# Modular and automated synthesis of oligonucleotide-small molecule conjugates for cathepsin B mediated traceless release of payloads†‡

**DOI:** 10.1039/d4cb00112e

**Published:** 2024-05-29

**Authors:** Cheng Jin, Siqi Li, Katherine A. Vallis, Afaf H. El-Sagheer, Tom Brown

**Affiliations:** a Department of Chemistry, Chemistry Research Laboratory, University of Oxford 12 Mansfield Road Oxford OX1 3TA UK tom.brown@chem.ox.ac.uk; b Hangzhou Institute of Medicine (HIM), Chinese Academy of Sciences, Zhejiang Cancer Hospital Hangzhou Zhejiang 310022 China jincheng@him.cas.cn; c Department of Oncology, University of Oxford Oxford OX3 7DQ UK; d School of Chemistry, University of Southampton Southampton SO17 1BJ UK; e Department of Science and Mathematics, Suez University, Faculty of Petroleum and Mining Engineering Suez 43721 Egypt

## Abstract

The reversible attachment of small molecules to oligonucleotides provides versatile tools for the development of improved oligonucleotide therapeutics. However, cleavable linkers in the oligonucleotide field are scarce, particularly with respect to the requirement for traceless release of the payload *in vivo*. Herein, we describe a cathepsin B-cleavable dipeptide phosphoramidite, Val-Ala(NB) for the automated synthesis of oligonucleotide-small molecule conjugates. Val-Ala(NB) was protected by a photolabile 2-nitrobenzyl group to improve the stability of the peptide linker during DNA synthesis. Intracellular cathepsin B digests the dipeptide efficiently, releasing the payload-phosphate which is converted to the free payload by endogenous phosphatase enzymes. With the advantages of modular synthesis and stimuli-responsive drug release, we believe Val-Ala(NB) will be a potentially valuable cleavable linker for use in oligonucleotide-drug conjugates.

## Abstract 2

An enzyme-cleavable dipeptide linker between an oligonucleotide and its payload tracelessly releases the payload in cells with applications in the rapidly expanding fields of oligonucleotide-drug conjugates and targeted oligonucleotide therapeutics.

## Graphical abstract

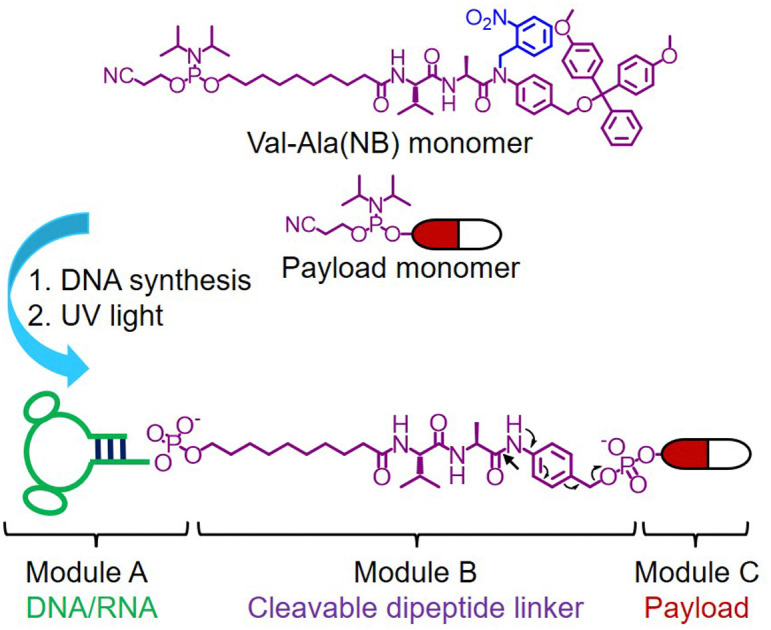

## Introduction

Solid-phase oligonucleotide synthesis is a well-established example of automated modular molecular synthesis. This technology enables the programmable assembly of oligonucleotides by the iterative addition of nucleoside phosphoramidites on resins.^1^ In addition to nucleosides, other small molecules can also be covalently attached to oligonucleotides in high yield through solid-phase phosphoramidite chemistry. These well-defined oligonucleotide-small molecule conjugates combine the functionality of small molecules with the sequence-specific target recognition of nucleic acids, expanding their potential practical uses. Fluorescent dyes/probes, hydrophobic molecules, targeting molecules and therapeutic reagents have all been conjugated to oligonucleotides for bioanalytical sensing,^2,3^ molecular assembly^4–6^ and drug development.^7–10^ In general, linkers between oligonucleotides and small molecules are non-cleavable and provide a stable connection. However, for oligonucleotide-drug conjugates in which small-molecule drugs or ligands must be conditionally released after entering the target cells, cleavable linkers are needed between the two entities.^11,12^

**Fig. 1:**
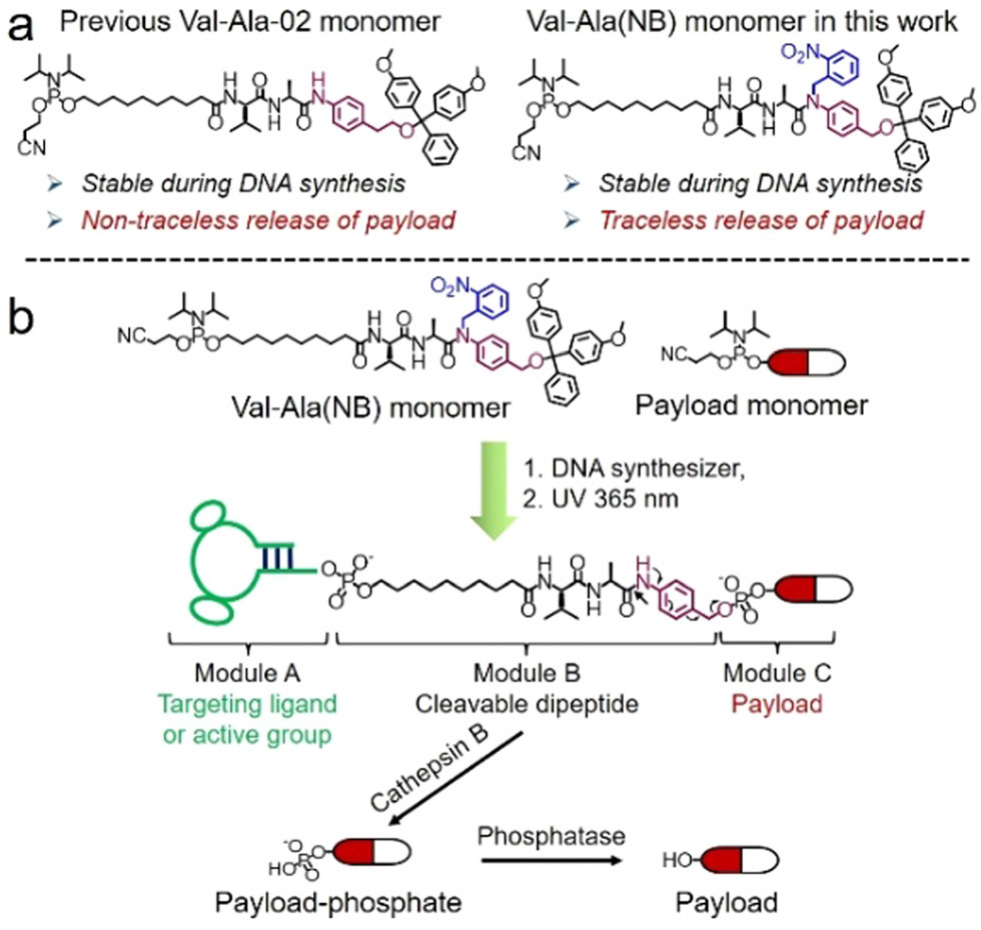
(a). The chemical structures of Val-Ala-02 and Val-Ala(NB) phosphoramidites. (b). The modular synthesis of oligonucleotide-small molecule conjugates using Val-Ala(NB) phosphoramidite on a DNA synthesizer, and the release of the payload-phosphate after cathepsin B-mediated enzymatic cleavage. The photolabile 2-nitrobenzyl on the Val-Ala(NB) monomer stabilizes the dipeptide during DNA synthesis.

Cleavable linkers are molecules that join two functional moieties through a scissile bond.^13^ Incorporation of cleavable linkers into therapeutic molecules confers the advantages of site-specific and stimulus-responsive cleavage.^14–17^ This enables the controllable release of a payload which can provide precision medicines and reduce side effects. However, commonly used cleavable linkages in the oligonucleotides field such as photocleavable (PC) and disulfide linkers have drawbacks for *in vivo* applications. These include the very limited tissue penetration of ultraviolet light and the poor stability of disulfide linkers during circulation in blood. Inspired by the cathepsin B-sensitive dipeptide linkers used in FDA-approved antibody–drug conjugates (ADCs),^18–20^ we recently reported the development of a Val-Ala-02 dipeptide linker phosphoramidite for the automated synthesis of enzyme-cleavable oligonucleotides Fig. 1a.^21^ In the Val-Ala-02 structure *p*-aminophenylethanol, instead of self-immolative *p*-aminobenzyl alcohol, was conjugated to the dipeptide moiety. Val-Ala-02 shows excellent stability during DNA synthesis but cannot be used for the traceless release of payloads as the *p*-aminophenethyl group remains attached to the payload after enzymatic cleavage of the dipeptide.

Herein, we overcome this problem by developing a novel traceless cleavable dipeptide linker phosphoramidite for the automated modular synthesis of oligonucleotide-small molecule conjugates. As shown in Fig. 1b, we synthesized a Val-Ala(NB) dipeptide phosphoramidite in which the dipeptide is protected by a photolabile 2-nitrobenzyl group to enhance its stability during DNA synthesis. After DNA synthesis, deprotection and photolysis, cathepsin B is able to cleave the Val-Ala dipeptide of oligonucleotide-payload conjugates. As a result, the payload-phosphate adduct is released, which is further converted to the dephosphorylated small molecule (free payload) by phosphatase enzymes. ODN7UV, a DNA conjugate with the microtubule destabilizing reagent combretastatin A-4 (CA4) attached *via* the Val-Ala linker, was delivered to HCT116 cells by lipofectamine. After transfection, we found a clear decline in cell viability which can be attributed to the release of CA4 *via* intracellular cathepsin B-mediated cleavage of the dipeptide. In addition, payloads containing an amino group can be conjugated to Val-Ala(NB) *via* a carbamate linkage through a two-step solid-phase process, expanding the general utility of Val-Ala(NB) phosphoramidite in the facile synthesis of oligonucleotide-small molecule conjugates.

## Results and discussion

**Fig. 2:**
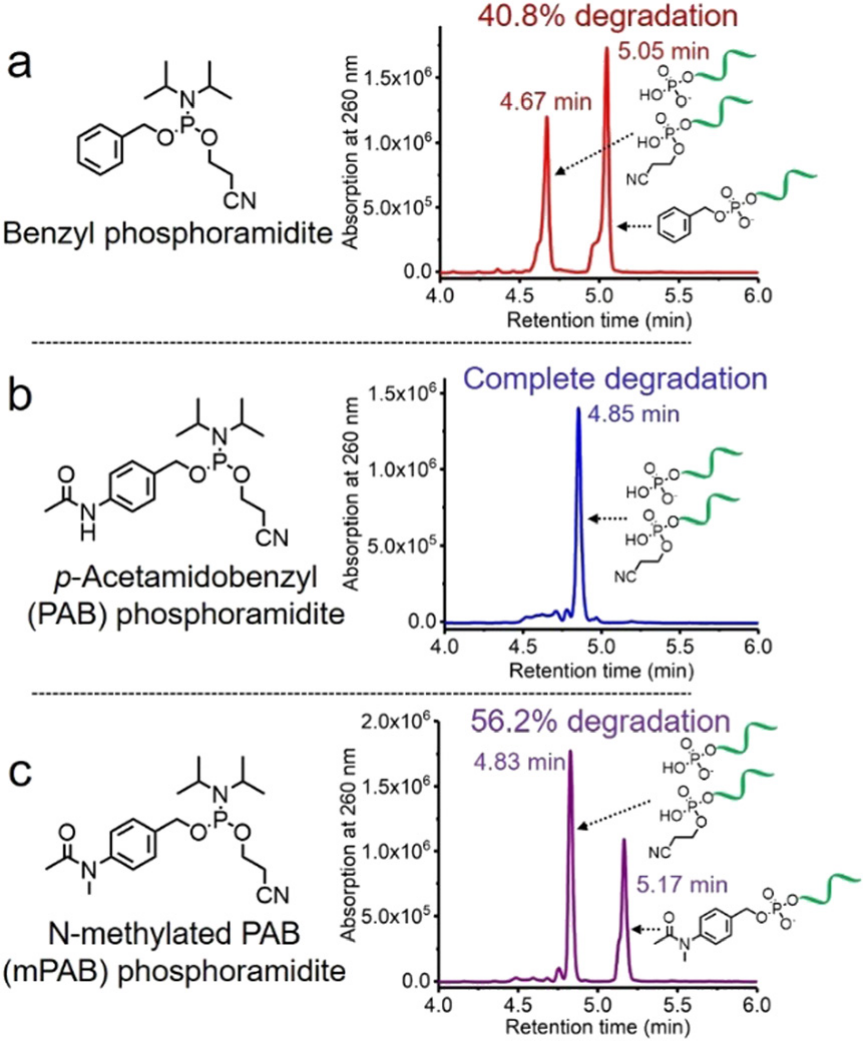
HPLC traces of (a) DNA-benzyl conjugate (ODN1), (b) DNA-PAB conjugate (ODN2) and (c) DNA-mPAB conjugate (ODN3) after deprotection by aqueous ammonia at room temperature for two hours. The percentages of degradation of ODN1, ODN2 and ODN3 are 40.8%, almost 100% and 56.2%, respectively, demonstrating that *N*-methylation of the PAB-phosphodiester enhances its stability during oligonucleotide synthesis and deprotection.

The solid-phase synthesis principle allows almost any small molecule to be straightforwardly attached to oligonucleotides on condition that (i) the corresponding phosphoramidite is available, and (ii) the small molecule is stable during oligonucleotide synthesis and deprotection.^22^ To develop our cathepsin B-cleavable dipeptide phosphoramidite for traceless payload release after enzymatic cleavage, a self-immolative spacer was designed to link the dipeptide to the small molecule. *p*-Aminobenzyl alcohol is a self-immolative linker that has been used in ADCs such as Adcetris.^23,24^ However, the *p*-acetamidobenzyl(PAB)-phosphodiester structure has poor stability during oligonucleotide synthesis/deprotection.^21^ We speculated that the acetamido group at the *para*-position leads to degradation of the PAB-phosphodiester. To evaluate this hypothesis, benzyl, PAB and *N*-methylated PAB (mPAB) phosphoramidites were synthesized and conjugated to DNA (Fig. 2). After oligonucleotide synthesis, the DNA conjugates were deprotected with concentrated aqueous ammonia at room temperature for two hours. As shown in Fig. 2b and Fig. S3 (ESI‡), consistent with the previous report, DNA-PAB conjugate (ODN2) with an acidic NH was completely degraded, and was even unstable to ultra-mild deprotection by 50 mM K_2_CO_3_ in methanol at room temperature for 4 hours (Fig. S4, ESI‡). However, the DNA-benzyl (ODN1) and DNA-mPAB (ODN3) conjugates have better stability, with 40.8% degradation for ODN1 and 56.2% degradation for ODN3 (Fig. 2a and c). These results indicate that the poor stability of the PAB-phosphodiester structure is improved by *N*-methylation of the PAB-phosphodiester (Fig. S5, ESI‡).

**Fig. 3:**
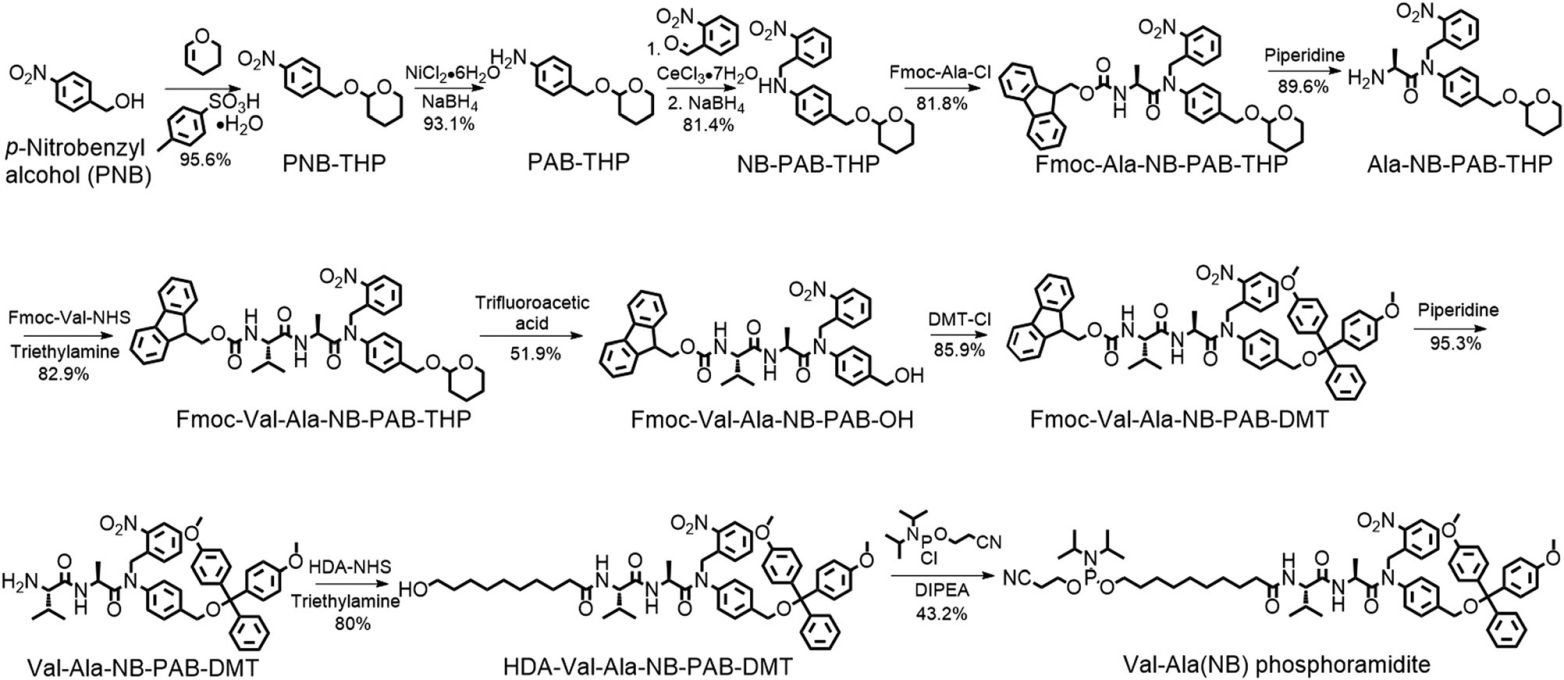
Synthesis of Val-Ala(NB) phosphoramidite. Fmoc-Ala-Cl: Fmoc-l-alanyl chloride. DMT-Cl: 4,4′-dimethoxytrityl chloride. HDA-NHS: 10-hydroxydecanoic acid NHS ester. DIPEA: *N,N*-diisopropylethylamine.

Encouraged by these results, we designed a Val-Ala(NB) phosphoramidite in which the dipeptide is protected by a photolabile 2-nitrobenzyl group to enhance stability. As shown in Fig. 3, *p*-nitrobenzyl alcohol (PNB) was protected with tetrahydropyran (THP) in 95.6% yield, and the resulting PNB-THP was reduced to PAB-THP by NaBH_4_ and nickel (II) chloride hexahydrate in an acetonitrile–water mixture. PAB-THP was then reacted with 2-nitrobenzaldehyde and reduced with NaBH_4_ to provide NB-PAB-THP in 81.4% yield. The NB-PAB-THP intermediate was coupled with Fmoc-l-alanyl chloride (Fmoc-Ala-Cl) to provide Fmoc-Ala-NB-PAB-THP in 81.8% yield, followed by deprotection of the Fmoc group with 20% piperidine in DMF. Reaction between Ala-NB-PAB-THP and Fmoc-Val-NHS ester provided Fmoc-Val-Ala-NB-PAB-THP in 82.9% yield. The THP group of Fmoc-Val-Ala-NB-PAB-THP was removed with 50% trifluoroacetic acid in dichloromethane, and the hydroxyl group of the purified Fmoc-Val-Ala-NB-PAB-OH was protected by reaction with 4,4′-dimethoxytrityl chloride (DMT-Cl) in anhydrous pyridine in 85.9% yield. After purification, Fmoc-Val-Ala-NB-PAB-DMT was treated with 20% piperidine in DMF, and the resultant Val-Ala-NB-PAB-DMT was further reacted with 10-hydroxydecanoic acid NHS ester (HDA-NHS). HDA-Val-Ala-NB-PAB-DMT was finally converted into the Val-Ala(NB) phosphoramidite for use in oligonucleotide synthesis (Fig. S75 and S76, ESI‡).

Next, an oligonucleotide with one Val-Ala(NB) incorporation (ODN4) was synthesized (Table S1, ESI‡). After synthesis, ODN4 was deprotected with concentrated aqueous ammonia at room temperature for two hours, followed by HPLC purification. As shown in Fig. S8 (ESI‡), 86.9% of the Val-Ala(NB) linkage remained intact, demonstrating that the 2-nitrobenzyl protecting group indeed improves the stability of the dipeptide. To determine whether Val-Ala(NB) could be degraded after longer deprotection times, Val-Ala(NB) was incorporated into DNA (ODN5) and deprotected with concentrated aqueous ammonia at 55 °C for 5 and 10 hours. The dipeptide showed good tolerance to both deprotection conditions; it was 85.1% intact after 5 hours and 86.1% intact after 10 hours (Fig. S9 and S10, ESI‡). This means that Val-Ala(NB) is stable during deprotection, and the slight degradation most likely occurs during DNA synthesis.

The photolabile 2-nitrobenzyl moiety is sensitive to exposure to ultraviolet (UV) light at 365 nm.^25^ Therefore, a 365 nm LED light was used to remove the 2-nitrobenzyl group from ODN4 in water.

**Fig. 4:**
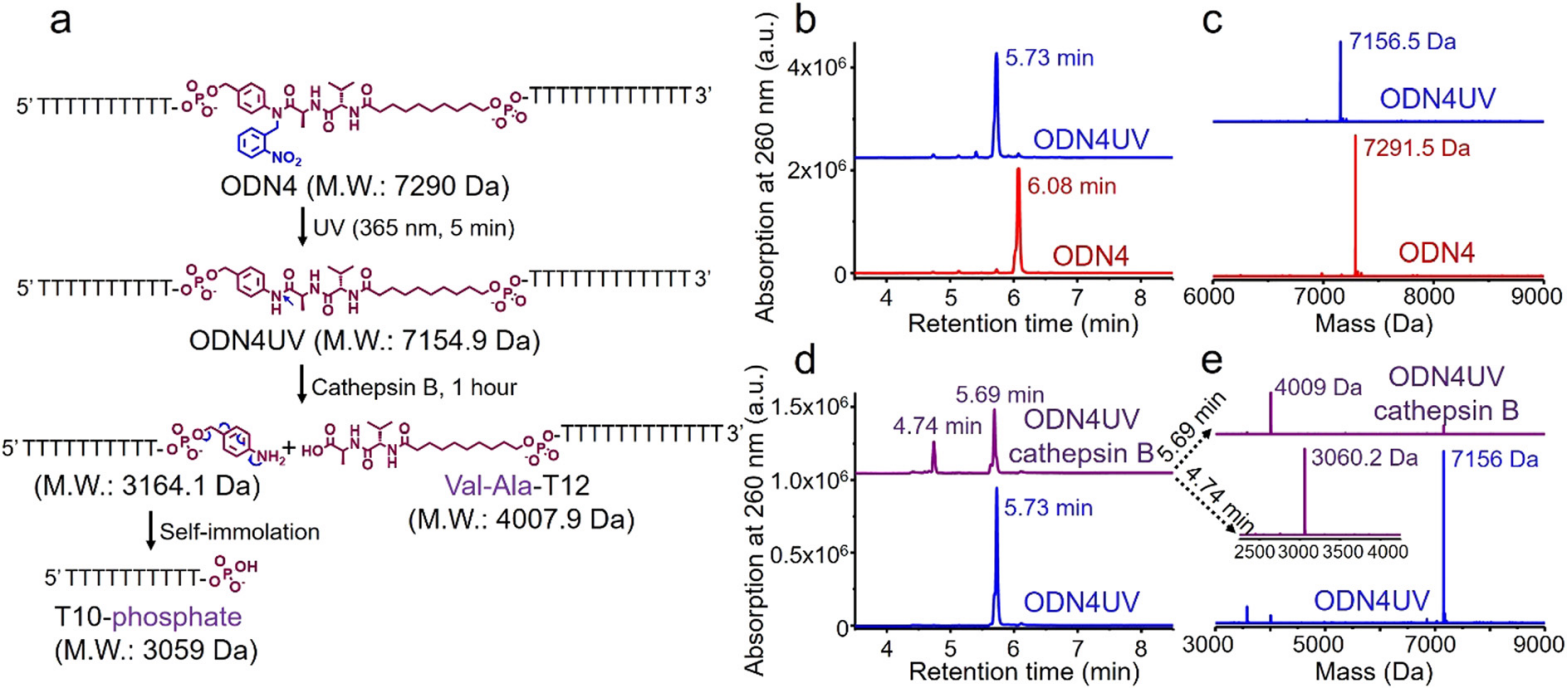
Photolysis of ODN4 and cathepsin B-mediated cleavage of the dipeptide in ODN4UV. (a) Reaction scheme for photolysis of ODN4 and cathepsin B-mediated cleavage of ODN4UV. (b) HPLC traces of ODN4 before (red line) and after (blue line) treatment with 365 nm UV light for 5 minutes. (c) Mass spectra of ODN4 before (red line) and after (blue line) treatment with 365 nm UV light for 5 minutes. (d) HPLC traces of 10 μM ODN4UV after incubation with (purple line) and without (blue line) 0.2 U mL^−1^ cathepsin B in buffer A for one hour at 37 °C. (e) Mass spectra of 10 μM ODN4UV after incubation with (purple lines) and without (blue line) 0.2 U mL^−1^ cathepsin B in buffer A for one hour at 37 °C.

As shown in Fig. 4b, after treatment with UV light for 5 minutes, the ODN4 peak at 6.08 minutes disappeared completely and a new DNA peak at 5.73 minutes emerged (Fig. 4b, blue line). The molecular weight of the new peak at 5.73 minutes is 7156.5 Da which is consistent with ODN4 without the 2-nitrobenzyl group (ODN4UV) (Fig. 4c), confirming that the 2-nitrobenzyl group had been removed. We then incubated 10 μM of ODN4UV with 0.2 U mL^−1^ of cathepsin B in 25 mM sodium acetate and 5 mM dithiothreitol (DTT) at pH 5.0 (buffer A) at 37 °C for 1 hour to investigate the enzymatic cleavage of the Val-Ala dipeptide. After incubation, the oligonucleotide was desalted and analyzed by mass spectrometry. As shown in Fig. 4d and e, ODN4UV shows excellent stability in buffer A after 1 hour of incubation (blue lines). However, after incubation with cathepsin B for 1 hour, ODN4UV was converted to T10-phosphate and Val-Ala-T12 (Fig. 4d and e, purple lines). These results demonstrate that cathepsin B cleaves ODN4UV into T10-phosphate and Val-Ala-T12. We further investigated the enzymatic cleavage kinetics of a 2 μM solution of Val-Ala dipeptide in FAM-labelled ODN4UV by 0.2 U mL^−1^ cathepsin B, and found that almost all the dipeptide was cleaved within two hours (Fig. S11, ESI‡).

**Fig. 5:**
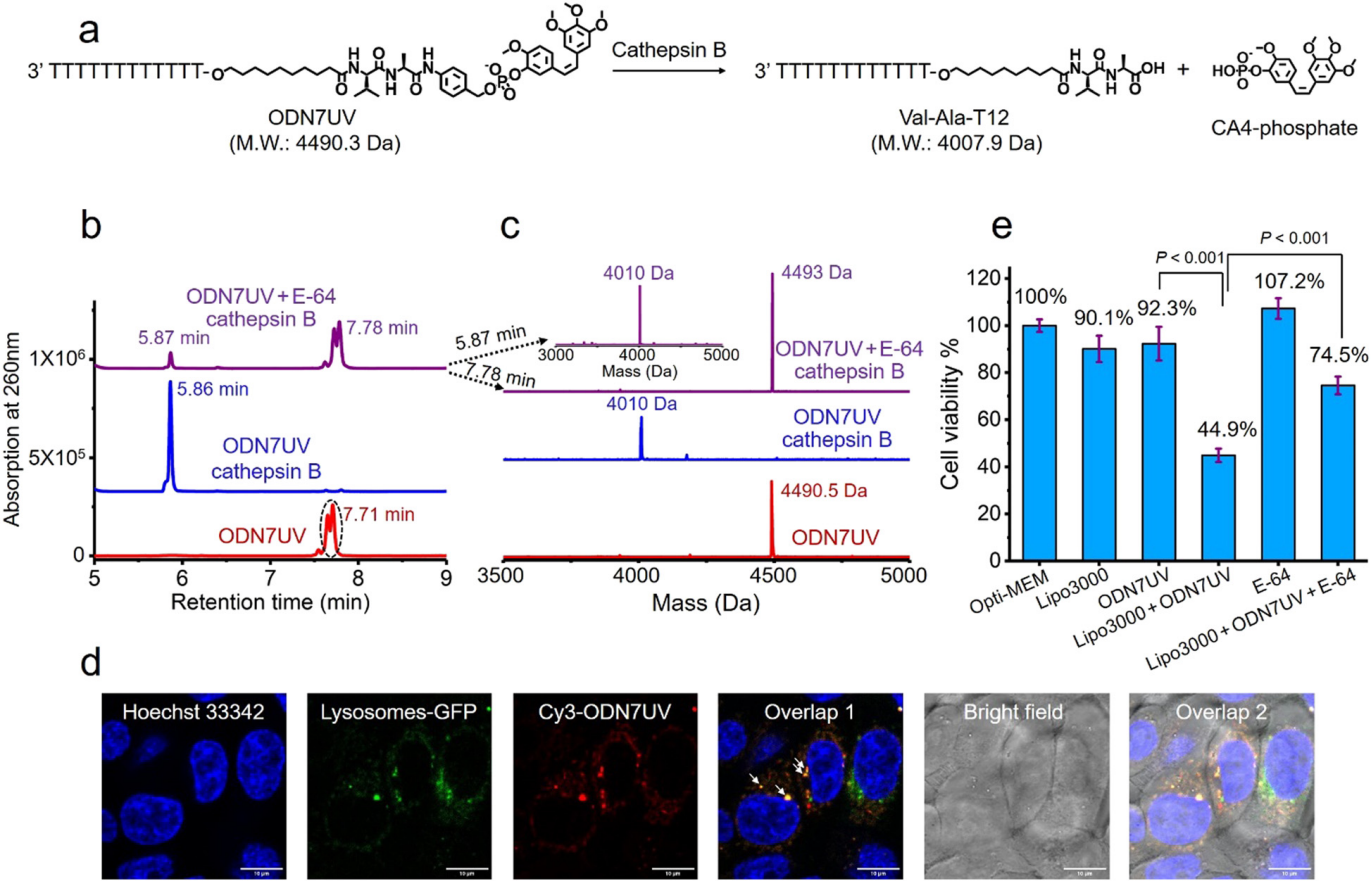
Intracellular cathepsin B-mediated cleavage of the dipeptide in ODN7UV. (a) Enzymatic cleavage of ODN7UV by cathepsin B. (b) HPLC traces of 10 μM of ODN7UV after incubation without cathepsin B (red line), with 0.2 U mL^−1^ of cathepsin B (blue line) or with 0.2 U mL^−1^ of cathepsin B and 1 μM E-64 (purple line) in buffer A at 37 °C for 1 hour. (c) Mass spectra of 10 μM of ODN7UV after incubation without cathepsin B (red line), with 0.2 U mL^−1^ of cathepsin B (blue line) or with 0.2 U mL^−1^ of cathepsin B and 1 μM of E-64 (purple line) in buffer A at 37 °C for 1 hour. After one hour of incubation, 10 μM of ODN7UV was completely cleaved by 0.2 U mL^−1^ of cathepsin B in buffer A. (d) Fluorescence co-localization imaging of HCT116 cells after incubation with Hoechst 33342 (blue), CellLight^TM^ lysosomes-GFP (green) and Lipo3000-transfected Cy3-ODN7UV (red) in Opti-MEM culture medium for 6 hours. The white arrows in Overlap 1 indicate colocalization of Cy3-ODN7UV with lysosomes-GFP. Scale bar: 10 μm. (e) Cell viability of HCT116 cells treated with Lipo3000, 1 μM of ODN7UV, Lipo3000-transfected ODN7UV (1 μM), 50 μM of E-64 and Lipo3000-transfected ODN7UV (1 μM) + 50 μM of E-64 in Opti-MEM culture medium for 48 hours.

Having confirmed the release of T10-phosphate from ODN4UV after enzymatic cleavage, we proceeded to construct oligonucleotide-small molecule conjugates with the cleavable dipeptide linkage. Various small molecules phosphoramidites can be quickly and efficiently attached *via* the Val-Ala(NB) linker to oligonucleotide sequences on a DNA synthesizer instead of resorting to complicated organic synthesis, and this is a key advantage of automated modular synthesis.^26,27^ CA4 is a microtubule destabilizing small-molecule drug^28^ (Fig. S12, ESI‡), and its phosphate derivative is a prodrug which is converted into the dephosphorylated compound by intracellular phosphatase. We synthesized CA4 phosphoramidite (Fig. S38, ESI‡) and covalently conjugated it to the dipeptide on ODN7 (Table S1, ESI‡). 10 μM of ODN7UV was incubated with 0.2 U mL^−1^ of cathepsin B in buffer A at 37 °C for 1 hour (Fig. 5a). The DNA peak of ODN7UV disappeared, and instead, a new DNA peak at 5.86 minutes was observed which corresponds to Val-Ala-T12 (Fig. 5b and c), demonstrating the enzymatic cleavage of ODN7UV by cathepsin B. Compound E-64, a commercial protease inhibitor, was then used to inhibit the activity of cathepsin B.^29^ As shown in Fig. 5b, 0.2 U mL^−1^ of cathepsin B failed to efficiently cut 10 μM ODN7UV in buffer A containing 1 μM E-64 (purple line), suggesting again, cathepsin B is responsible for the enzymatic cleavage of the dipeptide in ODN7UV. To investigate if the released payload-phosphate was further converted to the dephosphorylated compound by phosphatases, we conjugate 4-methylumbelliferone (4MU) to the peptide. 4MU is a coumarin fluorescent dye, and its phosphate derivative is a commercial fluorogenic probe for phosphatase enzymes.^30^ As shown in Fig. S13d (ESI‡), an obvious fluorescence emission at 454 nm was observed when 0.2 U mL^−1^ of cathepsin B and 0.2 U mL^−1^ acid phosphatase were added, indicating conversion of 4MU-posphate to 4MU. To investigate if the dipeptide is cleavable by cathepsin B in biological fluids, 5 μM of ODN6UV was treated with HCT116 cell lysate (final protein concentration 100 μg mL^−1^) in buffer A at 37 °C. After the addition of cell lysate, a clear increase in fluorescence was observed (Fig. S13e, ESI‡), indicating cleavage of the dipeptide by cathepsin B from the cell lysate. ODN6UV showed negligible cleavage in the buffer solution containing cell lysate if E-64 was added (Fig. S13e, cyan line, ESI‡), suggesting good biological stability of the phosphodiester linkage between the peptide and payload. These experiments support the conclusion that the successive cleavage of dipeptide-small molecule conjugates by cathepsin B and phosphatases results in the release of payloads.

Next, we investigated if the Val-Ala dipeptide attached to DNA can be cleaved by cathepsin B in cells. ODN7UV was transfected by lipofectamine into HCT116 cells, and the viability of the HCT116 cells was measured. Lipofectamine was used to deliver the Cy3-labeled ODN7UV into cellular lysosomes after 6 hours of transfection (Fig. 5d). Since intracellular cathepsin B largely localizes in lysosomes, delivery of ODN7UV to lysosomes by lipofectamine enables enzymatic cleavage of the dipeptide. As shown in Fig. 5e, after 48 hours incubation, the viability of HCT116 cells treated with lipofectamine-transfected ODN7UV (1 μM) is 44.9%, demonstrating the release of the microtubule destabilizing drug CA4-phosphate (Fig. S16, ESI‡). To further study if the intracellular release of CA4-phosphate from ODN7UV is dependent on the cathepsin B-mediated cleavage of the dipeptide, 50 μM of cell-penetrable E-64 was added to inhibit the activity of cathepsin B. As shown in Fig. 5e, the cell viability of HCT116 cells incubated with lipofectamine + ODN7UV + E-64 (50 μM) is 74.5% which is much greater than with lipofectamine + ODN7UV (44.9%). This result indicates that the intracellular release of CA4-phosphate from ODN7UV is dependent on the activity of intracellular cathepsin B.

**Fig. 6:**
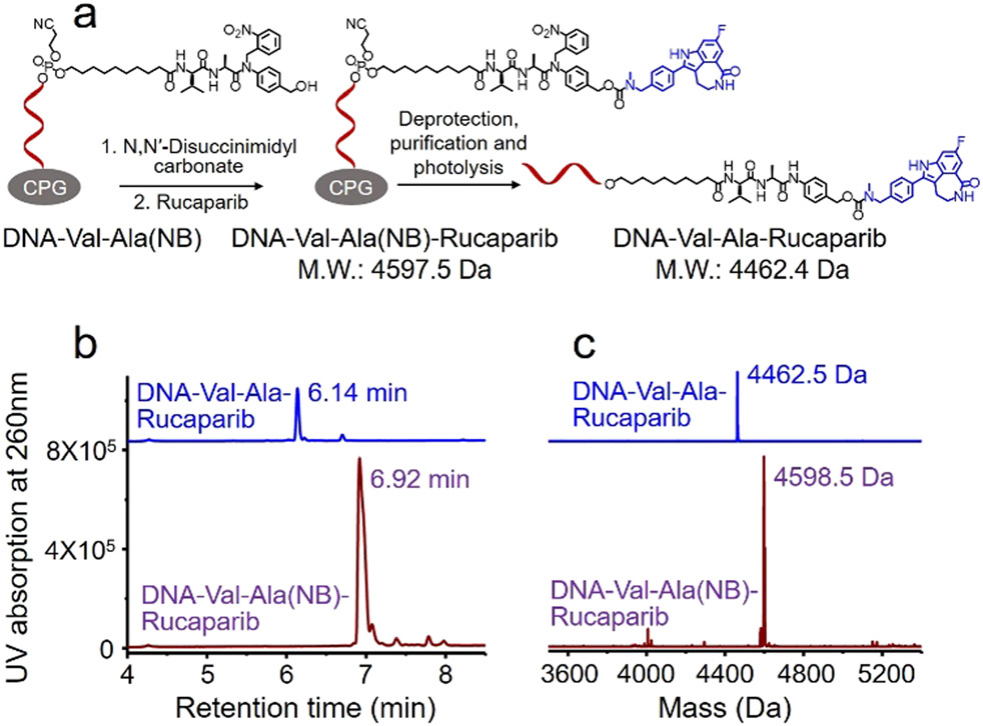
Solid-phase conjugation of the small-molecule anticancer drug rucaparib to the Val-Ala(NB) dipeptide linker through a carbamate linkage. (a) Activation of DNA-Val-Ala(NB) followed by conjugation with rucaparib on resin, then deprotection, purification and photolysis of DNA-Val-Ala(NB)-rucaparib. (b) HPLC traces of DNA-Val-Ala(NB)-rucaparib (purple) and DNA-Val-Ala-rucaparib (blue). (c) Mass spectra of DNA-Val-Ala(NB)-rucaparib (purple) and DNA-Val-Ala-rucaparib (blue).

Covalent conjugation of small molecules to the Val-Ala(NB) linker through phosphoramidite chemistry is limited to small molecules containing a hydroxyl group. To expand the general utility of the Val-Ala(NB) dipeptide linker to the synthesis of oligonucleotide-small molecule conjugates, we studied the solid-phase conjugation of the Val-Ala(NB) linker to payloads containing an amino group *via* a carbamate linker. As shown in Fig. 6a, the hydroxyl group of Val-Ala(NB) was activated by *N,N*′-disuccinimidyl carbonate at 37 °C overnight, followed by reaction with the secondary amino group of rucaparib.^31^ After washing, deprotection and purification, DNA-Val-Ala(NB)-rucaparib was obtained (Fig. 6b and c). The dipeptide in DNA-Val-Ala-rucaparib can be cleaved by cathepsin B, resulting in the traceless release of rucaparib. These experiments show that the covalent conjugation of small molecules containing an amino group to Val-Ala(NB) can be carried out on solid supports.

Many small-molecule drug candidates fail to advance due to their poor pharmacokinetics or unmanageable toxicity. One method to overcome these barriers is to covalently conjugate small-molecule drugs to targeting ligands for targeted delivery. There are several examples of ADCs, aptamer-drug conjugates (ApDCs) and small molecule-drug conjugates (SMDCs). Through the use of the Val-Ala(NB) monomer on a DNA synthesizer, sgc8-CA4, GalNAc-CA4 and NH_2_-DNA-CA4 conjugates were readily obtained (Fig. S31–S33, ESI‡). The NH_2_-DNA-CA4 conjugate contains an amino group which can be crosslinked to an antibody to synthesize ADCs (Fig. S18, ESI‡). These results further highlight the potential of Val-Ala(NB) in the development of targeting ligand-drug conjugates.

## Conclusions

In summary, a cathepsin B-cleavable Val-Ala(NB) phosphoramidite monomer has been developed for the efficient automated synthesis of oligonucleotide-small molecule conjugates. Cathepsin B releases small molecule-phosphate derivatives from oligonucleotides which are converted to the free small molecules by cellular phosphatase enzymes. To demonstrate the utility of our methodology, we have shown that the microtubule destabilizing reagent combretastatin A-4 (CA4) is released from an oligonucleotide after intracellular cathepsin B and phosphatase mediated cleavage of the dipeptide in lysosomes where cathepsin B largely localizes. To broaden the scope of the methodology to molecules that do not contain hydroxyl groups, we have carried out the solid-phase conjugation of small molecules containing amino groups to oligonucleotides *via* the Val-Ala(NB) linker by carbamate formation.

Overexpression of cathepsin B is associated with cancer and cathepsin B-sensitive peptides are widely used as cleavable linkers for the traceless release of therapeutic antibody–drug conjugates, *e.g.* DS-8201 and Adcetris. With this in mind, our results will encourage applications in the parallel field of therapeutic oligonucleotides for the traceless release of siRNA or ASOs from nanocarriers, targeting agents and molecules that promote cell uptake.

## Author contributions

C. J., A. H. E.-S. and T. B. designed the experiments. C. J. performed the experiments and collected the experimental data. A. H. E.-S. and C. J. synthesized oligonucleotides. S. L. and C. J. performed confocal fluorescence microscopy imaging experiments. C. J. and T. B. analyzed the data. C. J. wrote the manuscript, and T. B., A. H. E.-S., and K. A. V. revised the manuscript.

## Conflicts of interest

The authors declare no competing financial interest.

## Supplementary Material

CB-005-D4CB00112E-s001
