# Supplementary material for: Modular and automated synthesis of oligonucleotide-small molecule conjugates for cathepsin B mediated traceless release of payloads
Source: RSC Chem Biol. 2024 May 29;5(8):738–44. doi: 10.1039/d4cb00112e (PMC11289880; doi:10.1039/d4cb00112e)
Supplement: CB-005-D4CB00112E-s001 [file CB-005-D4CB00112E-s001.pdf]

Electronic Supplementary Material (ESI) for RSC Chemical Biology.
This journal is © The Royal Society of Chemistry 2024

Modular and Automated Synthesis of Oligonucleotide-Small Molecule Conjugates for Cathepsin B Mediated Traceless Release of Payloads

Cheng Jin[a,b]*, Siqi Li[a,c], Katherine A. Vallis[c], Afaf H. El-Sagheer[a,d,e] and Tom Brown[a]*

a Department of Chemistry, Chemistry Research Laboratory, University of Oxford, 12 Mansfield Road, Oxford, OX1 3TA, United Kingdom

b Hangzhou Institute of Medicine (HIM), Chinese Academy of Sciences, Zhejiang Cancer Hospital, Hangzhou, Zhejiang 310022, China

c Department of Oncology, University of Oxford, Oxford, OX3 7DQ, United Kingdom

d School of Chemistry, University of Southampton, Southampton, SO17 1BJ, UK

e Department of Science and Mathematics, Suez University, Faculty of Petroleum and Mining Engineering, Suez, 43721, Egypt

*Corresponding authors: jincheng@him.cas.cn (C.J.) and tom.brown@chem.ox.ac.uk (T.B.)

Contents

1. Materials 1
2. Experimental section 1
2.1 Oligonucleotide synthesis 1
2.2 Photolysis 2
2.3 Enzymatic cleavage of dipeptide by cathepsin B 2
2.4 Fluorescence spectra experiments 2
2.5 Fluorescence analysis of ODN6UV in cell lysate 2
2.6 Cell viability assay 3
2.7 Confocal fluorescence microscopy imaging 3
2.8 Solid-phase conjugation of Val-Ala(NB) with amino compound 3
3. Figures and Tables 4
4. Synthesis of phosphoramidites 23
4.1 Synthesis of benzyl phosphoramidite 24
4.2 Synthesis of PAB phosphoramidite 24
4.3 Synthesis of N-methylated PAB (mPAB) phosphoramidite 26
4.4 Synthesis of Val-Ala(NB) phosphoramidite 28
4.5 Synthesis of CA4 phosphoramidite 34
4.6 Synthesis of 4MU phosphoramidite 35
5. NMR spectra 35
6. References 57

1. Materials

All chemicals for organic synthesis were purchased commercially without further treatment. Combretastatin A-4 (CA4) was purchased from Fluorochem. 5'-GalNAc C3 phosphoramidite and 3'-amino modifier C7 CPG were purchased from Glen Research. Cathepsin B from human placenta, E-64 protease inhibitor, acid phosphatase from potato, cell counting kit-8 (CCK-8), Rucaparib camsylate and 4-methylumbelliferone (4MU) were purchased from Sigma-Aldrich. CellLight^{TM} Lysosomes-GFP BacMam 2.0, Lipofectamine^{TM} 3000 Transfection Reagent and Opti-MEM^{TM} I Reduced Serum Medium were purchased from Thermo Fisher Scientific.

2. Experimental section

2.1 Oligonucleotide synthesis

All oligonucleotides were synthesized on an ABI 394 DNA/RNA synthesizer with standard protocol. Val-Ala(NB) phosphoramidite was dissolved in anhydrous dichloromethane in concentration of 0.1 mol/L. Benzyl alcohol (Benzyl), N-(4-(hydroxymethyl)phenyl)acetamide (PAB), N-(4-(hydroxymethyl)phenyl)-N-methylacetamide (mPAB), Combretastatin A-4 (CA4), 4-methylumbelliferone (4MU) and 5'-GalNAc C3 (GalNAc) phosphoramidites were dissolved in anhydrous acetonitrile in concentration of 0.1 mol/L, respectively. After oligonucleotide synthesis, DNA were deprotected in concentrated aqueous ammonia at room temperature for 2 hours. For sgc8-CA4 conjugate, DNA were deprotected in concentrated aqueous ammonia at 55 °C for 10 hours. After deprotection, the ammonia was removed under high vacuum, and the DNA was purified using of high-performance liquid chromatography (HPLC). For ODN4 and ODN5 (Table S1), 80% aqueous acetic acid was used to remove 4,4'-dimethoxytrityl (DMT) group from DNA (30 minutes, room temperature) after HPLC purification. Then, the purified DNA was freeze dried and desalted, and quantitated by detecting their absorbance at 260 nm.

2.2 Photolysis

365 nm LED light (Thorlabs M365LP1) was used to remove 2-nitrobenzyl group from DNA. The desalted DNA was exposure to 365 nm LED light for 5 minutes at room temperature in water. Then, DNA was subjected to HPLC for further purification.

2.3 Enzymatic cleavage of dipeptide by cathepsin B

Cathepsin B from human placenta (2 U) (Sigma-Aldrich) was dissolved in 200 μL activation buffer (25 mM sodium acetate, 5 mM dithiothreitol (DTT), 1 mM ethylenediaminetetraacetic acid (EDTA), pH 5.0) to the final concentration of 10 U/mL. For enzymatic cleavage, 10 μM DNA was dissolved with 200 μL buffer A (25 mM sodium acetate, 5 mM DTT, pH 5.0) and incubated with 0.2 U/mL cathepsin B at 37 °C for one hour. After incubation, the DNA solution was desalted using of NAP columns (GE Healthcare), and the desalted DNA was analyzed by electrospray mass spectrometry (ES-).

2.4 Fluorescence spectra experiments

ODN6UV was dissolved in buffer A and then incubated with cathepsin B and acid phosphatase for enzymatic cleavage study. ODN6UV (2 μM) was incubated with 0.2 U/mL cathepsin B in buffer A at 37 °C for 1 hour. Then, 0.2 U/mL acid phosphatase was added, and the mixture was incubated at 37 °C for 1 hour. Then, the mixture was subjected to fluorescence spectra detection (PerkinElmer LS-50B Luminescence Spectrophotometer). Excitation wavelength: 360 nm, slit width: 7 nm; emission wavelength: 380 to 650 nm, slit width: 7 nm.

2.5 Fluorescence analysis of ODN6UV in cell lysate

Preparation of HCT116 cell lysate. HCT116 cells were cultured in 6-well cell culture plate to about 90% confluency. The DMEM culture medium was removed, and cells were washed with 1 mL Dulbecco's phosphate-buffered saline (DPBS) twice. After washing, 600 μL cell lysis buffer (10 mM Tris-HCl buffer, 50 mM KCl, 1.5 mM $MgCl_2$, 0.45% Tween 20 and 0.45% Triton X-100, pH 8.5) was added to the each well, followed by vibration at 400 rpm for 5 min. The mixture was collected and centrifuged at 8000 rpm for 5 min. The supernatant was collected, and the protein was quantitated by Pierce™ BCA protein assay kit (Thermo Fisher Scientific). The protein concentration of the supernatant is 1993.2 μg/mL.

5 μM ODN6UV was incubated with HCT116 cell lysate (the final protein concentration is 100 μg/mL) in buffer A at 37 °C for various time intervals. For the control group, 5 μM ODN6UV was incubated with HCT116 cell lysate and 10 μM E-64 in buffer A at 37 °C. The fluorescence intensity was measured at 450 nm on a microplate reader (INFINITE M1000 PRO). Excitation wavelength: 360 nm, emission wavelength: 450 nm, both slit widths were set as 20 nm.

2.6 Cell viability assay

HCT116 cells were cultured in DMEM culture media with 10% FBS, and 0.1 U/mL penicillin and 0.1 mg/mL streptomycin under 5% CO_2 atmosphere. For cell viability assay, 3000 HCT116 cells were cultured in the each well of 96-well cell culture plate and were grew for 24 hours in complete DMEM medium before incubation. Then, DMEM medium was removed, and 100 μL DPBS was added to wash cells. Then, cells were incubated with lipofectamine-transfected ODN7UV in Opti-MEM cell culture medium for 48 hours. After incubation, 10 μL cell counting kit-8 (CCK-8) was added. The cell viability was measured by detecting their absorbance at 450 nm on a microplate reader (INFINITE M1000 PRO).

Preparation of lipofectamine-ODN7UV transfection complex. 0.6 μL lipofectamine 3000 was diluted with 50 μL Opti-MEM cell culture medium (solution A), and 0.1 nmol ODN7UV was diluted with another 50 μL Opti-MEM cell culture medium and 0.4 μL P3000 reagent was added (solution B). Solutions A and B were mixed, and the mixture was stand at room temperature for 15 minutes before incubation with HCT116 cells.

2.7 Confocal fluorescence microscopy imaging

HCT116 cells were cultured in 10-wells microscope slide in 200 μL complete DMEM culture medium, and CellLight^{TM} Lysosomes-GFP (8 μL) was added. After 24 hours, DMEM culture medium was removed, and the cells were washed twice with DPBS. Then, cells were incubated with Cy3-ODN7UV (1 μM) or lipofectamine-transfected Cy3-ODN7UV (1 μM) in Opti-MEM culture medium for 6 hours. Hoechst 33342 (1 μg/ml) was incubated with cells for 10 minutes to stain the cell nucleus. After incubation, Opti-MEM culture medium was removed, and the cells were washed twice with DPBS and then subjected to confocal fluorescence microscopy imaging.

Preparation of lipofectamine/Cy3-ODN7UV transfection complex. Lipofectamine 3000 (0.6 μL) was diluted with 100 μL Opti-MEM culture medium (solution C). 0.2 nmol Cy3-ODN7UV was dissolved with 100 μL Opti-MEM culture medium and 0.4 μL P3000 reagent was added (solution D). Solutions C and D were mixed, and the mixture was stand for 15 minutes at room temperature before incubation with HCT116 cells.

2.8 Solid-phase conjugation of Val-Ala(NB) with amino compound

Resins of DNA-Val-Ala(NB) (DMT-off, 5' Val-Ala(NB)-TTTTTTTTTTTT 3') were dried with argon gas before conjugation reaction. Then, DNA-Val-Ala(NB) (0.5 μmol) resins were reacted with N,N'-disuccinimidyl carbonate (65 mg, 250 μmol) dissolved in 2.7 mL anhydrous CH_3CN and 0.3 mL anhydrous DIPEA at 37 °C for 5 hours. After washing with anhydrous CH_3CN, the resins were further reacted with 6-amino-1-hexanol (14 mg, 120 μmol) dissolved in 1.8 mL CH_3CN, 0.3 mL DMSO and 0.2 mL DIPEA at 37 °C overnight. Then, the resins were washed with CH_3CN and dried by argon gas. The oligonucleotide was deprotected by 50 mM K_2CO_3 in methanol (3 mL) at 25 °C for 4 hours. After deprotection, 18 μL acetic acid was added to neutralize the solution. The solvents were removed under high vacuum, and the DNA was purified by HPLC and analyzed by electrospray mass spectrometry (ES-).

After activation with N,N'-disuccinimidyl carbonate, 0.5 μmol DNA-Val-Ala(NB) resins were reacted with Rucaparib camsylate (3 mg, 5.4 μmol) dissolved in 1.5 mL DMSO, 0.5 mL anhydrous CH_3CN and 0.1 mL DIPEA at 37 °C overnight. Then, the resins were washed with CH_3CN and dried by argon gas, and the DNAs were deprotected. After purification and photolysis, DNA-Val-Ala(NB)-Rucaparib and DNA-Val-Ala-Rucaparib were analyzed by electrospray mass spectrometry (ES-).

3. Figures and Tables

Table S1. Designed oligonucleotides in this work

Name	Sequences (from 5' to 3')
ODN1	Benzyl-TTT TTT TTT TTT
ODN2	PAB-TTT TTT TTT TTT
ODN3	mPAB-TTT TTT TTT TTT
ODN4	TTT TTT TTT T-Val-Ala(NB)-TTT TTT TTT TTT
ODN4UV	TTT TTT TTT T-Val-Ala-TTT TTT TTT TTT
FAM-ODN4	FAM-TTT TTT TTT T-Val-Ala(NB)-TTT TTT TTT TTT
FAM-ODN4UV	FAM-TTT TTT TTT T-Val-Ala-TTT TTT TTT TTT
ODN5	T-Val-Ala(NB)-TTT TTT TTT TTT
ODN5UV	T-Val-Ala-TTT TTT TTT TTT
ODN6	4MU-Val-Ala(NB)-TTT TTT TTT TTT
ODN6UV	4MU-Val-Ala-TTT TTT TTT TTT
ODN7	CA4-Val-Ala(NB)-TTT TTT TTT TTT
ODN7UV	CA4-Val-Ala-TTT TTT TTT TTT
Cy3-ODN7	CA4-Val-Ala(NB)-TTT TTT TTT TTT-Cy3
Cy3-ODN7UV	CA4-Val-Ala-TTT TTT TTT TTT-Cy3
Sgc8-CA4	CA4-Val-Ala-TTT ATC TAA CTG CTG CGC CGC CGG GAA AAT ACT GTA CGG TTA GA
GalNAc-CA4	CA4-Val-Ala-$U_{OMe}U_{OMe}U_{OMe}U_{OMe}U_{OMe}U_{OMe}$-GalNAc-GalNAc-GalNAc-T
NH_2-DNA-CA4	CA4-Val-Ala-TTT TTT TTT TTT TTT-NH_2

Note: Val-Ala indicates dipeptide without 2-nitrobenzyl protection. U_{OMe} indicates 2'-OMe-U. NH_2 indicates 3'-amino C7 modification. GalNAc indicates 5'-GalNAc C3 modification.

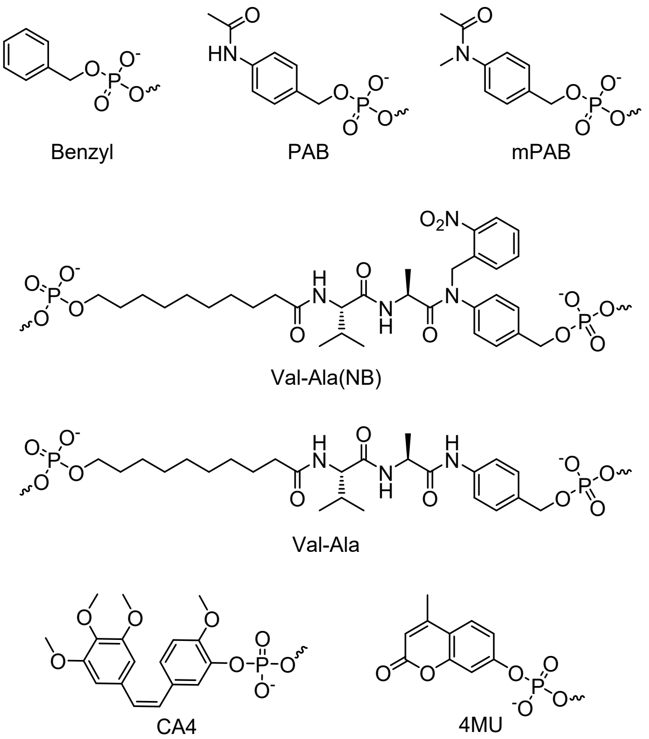

Figure S1. Chemical structures of Benzyl, PAB, mPAB, Val-Ala(NB), Val-Ala, CA4 and 4MU modifications on oligonucleotides.

Table S2. Calculated and found mass of DNA

Name	Calculated mass (Da)	Found mass (Da)
ODN4	7290	7291.5
ODN4UV	7154.9	7156.5
FAM-ODN4	7826.7	7827.4
FAM-ODN4UV	7692.5	7693
ODN5	4552.3	4553
ODN5UV	4417.2	4419
ODN6	4486.2	4489.5
ODN6UV	4351.1	4353.5
ODN7	4625.4	4626
ODN7UV	4490.3	4491
Cy3-ODN7	5132	5133.6
Cy3-ODN7UV	4998.9	5000
Sgc8-CA4	14448.7	14450.5
GalNAc-CA4	4880.5	4881
NH_2-DNA-CA4	5614.6	5613.1

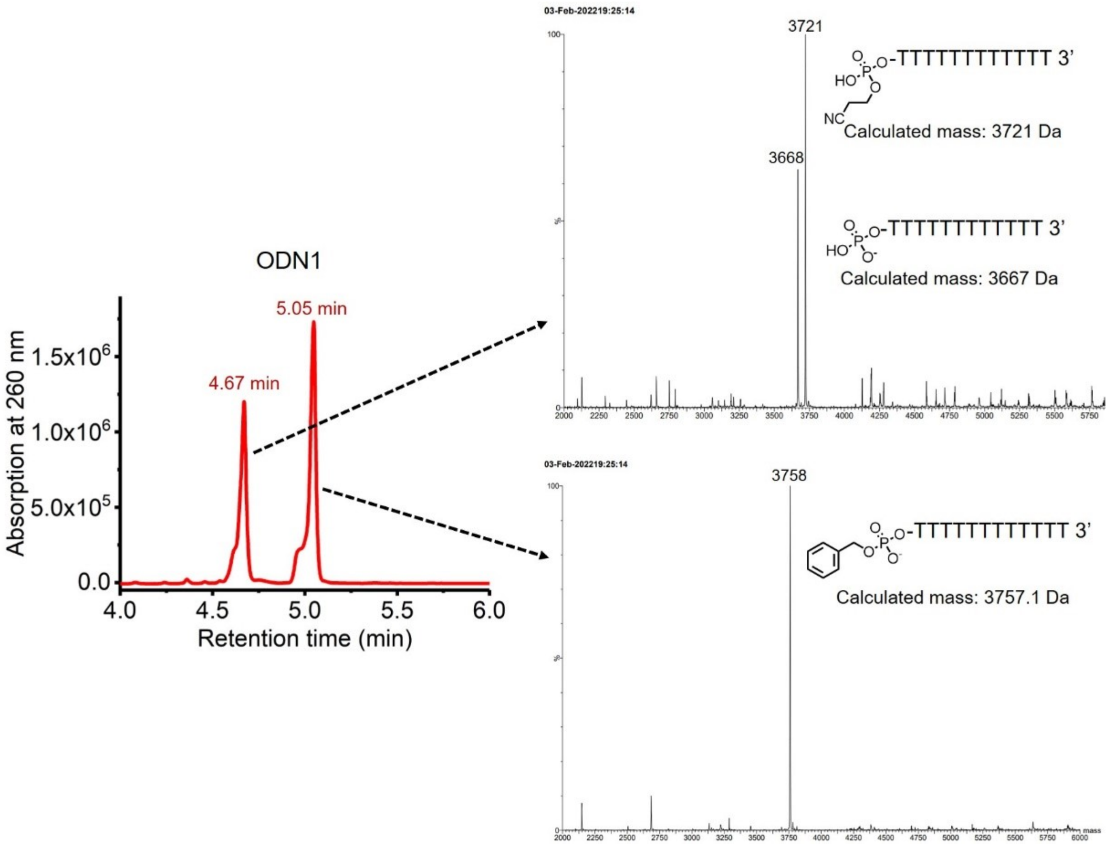

Figure S2. HPLC trace (left) and mass spectra (right) of ODN1 after deprotection by concentrated aqueous ammonia at room temperature for two hours. DNA peaks at 4.67 and 5.05 minutes are degraded and successful DNA, respectively.

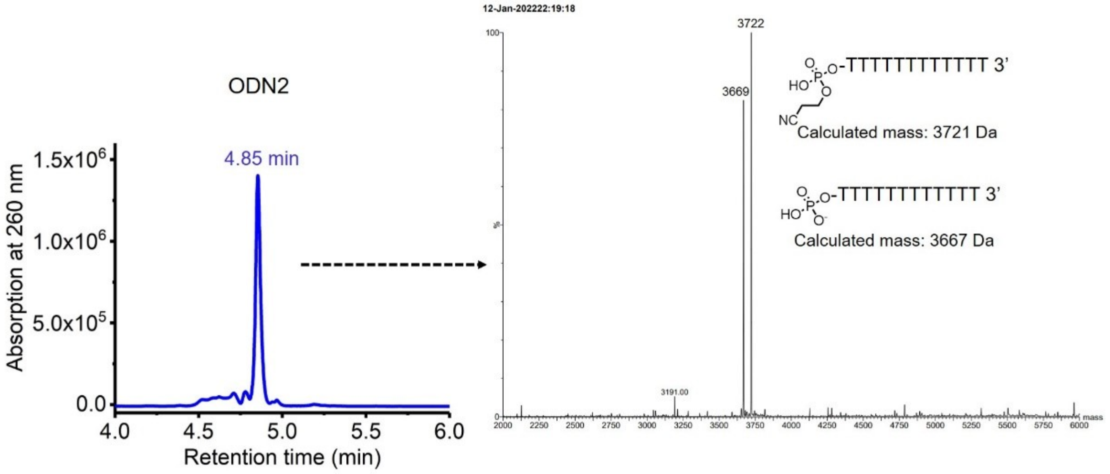

Figure S3. HPLC trace (left) and mass spectrum (right) of ODN2 after deprotection by concentrated aqueous ammonia at room temperature for two hours. ODN2 was completely degraded during oligonucleotide synthesis and deprotection.

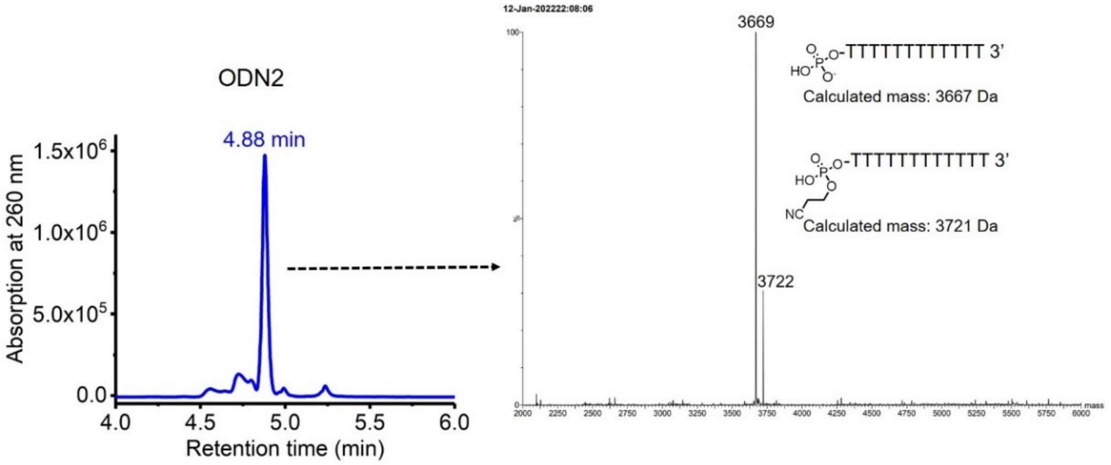

Figure S4. HPLC trace (left) and mass spectrum (right) of ODN2 after deprotection by 50 mM anhydrous K_2CO_3 in methanol at room temperature for 4 hours. After deprotection by 50 mM K_2CO_3 in methanol (2 mL), 12 µL of acetic acid were added to neutralize the solution. Then, the solvent was removed under high vacuum. ODN2 was completely degraded during oligonucleotide synthesis and deprotection.

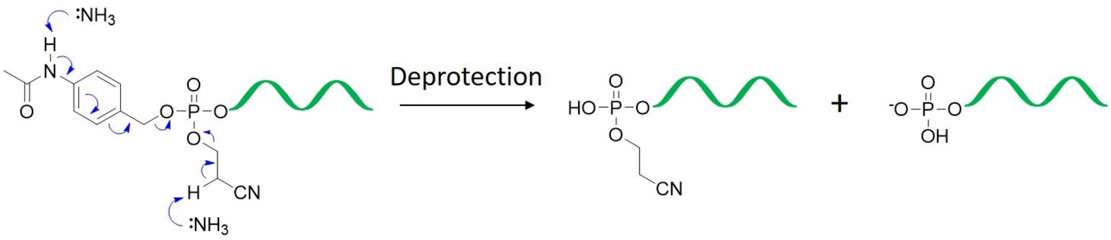

Figure S5. Proposed mechanism of rapid degradation of ODN2 during deprotection after solid-phase oligonucleotide synthesis.

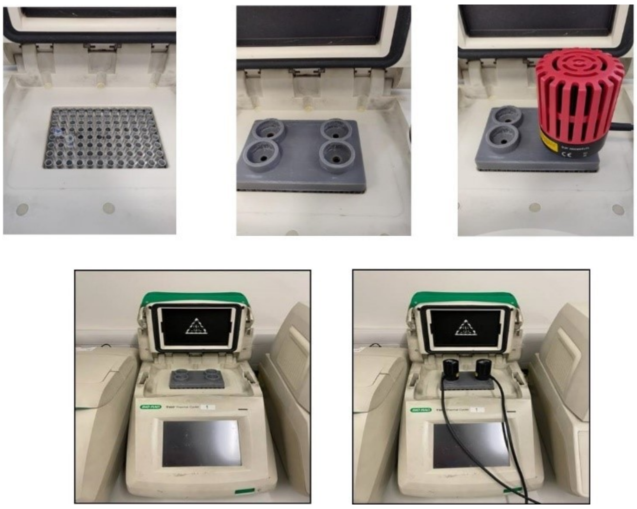

Figure S6. The UV cleavage apparatus used in this work. Using 3D-printing we have constructed a simple and inexpensive UV-irradiation device that sits on 96-well plates (or can be adapted to other formats). The device holds sample tubes at a fixed distance from the lamp to ensure consistent results. Between one and four LEDs of different wavelength can be used simultaneously (one and two lamps shown in the figure). In the present work just a single UV light source is required. Other designs can be readily constructed, and commercial systems are also available.

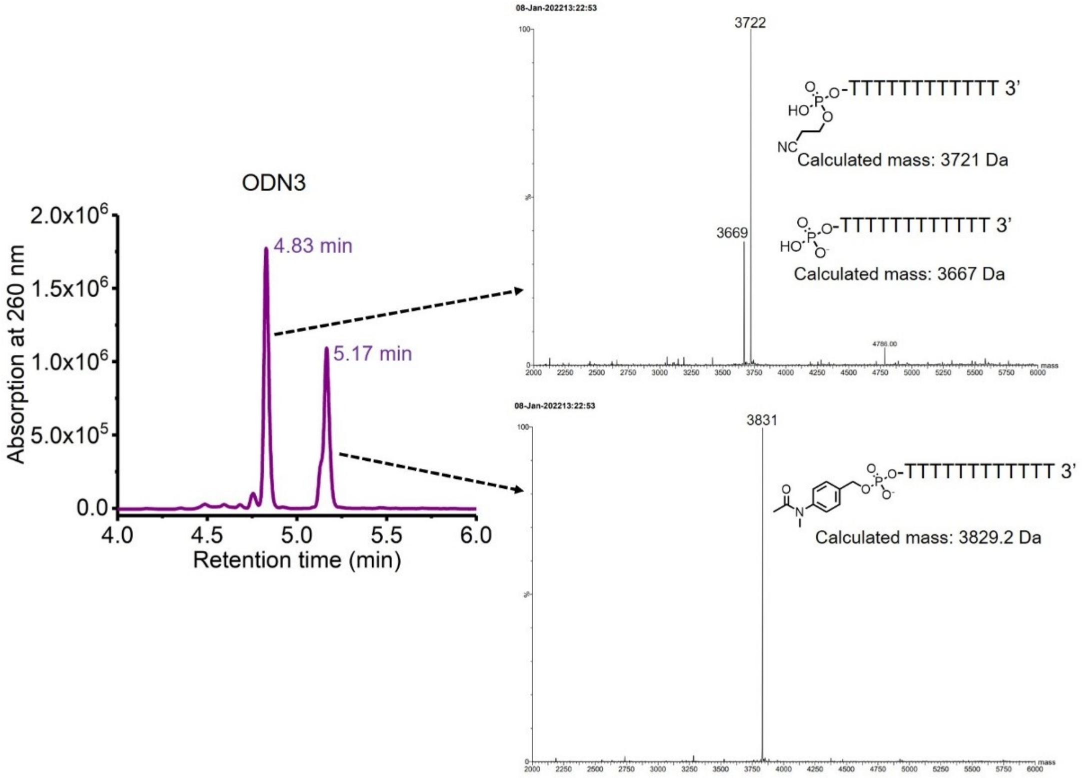

Figure S7. HPLC trace (left) and mass spectra (right) of ODN3 after deprotection by concentrated aqueous ammonia at room temperature for two hours. DNA peaks at 4.83 and 5.17 minutes are degraded and successful DNA, respectively.

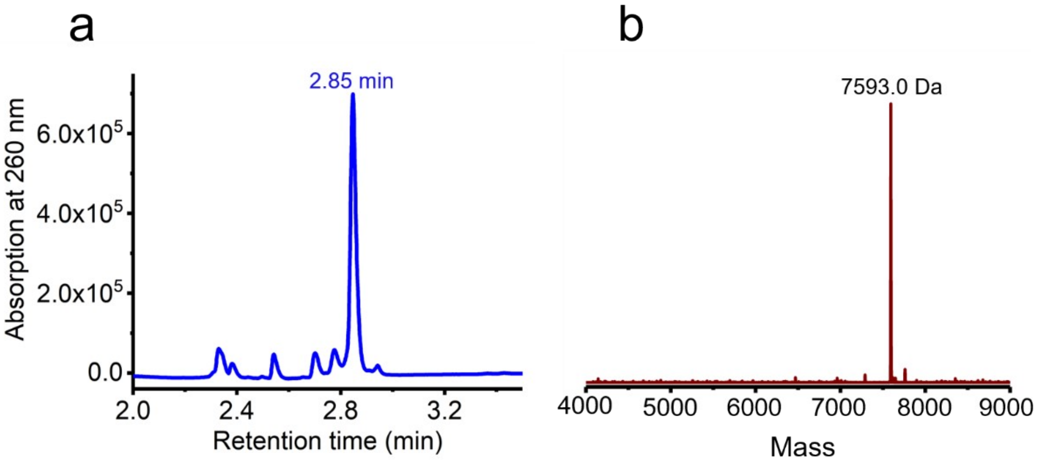

Figure S8. HPLC trace and mass spectrum of DMT-protected ODN4 after deprotection. (**a**) HPLC trace of DMT-protected ODN4 after deprotection by concentrated aqueous ammonia at room temperature for two hours. DNA peak at 2.85 min is the successful DMT-protected ODN4: 5’ DMT-TTTTTTTTTT-Val-Ala(NB)-TTTTTTTTTTTT 3’. After calculation, 86.9% Val-Ala(NB) on ODN4 remained intact after deprotection. (**b**) Mass spectrum of DNA peak at 2.85 min in Figure S6a. The calculated molecular weight is 7593 Da, and the observed molecular weight is 7593 Da.

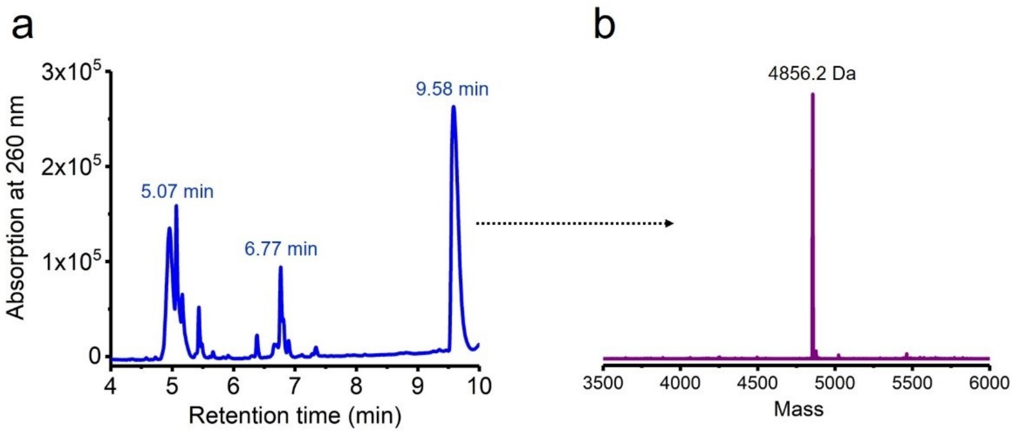

Figure S9. HPLC trace and mass spectrum of DMT-protected ODN5 (5’ DMT-T-Val-Ala(NB)-TTTTTTTTTTTT 3’) after 5 hours of deprotection. (**a**) HPLC trace of DMT-protected ODN5 after deprotection by concentrated aqueous ammonia at 55 °C for 5 hours. DNA peak around 5.07 min is DNA without peptide (5’ TTTTTTTTTTTT 3’). DNA peak at 6.77min is DNA with degraded peptide (5’ phosphite-Val-Ala(NB)-TTTTTTTTTTTT 3’). DNA peak at 9.58 min is the successfully synthesized DNA (5’ DMT-T-Val-Ala(NB)-TTTTTTTTTTTT 3’). Calculating the peak area of DNA at 6.77 and 9.58 min, 85.1% Val-Ala(NB) remained intact after deprotection. (**b**) Mass spectrum of DNA peak at 9.58 min in Figure S7a. The calculated molecular weight is 4854.7 Da, and the observed molecular weight is 4856.2 Da.

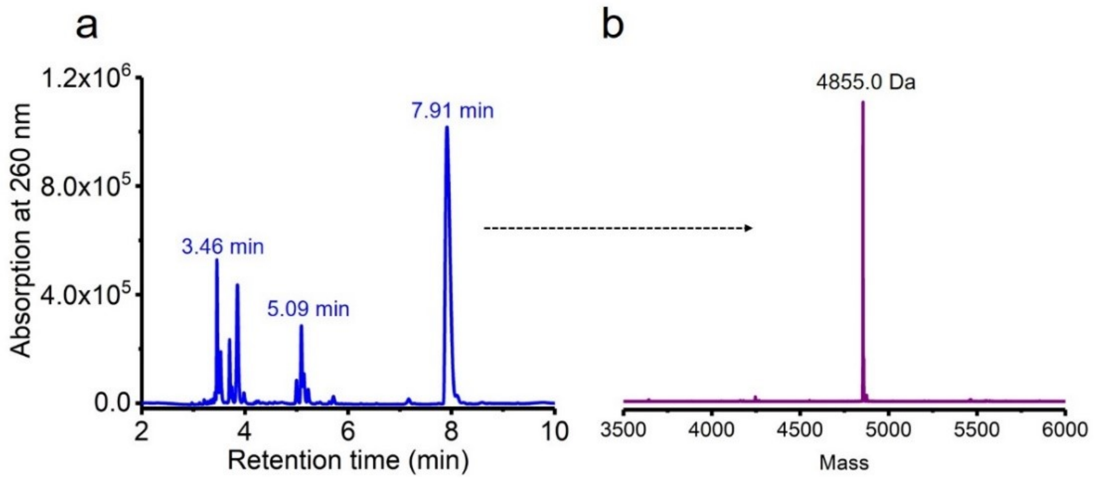

Figure S10. HPLC trace and mass spectrum of DMT-protected ODN5 (5' DMT-T-Val-Ala(NB)-TTTTTTTTTTTT 3') after 10 hours of deprotection. (**a**) HPLC trace of DMT-protected ODN5 after deprotection by concentrated aqueous ammonia at 55 °C for 10 hours. DNA peak around 3.46 min is the DNA without peptide (TTTTTTTTTTTTT). DNA peak at 5.09 min is the DNA with degraded peptide (5' phosphite-Val-Ala(NB)-TTTTTTTTTTTT). DNA peak at 7.91 min is the successfully synthesized DNA (5' DMT-T-Val-Ala(NB)-TTTTTTTTTTTT 3'). Calculating the peak area of DNA at 5.09 and 7.91 min, 86.1% Val-Ala(NB) on ODN5 remained intact after deprotection. (**b**) Mass spectrum of DNA peak at 7.91 min in Figure S8a. The calculated molecular weight is 4854.7 Da, and the observed molecular weight is 4855.0 Da.

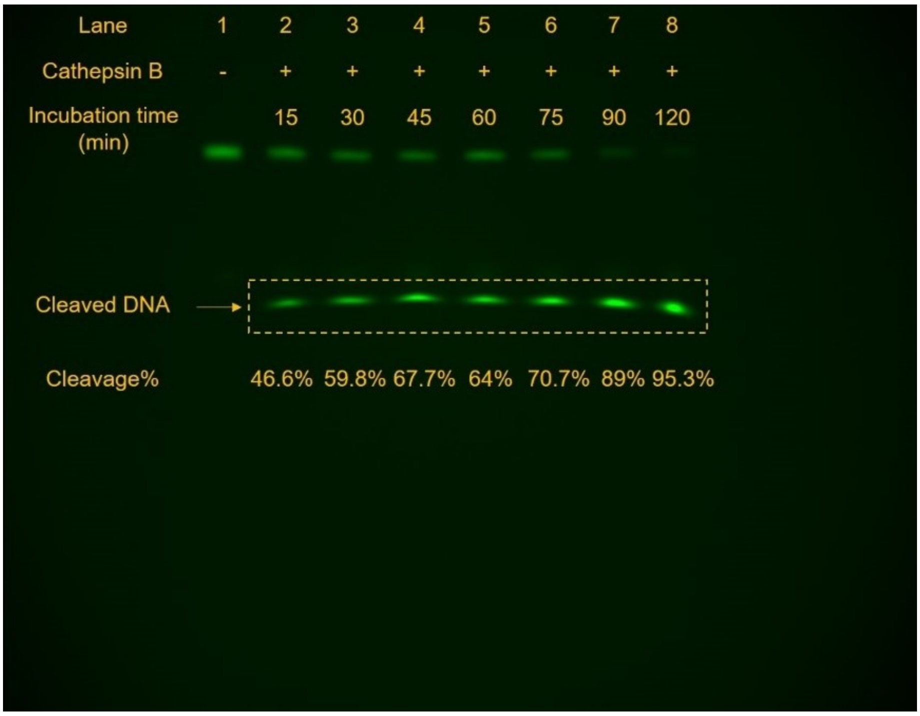

Figure S11. 12% Native PAGE gel analysis of cathepsin B-mediated enzymatic cleavage kinetics of FAM-ODN4UV (5' FAM-TTTTTTTTTT-Val-Ala-TTTTTTTTTTTT 3'). 2 μM FAM-ODN4UV was incubated with 0.2 U/mL cathepsin B in buffer A at 37 °C for various incubation intervals. After incubation, the mixture was subjected to electrophoresis gel analysis. The gel was run for one hour at room temperature and the power was set at 20 W. 2 μM FAM-ODN4UV was completely cleaved by 0.2 U/mL cathepsin B within two hours of incubation in buffer A.

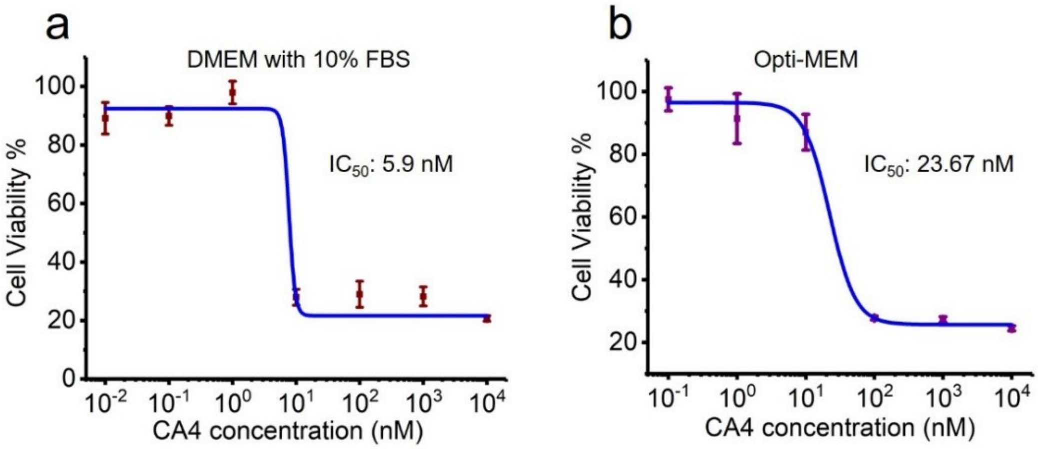

Figure S12. Cell viability of HCT116 cells after incubation with CA4 in DMEM cell culture medium supplemented with10% FBS (**a**) and Opti-MEM cell culture medium (**b**) for 48 hours.

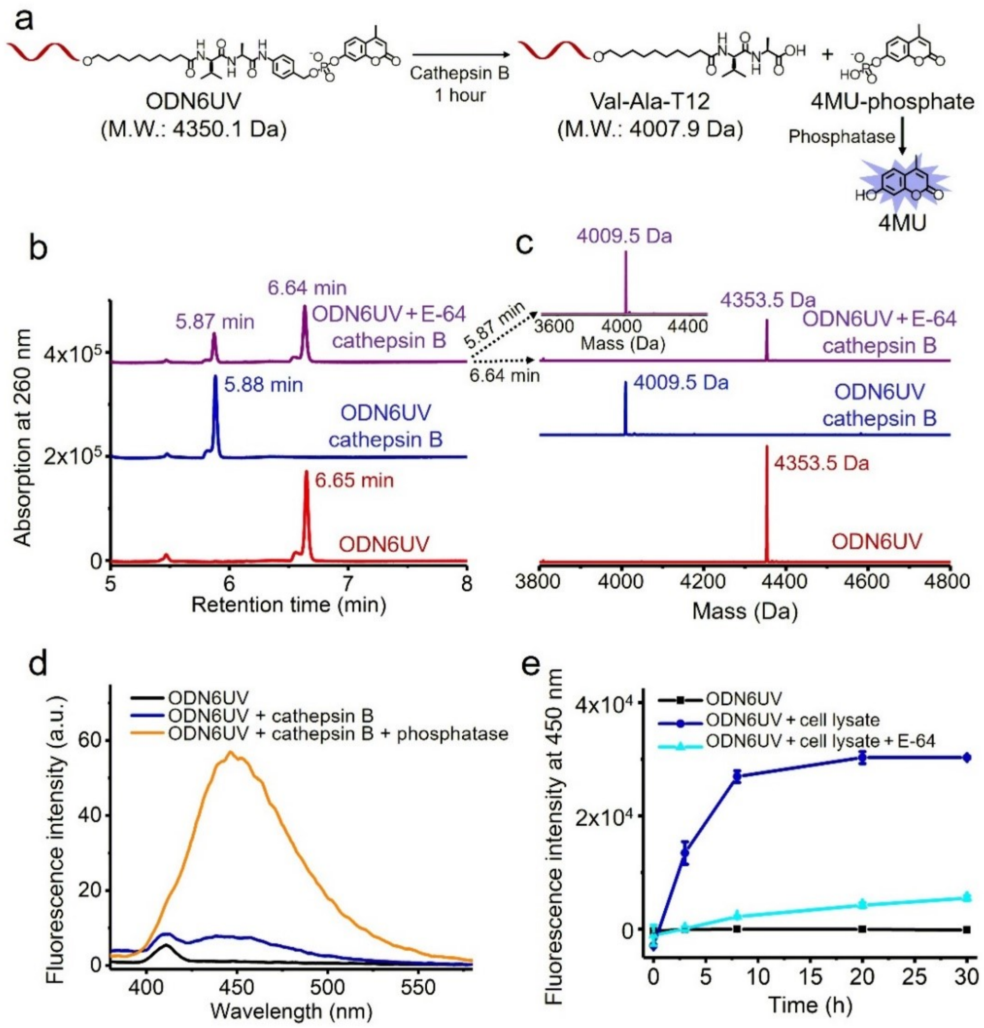

Figure S13. Cathepsin B-mediated cleavage of ODN6UV. (**a**) Cathepsin B-mediated cleavage reaction and conversion of 4MU-phosphate to 4MU by phosphatase. (**b**) HPLC traces of 10 μM ODN6UV after incubation without cathepsin B (red line), with 0.2 U/mL cathepsin B (blue line) or with 0.2 U/mL cathepsin B and 1 μM E-64 (purple line) in buffer A at 37 °C for 1 hour. (**c**) Mass spectra of 10 μM ODN6UV after incubation without cathepsin B (red line), with 0.2 U/mL cathepsin B (blue line) or with 0.2 U/mL cathepsin B and 1 μM E-64 (purple line) in buffer A at 37 °C for 1 hour. After incubation in buffer A at 37 °C for 1 hour, Val-Ala dipeptide in ODN6UV was completely cleaved by cathepsin B. (**d**) Fluorescence spectra of 2 μM ODN6UV before and after treatment with 0.2 U/mL cathepsin B and 0.2 U/mL acid phosphatase in buffer A at 37 °C for 1 hour. Black line: ODN6UV. Blue line: ODN6UV incubated with cathepsin B. Orange line: ODN6UV incubated with cathepsin B and then incubated with acid phosphatase. Excitation wavelength: 360 nm, emission wavelength: 380 to 650 nm. (**e**) Fluorescence intensity of 5 μM ODN6UV at 450 nm after incubation with 100 μg/mL HCT116 cell lysate in buffer A with (cyan line) or without (blue line) 10 μM E-64 at 37 °C for various times. The fluorescence intensity was measured on a microplate reader (INFINITE M1000 PRO). Excitation wavelength: 360 nm, emission wavelength: 450 nm, both slit widths were set as 20 nm.

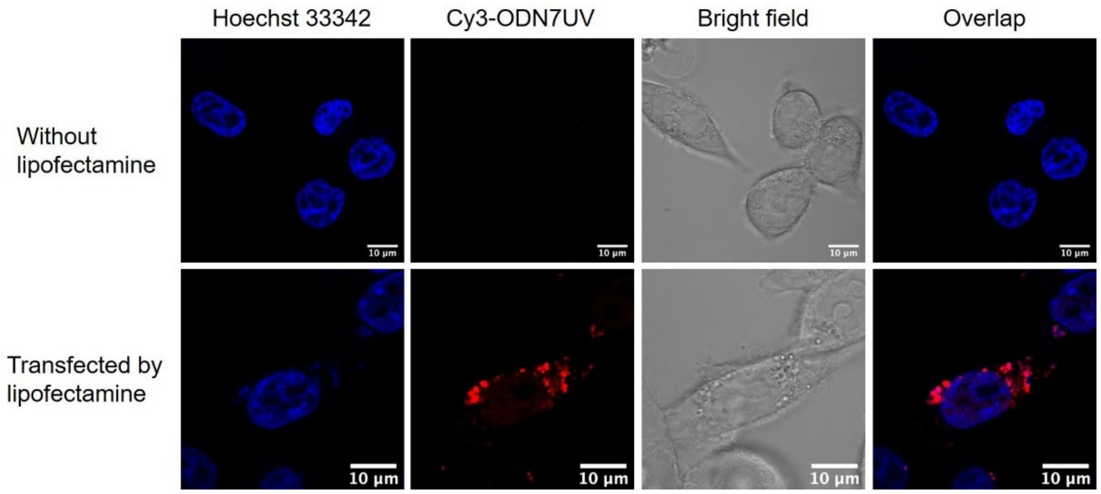

Figure S14. Confocal fluorescence microscopy imaging of HCT116 cells treated with Cy3-ODN7UV (1 μM) with or without transfection by lipofectamine in Opti-MEM culture medium at 37 °C for 6 hours. Cy3-ODN7UV: 5’ CA4-Val-Ala-TTTTTTTTTTTT-Cy3 3’. Scale bar: 10 μm.

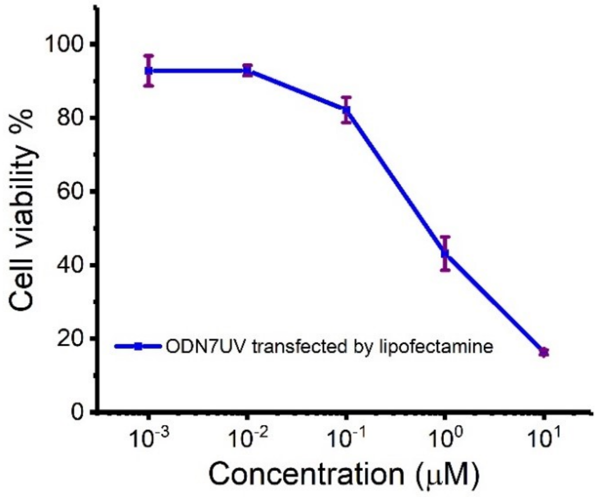

Figure S15. Cell viability of HCT116 cells after incubation with lipofectamine-transfected ODN7UV in Opti-MEM culture medium at 37 °C for 48 hours. The IC_{50} value of lipofectamine/ODN7UV complex to HCT116 cells after 48 hours of incubation in Opti-MEM culture medium is 0.79±0.4 μM.

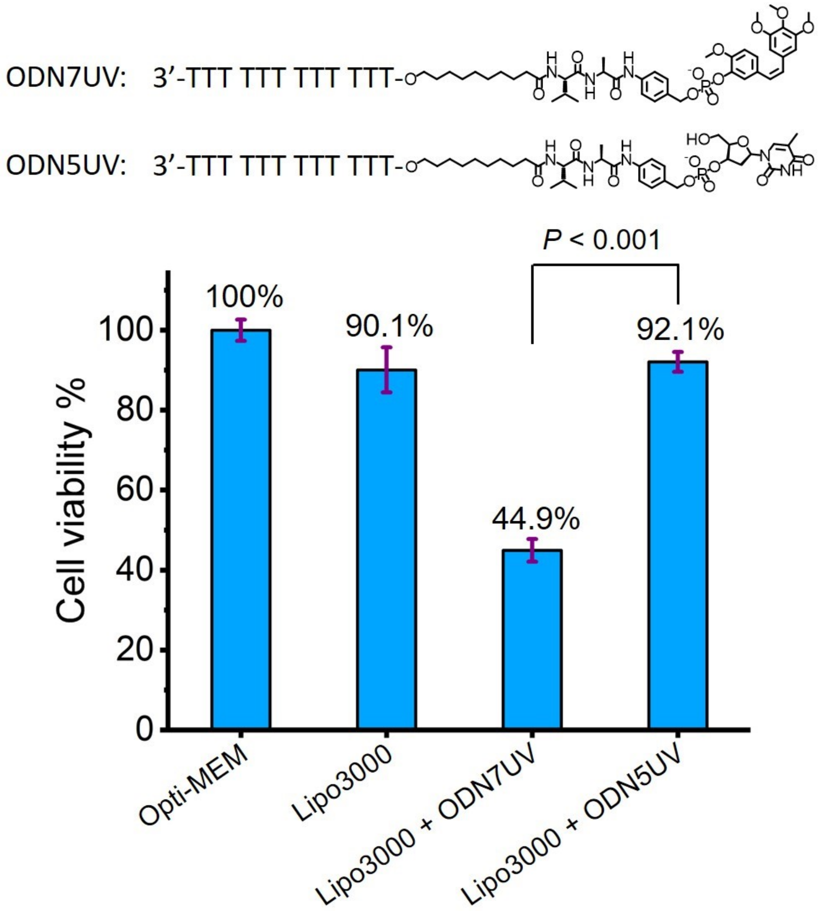

Figure S16. Cell viability of HCT116 cells after incubation with lipofectamine-transfected ODN7UV (1 μM) or lipofectamine-transfected ODN5UV (1 μM) in Opti-MEM cell culture medium at 37 °C for 48 hours.

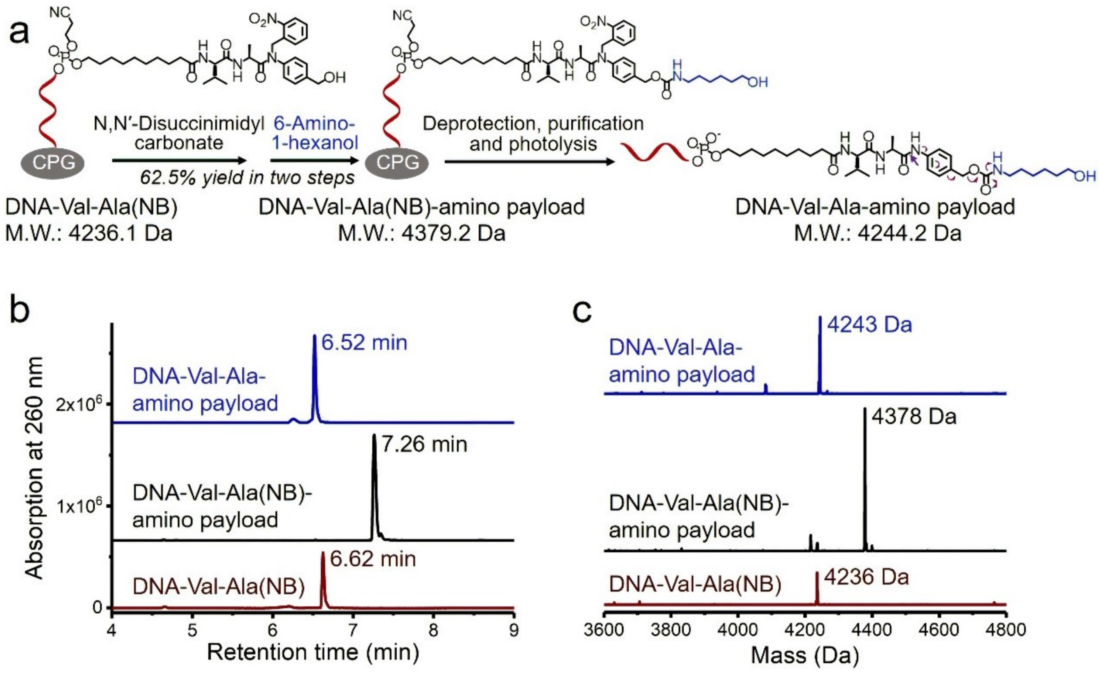

Figure S17. Solid-phase conjugation of small molecules containing an amino group with Val-Ala(NB) dipeptide linker through a carbamate linkage. (**a**) Schematic of activation and conjugation with 6-amino-1-hexanol of DNA-Val-Ala(NB) on resins, and the deprotection, purification and photolysis of DNA-Val-Ala(NB)-amino payload. (**b**) HPLC traces of DNA-Val-Ala(NB) (purple line), DNA-Val-Ala(NB)-amino payload (black line) and DNA-Val-Ala-amino payload (blue line). (**c**) Mass spectra of DNA-Val-Ala(NB) (purple line), DNA-Val-Ala(NB)-amino payload (black line) and DNA-Val-Ala-amino payload (blue line).

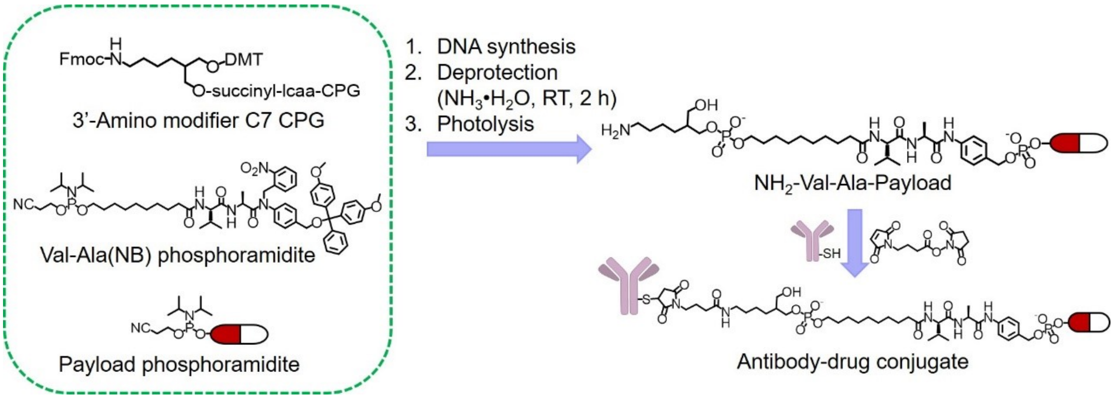

Figure S18. Automated synthesis of NH_2-Val-Ala-Payload conjugate through DNA solid-phase synthesis using of 3‘-amino CPG and Val-Ala(NB) and payload phosphoramidites. The NH_2-Val-Ala-Payload and 4-maleimidobutyric acid N-hydroxysuccinimide ester could enable the facile conjugation with antibodies via a Miachel addition reaction between thiol and maleimide groups.

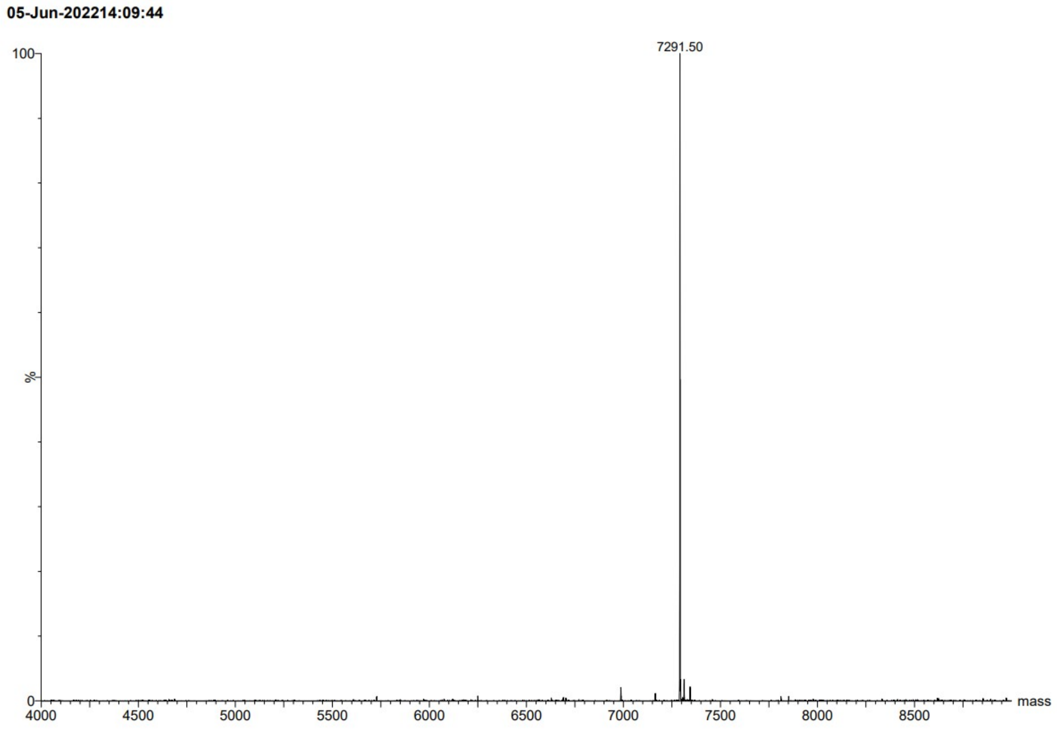

Figure S19. Mass spectrum of ODN4. The calculated molecular weight is 7290 Da, and observed molecular weight is 7291.5 Da.

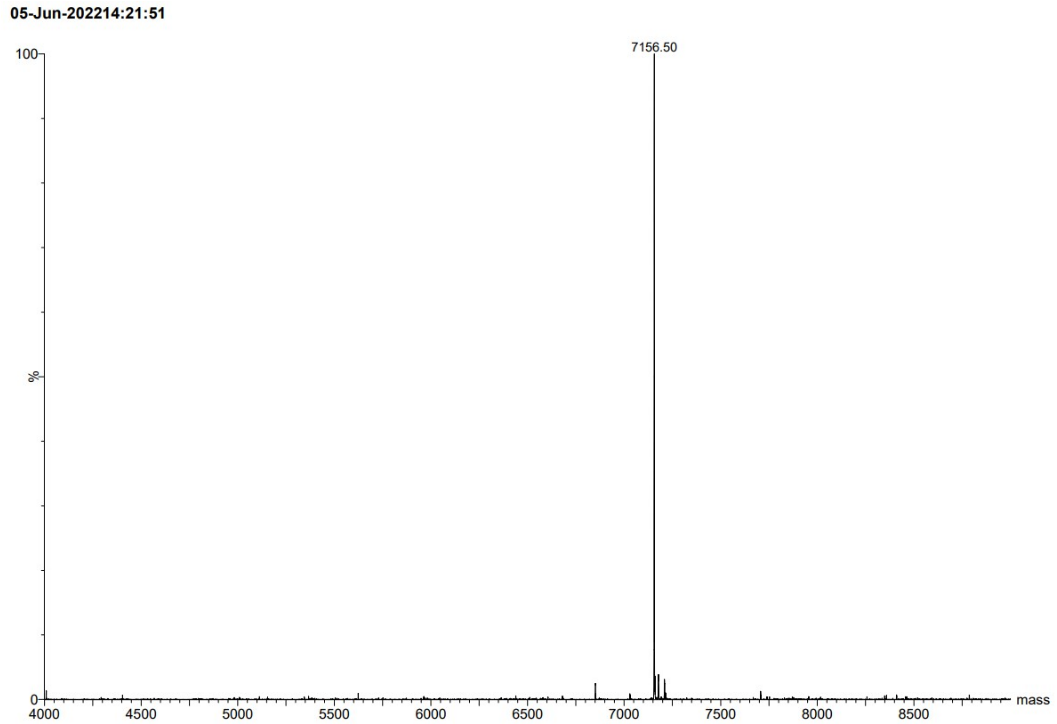

Figure S20. Mass spectrum of ODN4UV. The calculated molecular weight is 7154.9 Da, and observed molecular weight is 7156.5 Da.

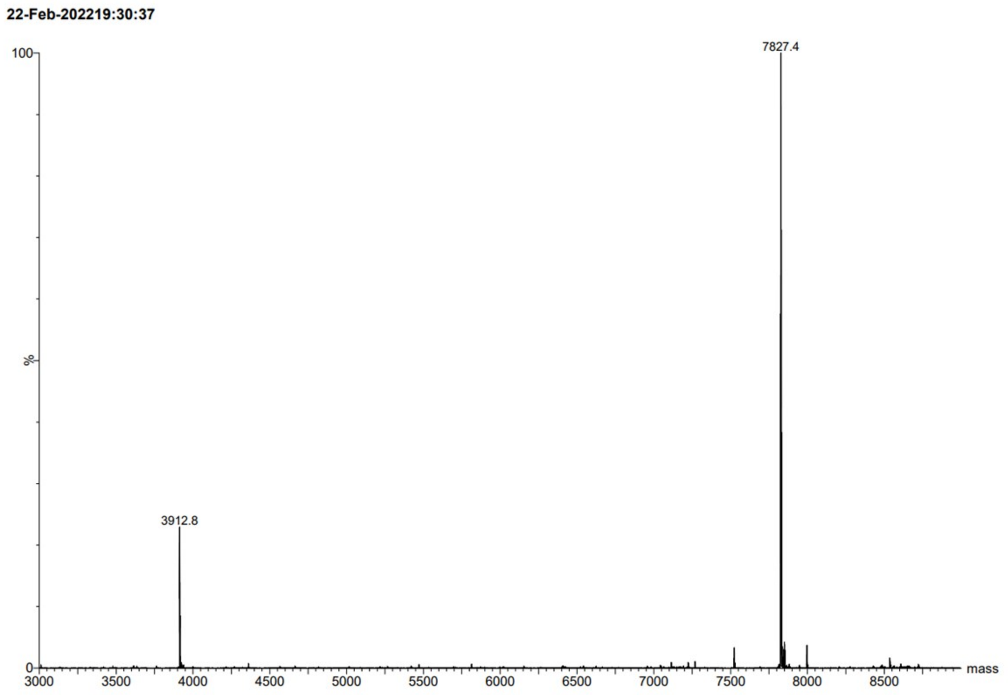

Figure S21. Mass spectrum of FAM-ODN4. The calculated molecular weight is 7826.7 Da, and observed molecular weight is 7827.4 Da. The DNA peak of 3912.8 Da is due to the half molecular weight of FAM-ODN4.

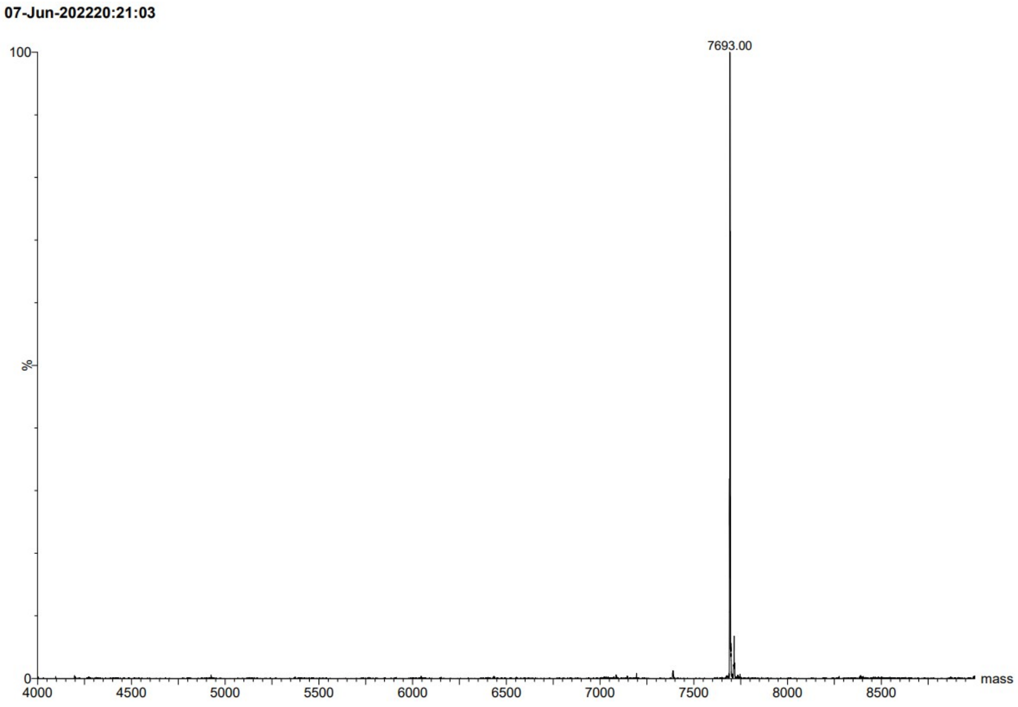

Figure S22. Mass spectrum of FAM-ODN4UV. The calculated molecular weight is 7692.5 Da, and observed molecular weight is 7693 Da.

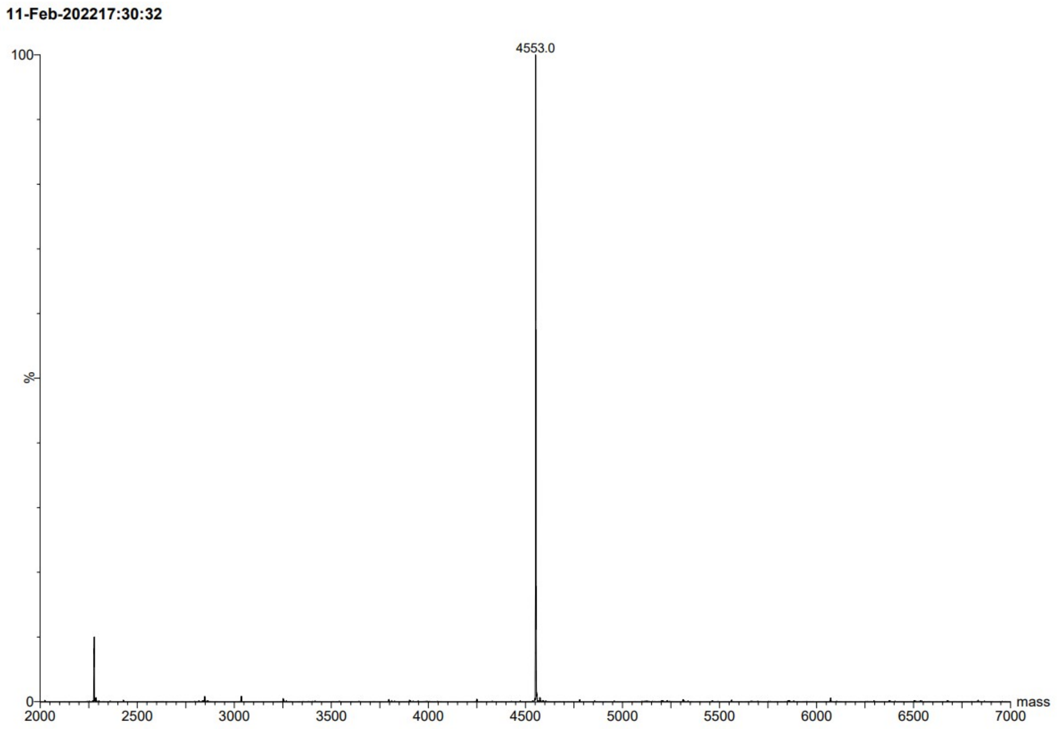

Figure S23. Mass spectrum of ODN5. The calculated molecular weight is 4552.3 Da, and observed molecular weight is 4553 Da. The DNA peak of 2276.5 Da is due to the half molecular weight of ODN5.

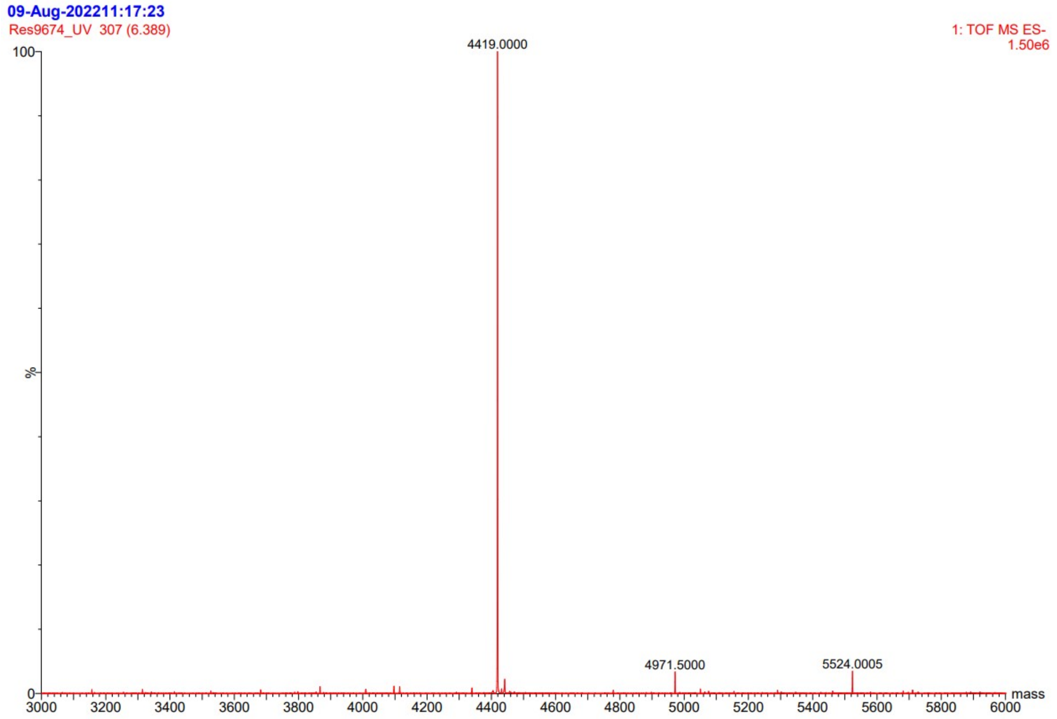

Figure S24. Mass spectrum of ODN5UV. The calculated molecular weight is 4417.2 Da, and observed molecular weight is 4419 Da.

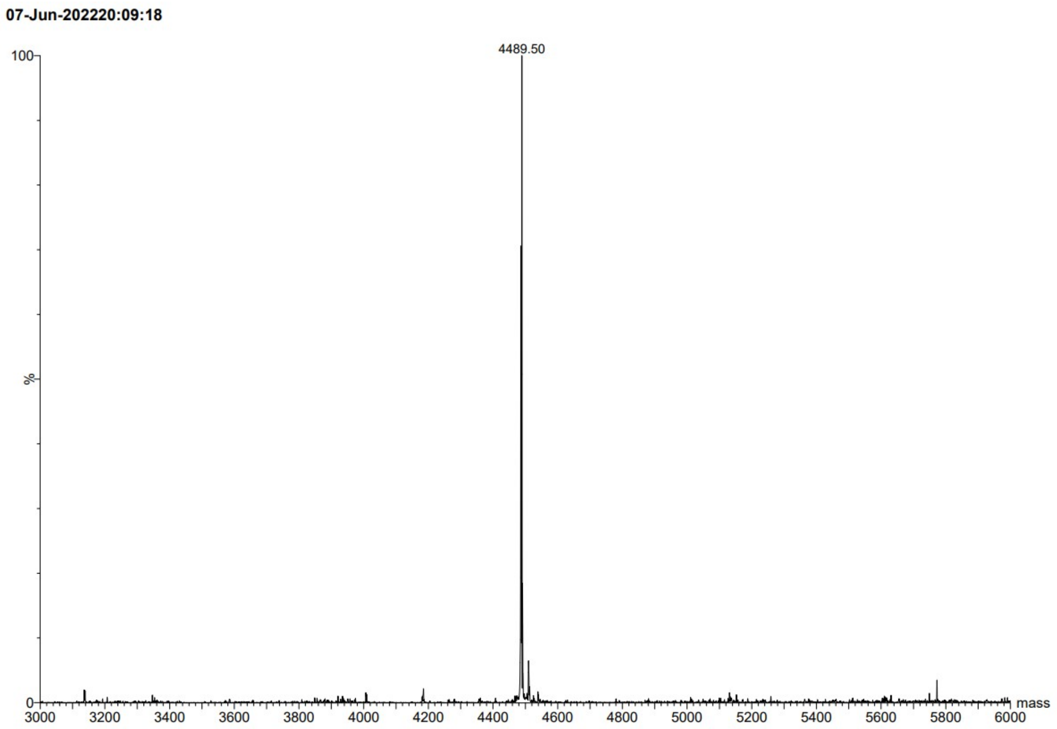

Figure S25. Mass spectrum of ODN6. The calculated molecular weight is 4486.2 Da, and observed molecular weight is 4489.5 Da.

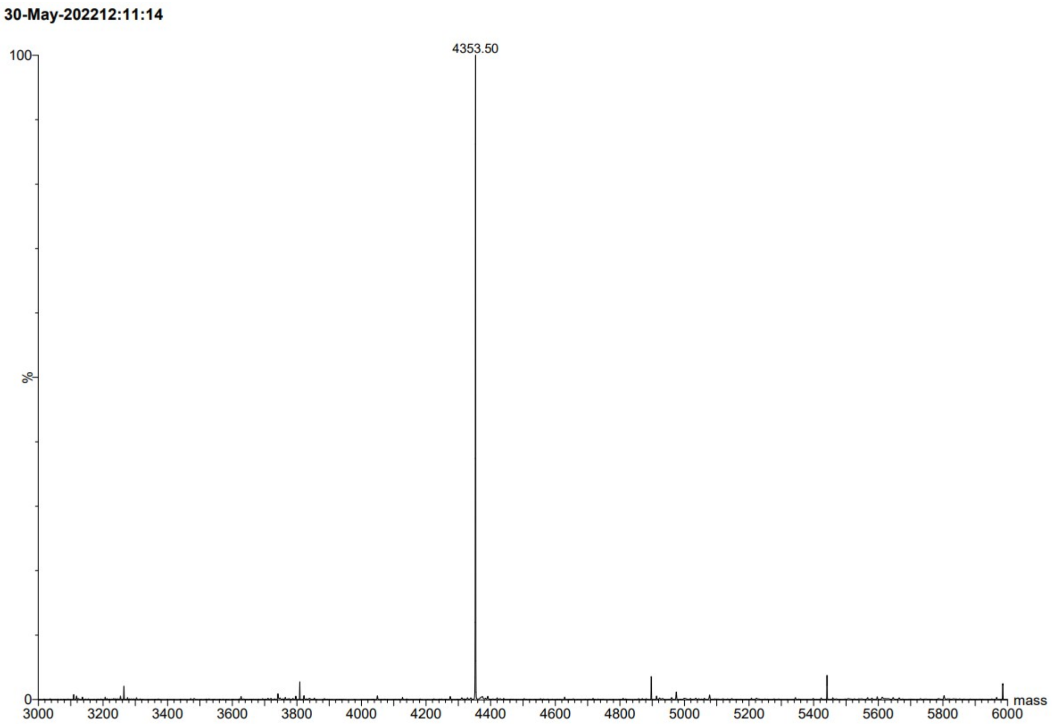

Figure S26. Mass spectrum of ODN6UV. Calculated molecular weight is 4351.1 Da, and observed molecular weight is 4353.5 Da.

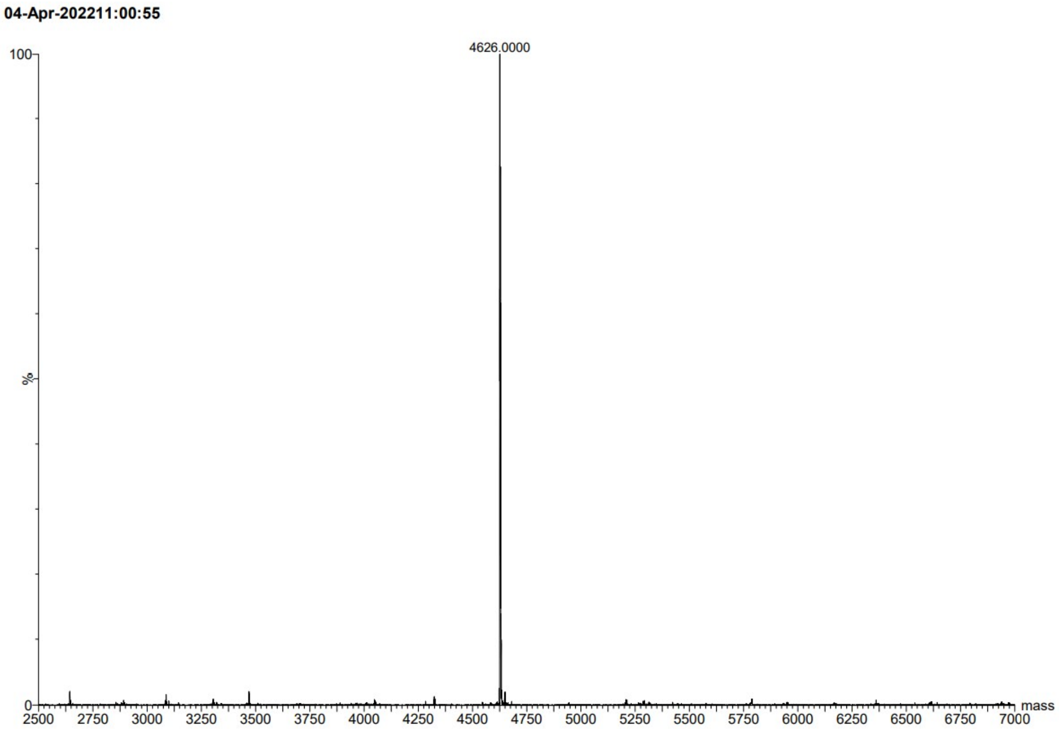

Figure S27. Mass spectrum of ODN7. The calculated molecular weight is 4625.4 Da, and observed molecular weight is 4626 Da.

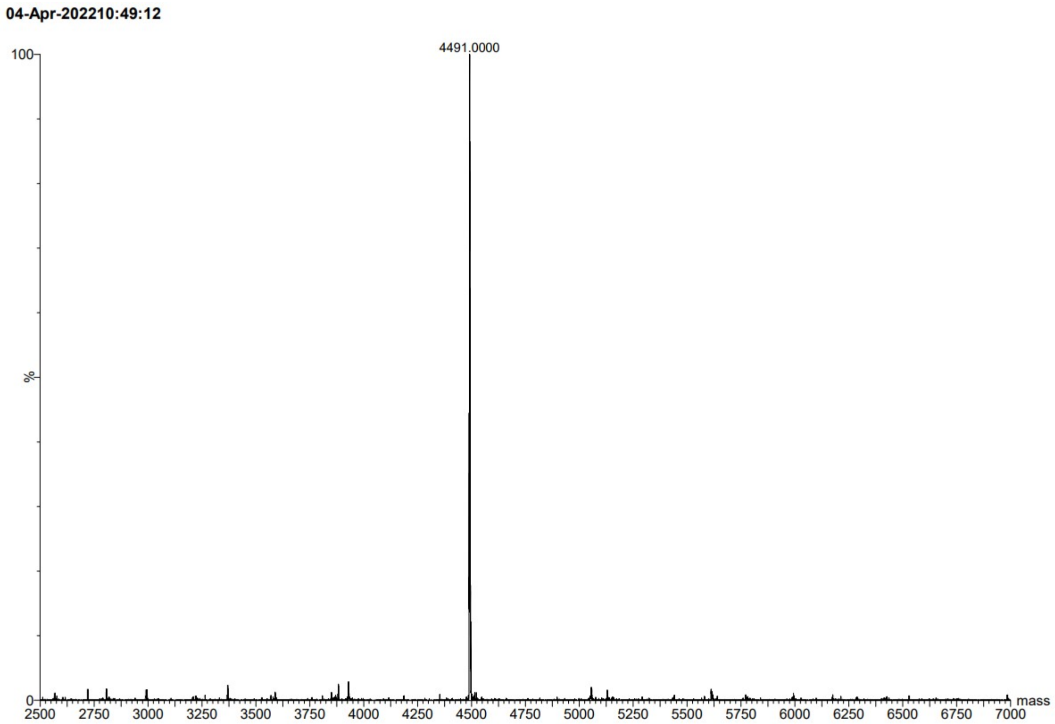

Figure S28. Mass spectrum of ODN7UV. The calculated molecular weight is 4490.3 Da, and observed molecular weight is 4491 Da.

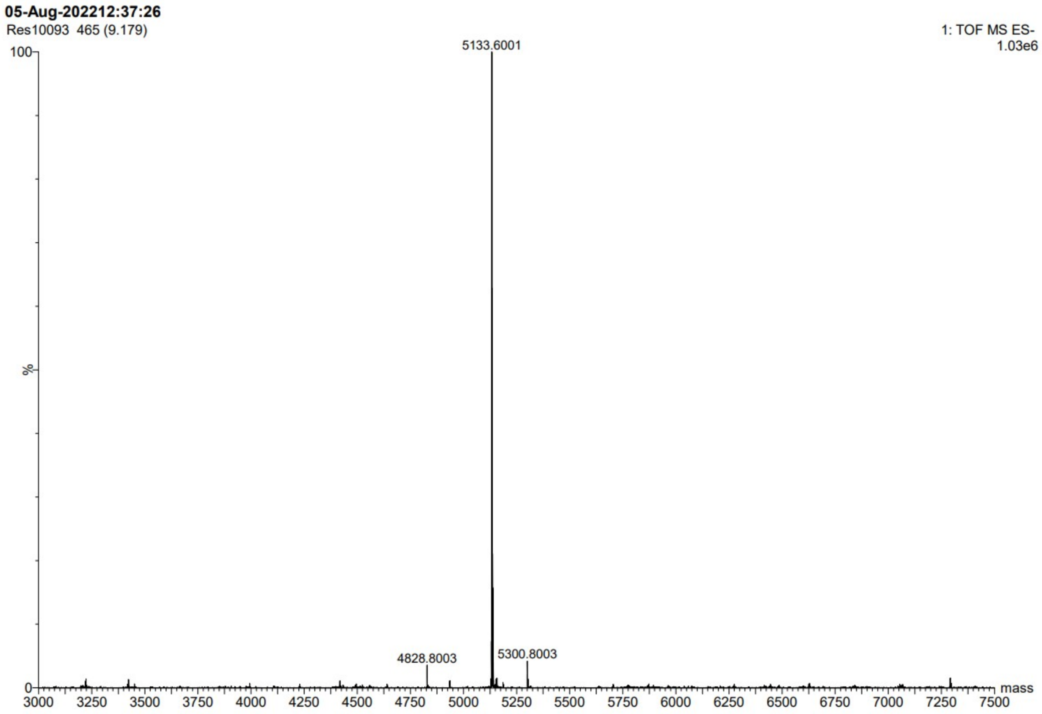

Figure S29. Mass spectrum of Cy3-ODN7. The calculated molecular weight is 5132 Da, and observed molecular weight is 5133.6 Da.

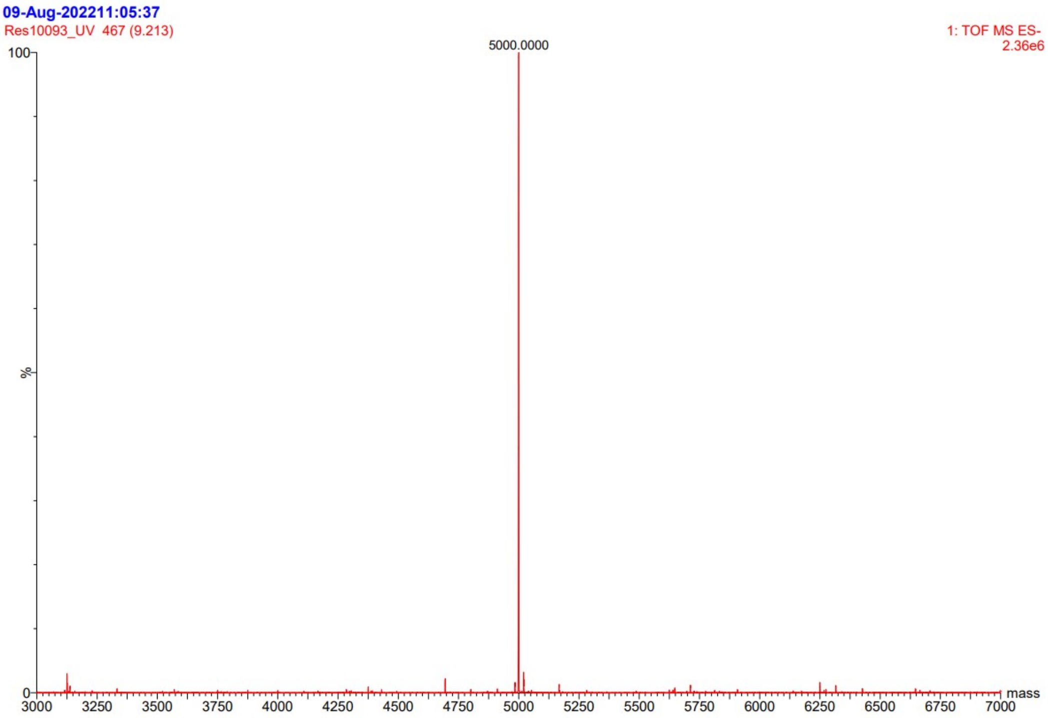

Figure S30. Mass spectrum of Cy3-ODN7UV. The calculated molecular weight is 4998.9 Da, and observed molecular weight is 5000 Da.

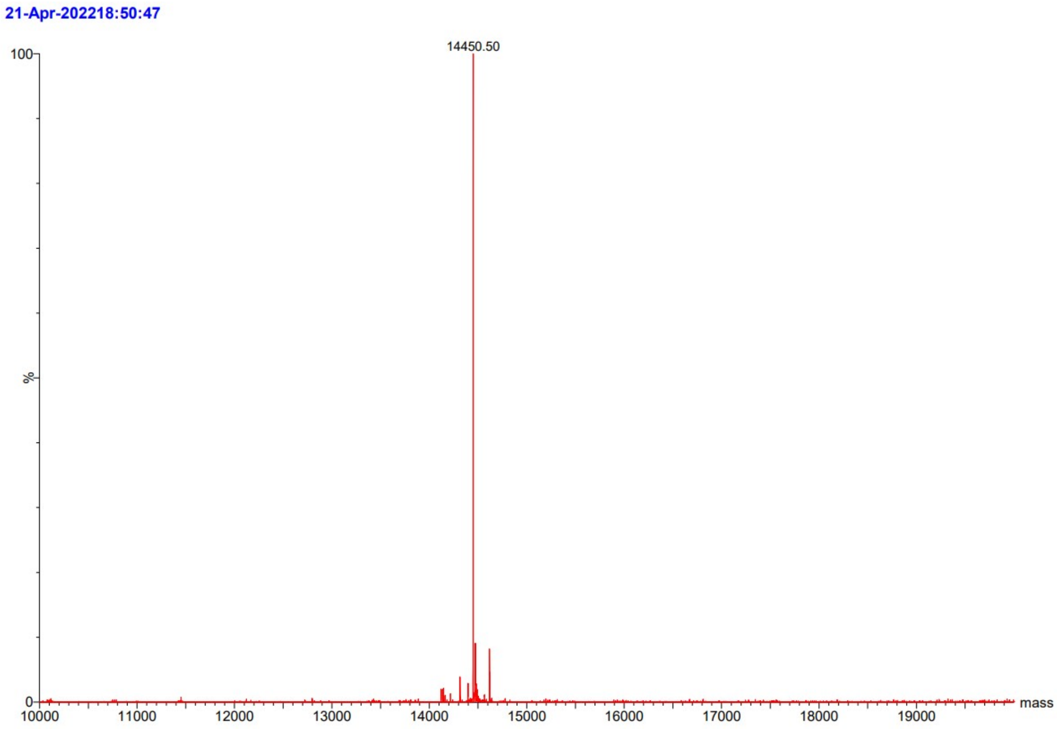

Figure S31. Mass spectrum of Sgc8-CA4 (5‘ CA4-Val-Ala-TTT ATC TAA CTG CTG CGC CGC CGG GAA AAT ACT GTA CGG TTA GA 3‘). The calculated molecular weight is 14448.7 Da, and observed molecular weight is 14450.5 Da.

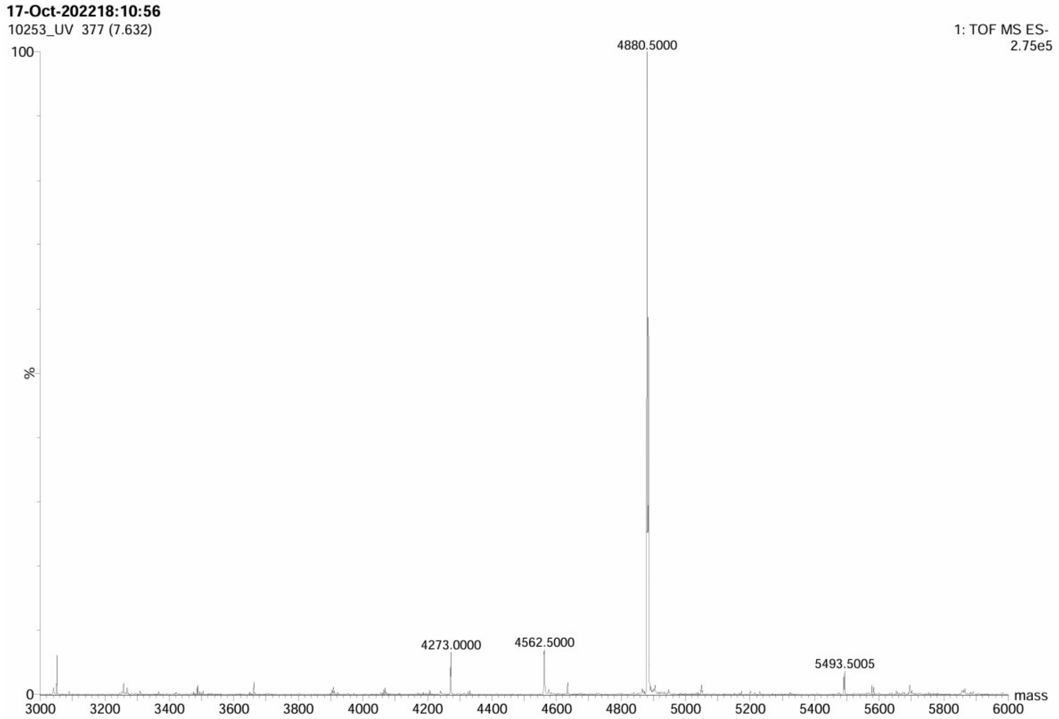

Figure S32. Mass spectrum of GalNAc-CA4 (5‘ CA4-Val-Ala-$U_{OMe}U_{OMe}U_{OMe}U_{OMe}U_{OMe}U_{OMe}$-GalNAc-GalNAc-GalNAc-T 3‘). The calculated molecular weight is 4881 Da, and observed molecular weight is 4880.5 Da.

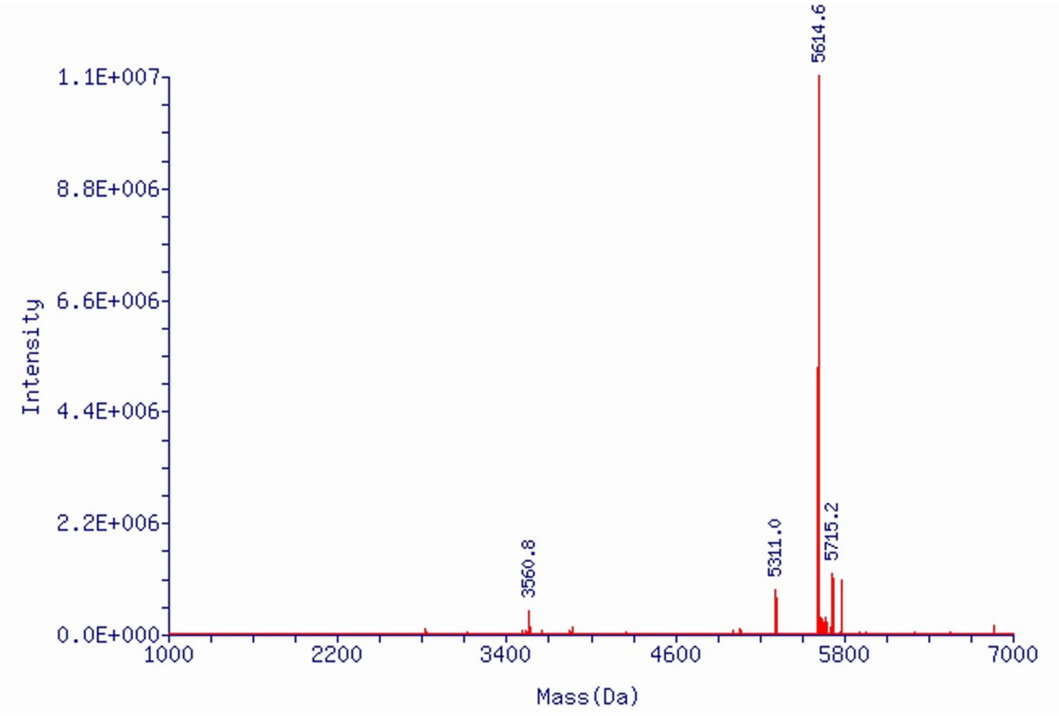

Figure S33. Mass spectrum of NH_2-DNA-CA4 (5‘ CA4-Val-Ala-TTT TTT TTT TTT TTT-NH_2 3‘). The calculated molecular weight is 5613.1 Da, and observed molecular weight is 5614.6 Da.

4. Synthesis of phosphoramidites

4.1 Synthesis of benzyl phosphoramidite

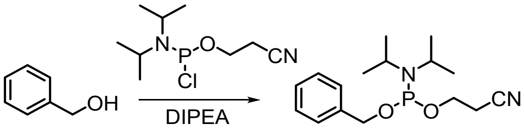

Figure S34. Synthesis of benzyl phosphoramidite.

Benzyl alcohol (0.2 g, 1.85 mmol) was dissolved in 20 mL anhydrous dichloromethane under argon gas protection, and 0.96 mL N,N-diisopropylethylamine (DIPEA) (0.71 g, 5.54 mmol) was added. The mixture was cooled down under ice bath. Then, 0.61 mL 2-cyanoethyl N,N-diisopropylchlorophosphoramidite (0.65 g, 2.74 mmol) was added dropwise. The reaction was monitored by thin-layer chromatography. When the reaction was completed, the mixture was diluted with 50 mL dichloromethane and washed successively with saturated $NaHCO_3$ and brine. The organic layer was collected and dried by anhydrous sodium sulfate. After purification by silica gel column chromatography (elution solvents: ethyl acetate/petroleum ether 1:3 to 1:1 v/v and 1% triethylamine was added), 0.18 g benzyl phosphoramidite was obtained as colorless oily. ^{1}H NMR (400 MHz, DMSO-*d*6) δ 7.40 – 7.32 (m, 4H), 7.29 (tt, *J* = 5.4, 4.3 Hz, 1H), 4.69 (qd, *J* = 12.7, 8.6 Hz, 2H), 3.87 – 3.69 (m, 2H), 3.69 – 3.55 (m, 2H), 2.78 (t, *J* = 5.9 Hz, 2H), 1.16 (dd, *J* = 8.4, 6.8 Hz, 12H). ^{31}P NMR (162 MHz, DMSO-*d*6) δ 147.31.

4.2 Synthesis of PAB phosphoramidite

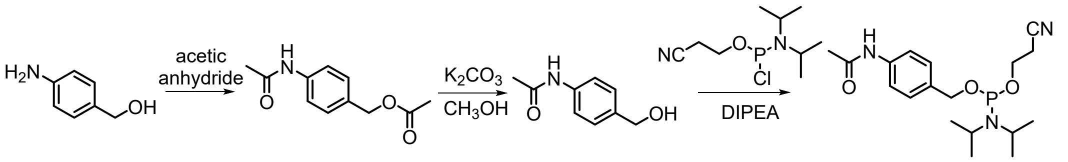

4-Aminobenzyl alcohol | 4-Acetamidobenzyl acetate | *N*-(4-(hydroxymethyl)phenyl) acetamide | PAB phosphoramidite

Figure S35. Synthesis of PAB phosphoramidite.

Synthesis of 4-acetamidobenzyl acetate

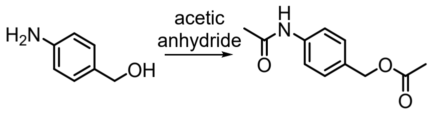

4-Aminobenzyl alcohol | 4-Acetamidobenzyl acetate

4-Aminobenzyl alcohol (0.51 g, 4.14 mmol) was dissolved in 30 mL dichloromethane. Triethylamine (1.44 mL, 10.3 mmol) and 1.69 g acetic anhydride (16.5 mmol) were added. The reaction was stirred at room temperature overnight. Then, the mixture was diluted with 100 mL dichloromethane and washed successively by saturated $NaHCO_3$ and brine. The organic layer was collected and dried by anhydrous Na_2SO_4. After removing organic solvent under reduced pressure, the residue was treated with 50 mL hexane, and the white precipitates were collected and dried under high vacuum. After drying, 0.73 g 4-acetamidobenzyl acetate was obtained as white solid (85% yield). ^{1}H NMR (400 MHz, $CDCl_3$) δ 7.69 (s, 1H), 7.44 (d, *J* = 8.4 Hz, 2H), 7.22 (d, *J* = 8.4 Hz, 2H), 4.98 (s, 2H), 2.09 (s, 3H), 2.01 (s, 3H). ^{13}C NMR (101 MHz, $CDCl_3$) δ 170.99, 168.63, 138.06, 131.71, 129.17, 119.92, 65.95, 24.53, 21.05.

Synthesis of N-(4-(hydroxymethyl)phenyl)acetamide

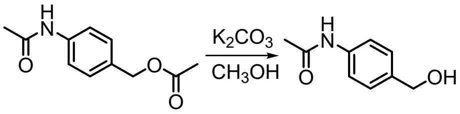

4-Acetamidobenzyl acetate | *N*-(4-(hydroxymethyl)phenyl) acetamide

4-Acetamidobenzyl acetate (0.25 g, 1.2 mmol) was dissolved in 10 mL methanol, and 0.5 g K_2CO_3 (3.61 mmol) was added. The mixture was stirred overnight. Then, the solvent was removed under reduced pressure, and the residue was dissolved in 50 mL dichloromethane and washed with saturated brine. The organic layer was collected and dried with anhydrous Na_2SO_4. After drying under high vacuum, 114 mg N-(4-(hydroxymethyl)phenyl)acetamide was obtained (57.5% yield). ^{1}H NMR (400 MHz, DMSO-*d6*) δ 9.84 (s, 1H), 7.48 (d, *J* = 8.5 Hz, 2H), 7.18 (d, *J* = 8.5 Hz, 2H), 5.04 (s, 1H), 4.38 (s, 2H), 1.99 (s, 3H). ^{13}C NMR (101 MHz, DMSO-*d6*) δ 168.11, 137.95, 137.07, 126.90, 118.70, 62.63, 23.96.

Synthesis of PAB phosphoramidite

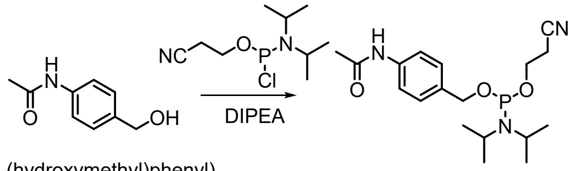

N-(4-(hydroxymethyl)phenyl)acetamide (105 mg, 0.63 mmol) was dissolved in 20 mL anhydrous dichloromethane under argon gas protection, and 0.32 mL DIPEA (0.24 g, 1.9 mmol) was added. The mixture was cooled down under ice bath. Then, 0.25 mL 2-cyanoethyl N,N-diisopropylchlorophosphoramidite (0.23 g, 0.97 mmol) was added dropwise. The reaction was monitored by thin-layer chromatography. When the reaction was completed, the mixture was diluted with 50 mL dichloromethane and washed successively with saturated $NaHCO_3$ and brine. The organic layer was collected and dried by anhydrous Na_2SO_4. After purification by silica gel column chromatography (elution solvents: ethyl acetate and 1% triethylamine was added), 80 mg PAB phosphoramidite was obtained as colorless oily. ^{1}H NMR (400 MHz, DMSO-*d*6) δ 9.94 (s, 1H), 7.55 (d, *J* = 8.5 Hz, 2H), 7.25 (d, *J* = 8.5 Hz, 2H), 4.61 (ddd, *J* = 27.9, 12.4, 8.7 Hz, 2H), 3.81 – 3.66 (m, 2H), 3.66 – 3.53 (m, 2H), 2.77 (t, *J* = 5.9 Hz, 2H), 2.03 (s, 3H), 1.14 (dd, *J* = 8.7, 6.8 Hz, 12H). ^{31}P NMR (162 MHz, DMSO-*d*6) δ 147.17.

4.3 Synthesis of N-methylated PAB (mPAB) phosphoramidite

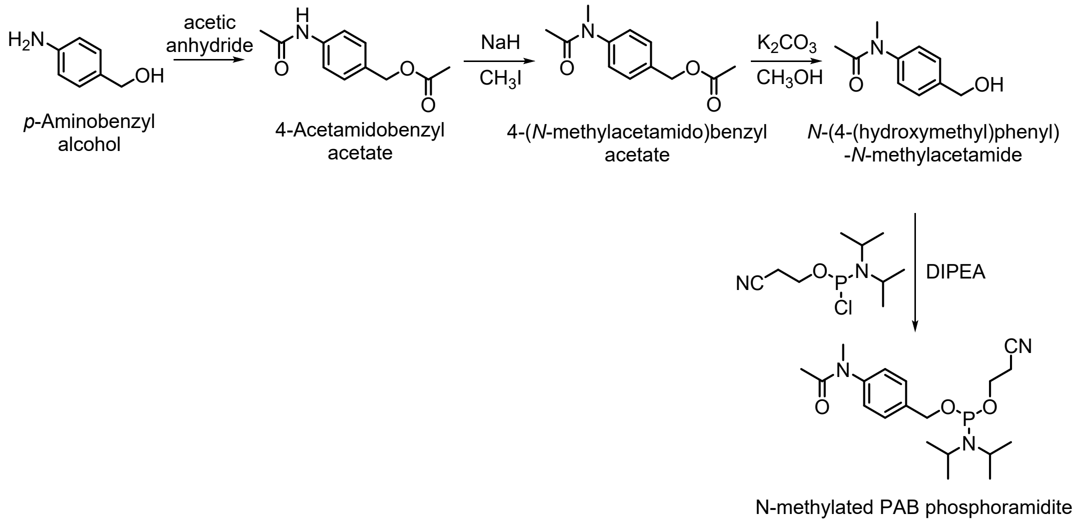

Figure S36. Synthesis of N-methylated PAB (mPAB) phosphoramidite.

Synthesis of 4-(N-methylacetamido)benzyl acetate

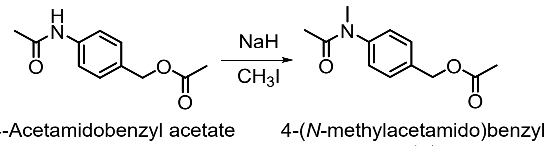

4-acetamidobenzyl acetate (0.442 g, 2.13 mmol) was dissolved in 10 mL dry tetrahydrofuran under argon gas protection. The mixture was cooled down under ice bath, and 84 mg NaH (60% dispersion in mineral oil) was added. The reaction was warmed to room temperature. When the hydrogen evolution ceased, 0.12 mL CH_3I was added, and the mixture was stirred for 30 minutes. Then, the mixture was poured into 40 mL saturated ammonium chloride and washed with ethyl acetate twice. The organic layer was collected and dried by anhydrous Na_2SO_4. After purification by silica gel column chromatography (elution solvent: ethyl acetate), 0.31 g 4-(N-methylacetamido)benzyl acetate was obtained (65.7% yield). ^{1}H NMR (400 MHz, $CDCl_3$) δ 7.34 (d, *J* = 8.3 Hz, 2H), 7.12 (d, *J* = 8.2 Hz, 2H), 5.05 (s, 2H), 3.19 (s, 3H), 2.06 (s, 3H), 1.81 (s, 3H). ^{13}C NMR (101 MHz, $CDCl_3$) δ 170.75, 170.45, 144.47, 135.61, 129.50, 127.24, 65.48, 37.13, 22.44, 20.97.

Synthesis of N-(4-(hydroxymethyl)phenyl)-N-methylacetamide

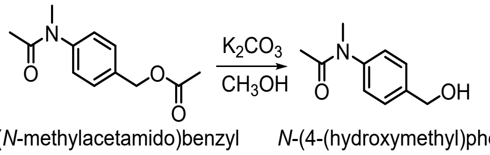

4-(*N*-methylacetamido)benzyl acetate   *N*-(4-(hydroxymethyl)phenyl)-*N*-methylacetamide

4-(N-methylacetamido)benzyl acetate (0.29 g, 1.31 mmol) was dissolved in 20 mL methanol, 0.54 g K_2CO_3 (3.91 mmol) was added. The mixture was stirred overnight under room temperature. Then, the organic solvent was removed under reduced pressure, and the residue was dissolved in 100 mL dichloromethane and washed successively with saturated $NaHCO_3$ and brine. The organic layer was collected and dried by anhydrous Na_2SO_4. After purification by silica gel column chromatography (elution solvent: ethyl acetate), 185 mg of N-(4-(hydroxymethyl)phenyl)-N-methylacetamide was obtained (78.6% yield). ^{1}H NMR (400 MHz, $CDCl_3$) δ 7.36 (d, *J* = 8.2 Hz, 2H), 7.11 (d, *J* = 8.2 Hz, 2H), 4.67 (s, 2H), 3.18 (s, 3H), 2.28 (s, 1H), 1.80 (s, 3H). ^{13}C NMR (101 MHz, $CDCl_3$) δ 170.72, 143.77, 140.70, 128.19, 127.11, 64.49, 37.21, 22.37.

Synthesis of mPAB phosphoramidite

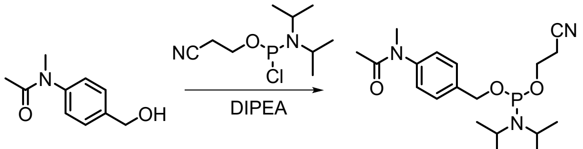

N-(4-(hydroxymethyl)phenyl)-*N*-methylacetamide   mPAB phosphoramidite

N-(4-(hydroxymethyl)phenyl)-N-methylacetamide (170 mg, 0.95 mmol) was dissolved in 20 mL anhydrous dichloromethane under argon gas protection, and 0.5 mL DIPEA (2.84 mmol) was added. The mixture was cooled down under ice bath. Then, 0.32 mL 2-cyanoethyl N,N-diisopropylchlorophosphoramidite (1.42 mmol) was added dropwise. The reaction was monitored by thin-layer chromatography. When the reaction was completed, the mixture was diluted with 50 mL dichloromethane and washed successively with saturated $NaHCO_3$ and

brine. The organic layer was collected and dried by anhydrous Na_2SO_4. After purification by silica gel column chromatography (elution solvents: ethyl acetate and 1% triethylamine was added), 150 mg mPAB phosphoramidite was obtained as colorless oily (42.1% yield). ^1H NMR (400 MHz, $CDCl_3$) δ 7.39 (d, J = 8.3 Hz, 2H), 7.16 (d, J = 8.3 Hz, 2H), 4.73 (ddd, J = 36.9, 12.8, 8.3 Hz, 2H), 3.97 – 3.78 (m, 2H), 3.75 – 3.60 (m, 2H), 3.25 (s, 3H), 2.65 (t, J = 6.4 Hz, 2H), 1.87 (s, 3H), 1.20 (dd, J = 8.4, 6.8 Hz, 12H). ^{31}P NMR (162 MHz, $CDCl_3$) δ 148.71. ^{13}C NMR (101 MHz, $CDCl_3$) δ 170.56, 144.04, 143.81, 128.24, 127.01, 117.57, 64.95, 64.77, 58.55, 58.36, 43.28, 43.16, 37.17, 24.70, 24.63, 24.57, 22.43, 20.45, 20.38.

4.4 Synthesis of Val-Ala(NB) phosphoramidite

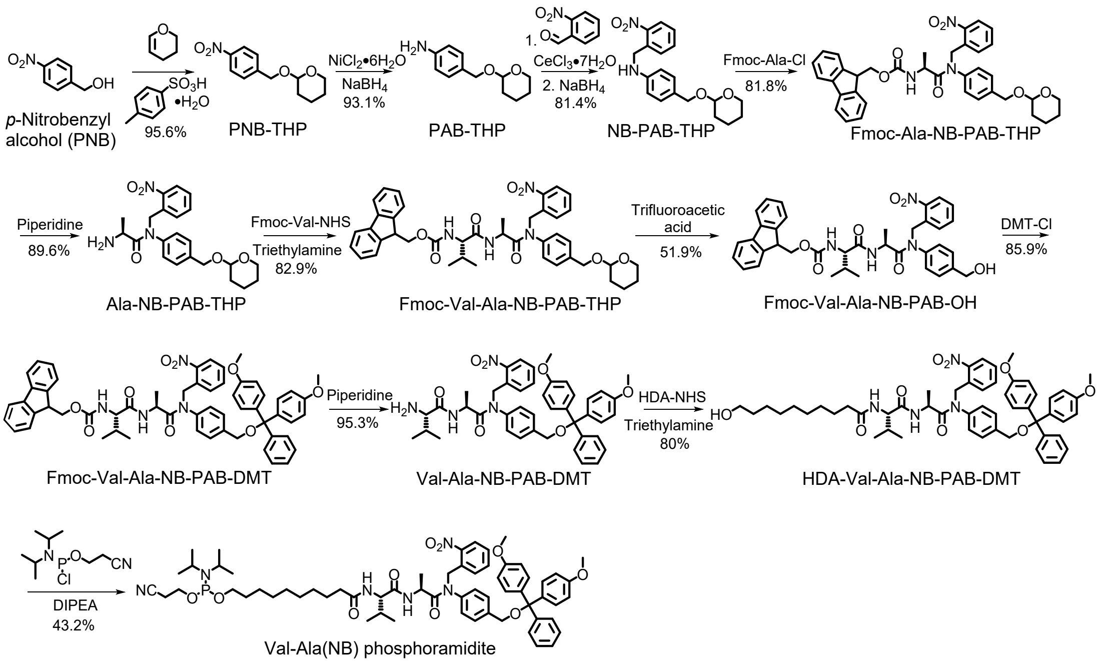

Figure S37. Synthesis of Val-Ala(NB) phosphoramidite.

Synthesis of PNB-THP

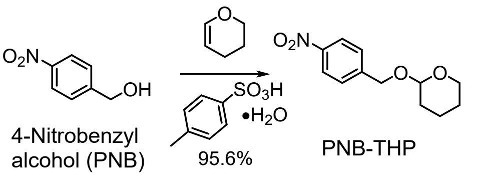

4-Nitrobenzyl alcohol (5.0 g, 32.6 mmol) was dissolved in 100 mL dichloromethane. 3,4-Dihydro-2H-pyran (4.12 g, 48.9 mmol) and 0.93 g p-toluenesulfonic acid monohydrate (4.9 mmol) were added. The mixture was stirred under room temperature for 1.5 hours. Then, the mixture was washed with saturated $NaHCO_3$. The organic layer was collected and dried over anhydrous Na_2SO_4. After purification by silica gel column chromatography (elution solvents: ethyl acetate/petroleum ether 1:5 to 1:2 v/v), 7.4 g PNB-THP was obtained as yellow oily (95.6% yield). ^1H NMR (400 MHz, $CDCl_3$) δ 8.20 (d, J = 8.8 Hz, 2H), 7.53 (d, J = 8.9 Hz, 2H), 4.88 (d,

J = 13.5 Hz, 1H), 4.73 (t, J = 3.5 Hz, 1H), 4.60 (d, J = 13.5 Hz, 1H), 3.94 – 3.82 (m, 1H), 3.62 – 3.52 (m, 1H), 1.90 – 1.75 (m, 2H), 1.69 – 1.50 (m, 4H). ^{13}C NMR (101 MHz, $CDCl_3$) δ 147.29, 146.12, 127.77, 123.60, 98.31, 67.64, 62.29, 30.46, 25.36, 19.27.

Synthesis of PAB-THP

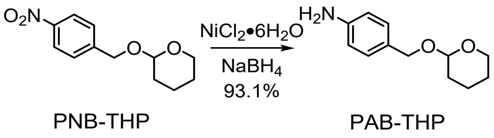

PNB-THP (1.18 g, 4.97 mmol) was dissolved in 20 mL CH_3CN in 150 mL flask. H_2O (2 mL) and 0.2 g nickel (II) chloride hexahydrate (0.84 mmol) were added. The mixture was stirred under room temperature for 5 minutes. Then, 0.63 g sodium borohydride (16.8 mmol) was added (*keeping the flask opened to balance gas pressure*), and the mixture was stirred for 30 minutes under room temperature. Then, the mixture was diluted with 30 mL H_2O and extracted with dichloromethane (100 mL). The organic layer was collected and dried by Na_2SO_4. The solvents were removed under reduced pressure, and the residue was dried under high vacuum. 0.96 g PAB-THP was obtained as oily (93.1% yield). ^{1}H NMR (400 MHz, $CDCl_3$) δ 7.17 (d, J = 8.4 Hz, 2H), 6.68 (d, J = 8.4 Hz, 2H), 4.67 (dd, J = 7.7, 3.9 Hz, 2H), 4.39 (d, J = 11.5 Hz, 1H), 3.92 (ddd, J = 11.5, 8.2, 3.4 Hz, 1H), 3.62 – 3.45 (m, 1H), 1.90 – 1.79 (m, 1H), 1.76 – 1.66 (m, 1H), 1.64 – 1.50 (m, 4H). ^{13}C NMR (101 MHz, $CDCl_3$) δ 145.48, 129.63, 128.43, 115.21, 97.35, 68.73, 62.17, 30.64, 25.53, 19.48.

Synthesis of NB-PAB-THP

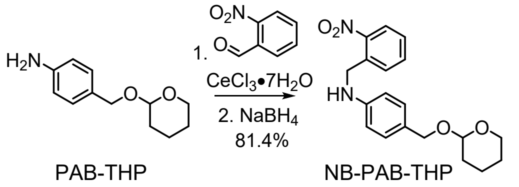

PAB-THP (0.95 g, 4.58 mmol) was dissolved in 25 mL methanol in 150 mL flask. 2-Nitrobenzaldehyde (0.76 g, 5.03 mmol) and 68 mg cerium (III) chloride heptahydrate (0.18 mmol) were added. The mixture was stirred at room temperature for 30 minutes. Then, 0.433 g sodium borohydride (11.45 mmol) was added (*keeping the flask opened to balance gas pressure*) and stirred for further 2 hours. The residue was diluted with 100 mL ethyl acetate and washed with saturated $NaHCO_3$. The organic layer was collected and dried over anhydrous Na_2SO_4. After purification by silica gel column chromatography (elution solvents: ethyl acetate/petroleum ether 1:4 to 1:3 v/v), 1.28 g NB-PAB-THP was obtained as orange oily (81.4% yield). ^{1}H NMR (400 MHz, $CDCl_3$) δ 7.98 (dd, J = 8.1, 1.3 Hz, 1H), 7.57 (d, J = 7.8 Hz, 1H), 7.47 (td, J = 7.6, 1.3 Hz, 1H), 7.37 – 7.16 (m, 2H), 7.08 (d, J = 8.5 Hz, 2H), 6.49 – 6.43 (m, 2H), 4.64 (s, 2H), 4.61 – 4.54 (m, 2H), 4.28 (d, J = 11.4 Hz, 1H), 3.84 (ddd, J = 11.4, 8.1, 3.1 Hz, 1H), 3.49 – 3.42 (m, 1H), 1.82 – 1.71 (m, 1H), 1.64 – 1.58 (m, 1H), 1.56 – 1.42 (m, 4H).

^{13}C NMR (101 MHz, $CDCl_3$) δ 147.02, 135.69, 133.77, 129.89, 129.81, 128.92, 128.09, 125.28, 121.33, 112.88, 97.56, 68.90, 62.27, 45.89, 30.73, 25.61, 19.58.

Synthesis of Fmoc-Ala-NB-PAB-THP

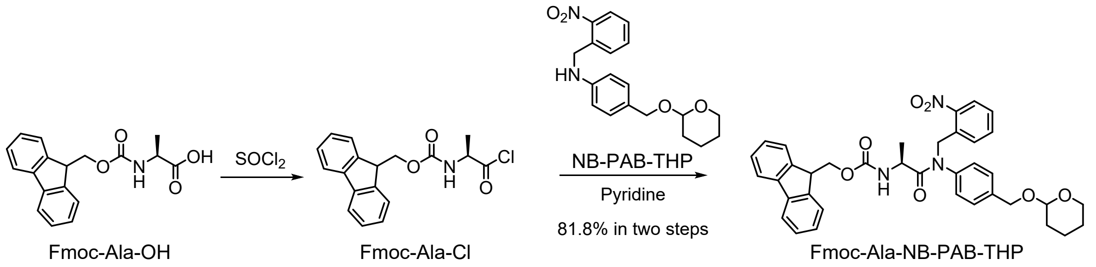

Fmoc-Ala-OH (1.5 g, 4.8 mmol) was dissolved in anhydrous dichloromethane (30 mL) in a two-neck flask under argon gas protection, and excess $SOCl_2$ (2.8 mL, 38.5 mmol) was added dropwise. The mixture was heated at reflux for 30 min. Then, the solvent was evaporated under reduced pressure, and the residue was purified by crystallization from anhydrous *n*-hexane. After drying, Fmoc-Ala-Cl was obtained as white solid and used for the next step without further purification.

NB-PAB-THP (3.7 g, 10.8 mmol) was dissolved in 30 mL anhydrous dichloromethane under argon gas protection, and dry pyridine (0.78 mL, 9.6 mmol) was added. Then, Fmoc-Ala-Cl (4.8 mmol) in dry dichloromethane was added dropwise. The mixture was stirred under room temperature for 24 hours. Then, the organic solvents were removed under reduced pressure, and the residue was purified by silica gel column chromatography (elution solvents: ethyl acetate/petroleum ether 1:4 to 1:1 v/v). After purification, 2.5 g Fmoc-Ala-NB-PAB-THP was obtained as foam solid (81.8% yield in two steps). ^{1}H NMR (400 MHz, CDCl3) δ 7.95 (d, *J* = 8.1 Hz, 1H), 7.76 (d, *J* = 7.5 Hz, 2H), 7.65 – 7.55 (m, 4H), 7.40 (t, *J* = 8.4 Hz, 5H), δ 7.31 (t, *J* = 7.5 Hz, 2H), 7.22 (d, *J* = 8.2 Hz, 2H), 5.55 (d, *J* = 8.0 Hz, 1H), 5.43 (dd, *J* = 16.5, 3.4 Hz, 1H), 5.17 (dd, *J* = 16.5, 2.9 Hz, 1H), 4.78 (d, *J* = 12.6 Hz, 1H), 4.71 (dd, *J* = 6.8, 3.4 Hz, 1H), 4.52 – 4.43 (m, 2H), 4.40 – 4.30 (m, 2H), 4.22 (t, *J* = 7.1 Hz, 1H), 3.88 (t, *J* = 10.4 Hz, 1H), 3.54 (d, *J* = 11.1 Hz, 1H), 1.85 (ddd, *J* = 13.7, 10.0, 4.0 Hz, 1H), 1.77 – 1.70 (m, 1H), 1.69 – 1.52 (m, 4H), 1.25 (d, *J* = 6.9 Hz, 3H). ^{13}C NMR (101 MHz, CDCl3) δ 173.83, 155.77, 143.90, 143.79, 141.27, 140.07, 139.35, 133.51, 132.21, 129.62, 129.05, 128.16, 127.89, 127.69, 127.06, 125.15, 124.92, 119.96, 99.97, 98.10, 98.00, 67.91, 67.03, 62.14, 51.00, 47.67, 47.11, 30.47, 25.39, 19.25, 18.66.

Synthesis of Ala-NB-PAB-THP

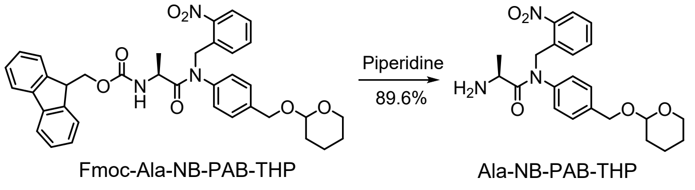

Fmoc-Ala-NB-PAB-THP (1.41 g, 2.21 mmol) was dissolved in 24 mL N,N-dimethylformamide (DMF), and 6 mL piperidine was added. The mixture was stirred under room temperature for

30 minutes. Then, the organic solvents were removed under high vacuum, and the residue was purified by silica gel column chromatography (elution solvents: ethyl acetate/petroleum ether 1:1 to dichloromethane/methanol 10:1 v/v). After drying, 0.82 g Ala-NB-PAB-THP was obtained (89.6% yield). ^{1}H NMR (400 MHz, $CDCl_3$) δ 7.92 (d, *J* = 8.0 Hz, 1H), 7.63 – 7.55 (m, 2H), 7.43 – 7.37 (m, 1H), 7.35 (d, *J* = 8.3 Hz, 2H), 7.10 (d, *J* = 8.3 Hz, 2H), 5.35 – 5.19 (m, 2H), 4.77 (d, *J* = 12.5 Hz, 1H), 4.72 – 4.67 (m, 1H), 4.47 (d, *J* = 12.5 Hz, 1H), 3.93 – 3.84 (m, 1H), 3.55 (ddd, *J* = 15.8, 11.9, 5.7 Hz, 2H), 1.88 – 1.80 (m, 3H), 1.76 – 1.70 (m, 1H), 1.65 – 1.50 (m, 4H), 1.18 (d, *J* = 6.7 Hz, 3H). ^{13}C NMR (101 MHz, $CDCl_3$) δ 177.13, 148.79, 140.75, 139.16, 133.50, 132.66, 129.89, 129.12, 128.28, 127.86, 124.98, 98.20, 98.14, 68.07, 62.35, 50.61, 47.58, 30.62, 25.51, 21.98, 19.44.

Synthesis of Fmoc-Val-Ala-NB-PAB-THP

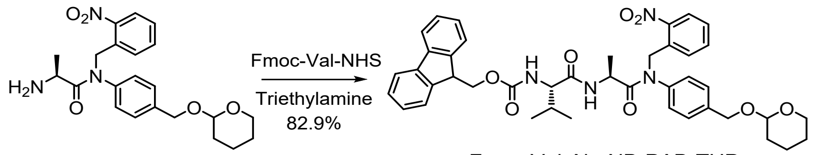

Ala-NB-PAB-THP (0.8 g, 1.93 mmol) and 1.26 g Fmoc-Val-NHS[1] (2.90 mmol) were dissolved in 40 mL dichloromethane, and 0.3 mL triethylamine was added. The mixture was stirred under room temperature overnight. Then, the organic solvents were removed under reduced pressure. After purification by silica gel column chromatography (elution solvents: ethyl acetate/petroleum ether 1:3 to 2:1 v/v), 1.18 g Fmoc-Val-Ala-NB-PAB-THP was obtained as solid foam (82.9% yield). ^{1}H NMR (400 MHz, $CDCl_3$) δ 7.96 (d, *J* = 8.1 Hz, 1H), 7.75 (d, *J* = 7.5 Hz, 2H), 7.60 (dt, *J* = 11.4, 7.1 Hz, 4H), 7.38 (dd, *J* = 7.0, 4.1 Hz, 5H), 7.33 – 7.23 (m, 4H), 6.62 (d, *J* = 6.9 Hz, 1H), 5.44 (dd, *J* = 16.9, 14.1 Hz, 2H), 5.08 (d, *J* = 18.3 Hz, 1H), 4.77 (d, *J* = 12.5 Hz, 1H), 4.70 (dd, *J* = 6.6, 3.1 Hz, 1H), 4.62 (p, *J* = 6.9 Hz, 1H), 4.47 (d, *J* = 12.5 Hz, 1H), 4.38 (dt, *J* = 17.5, 10.5 Hz, 2H), 4.20 (t, *J* = 7.0 Hz, 1H), 4.06 (dd, *J* = 8.5, 6.3 Hz, 1H), 3.88 (t, *J* = 10.0 Hz, 1H), 3.60 – 3.50 (m, 1H), 2.13 (td, *J* = 13.1, 6.6 Hz, 1H), 1.91 – 1.80 (m, 1H), 1.71 (m, 1H), 1.68 – 1.51 (m, 4H), 1.21 (d, *J* = 6.9 Hz, 3H), 0.97 (dd, *J* = 10.3, 6.9 Hz, 6H). ^{13}C NMR (101 MHz, $CDCl_3$) δ 173.37, 170.93, 156.30, 148.41, 143.94, 143.78, 141.30, 140.22, 139.39, 139.36, 133.49, 132.27, 129.49, 129.08, 128.12, 127.89, 127.70, 127.07, 125.11, 124.99, 119.98, 119.96, 98.12, 98.02, 68.00, 67.94, 67.07, 62.16, 62.14, 59.93, 51.29, 47.21, 46.40, 33.97, 31.58, 30.50, 25.63, 25.42, 24.96, 19.29, 19.27, 19.10, 17.95, 17.84.

Synthesis of Fmoc-Val-Ala-NB-PAB-OH

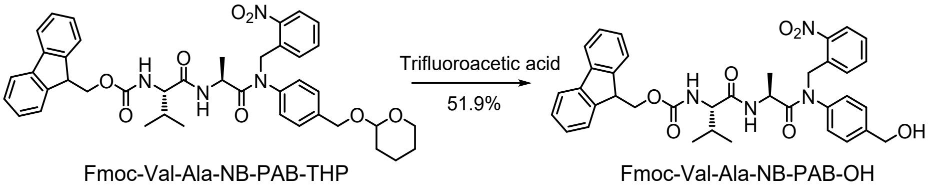

1.09 g Fmoc-Val-Ala-NB-PAB-THP (1.56 mmol) was dissolved in 10 mL dichloromethane, and 10 mL trifluoroacetic acid was added. The mixture was stirred under room temperature for two

hours. Then, the organic solvents were removed under high vacuum. The residue was purified by silica gel column chromatography (elution solvents: ethyl acetate/petroleum ether 1:1 v/v to ethyl acetate). After purification, 0.53 g Fmoc-Val-Ala-NB-PAB-OH was obtained (51.9% yield). ^{1}H NMR (400 MHz, $CDCl_3$) δ 7.94 (d, *J* = 8.0 Hz, 1H), 7.75 (d, *J* = 7.5 Hz, 2H), 7.58 (dd, *J* = 11.4, 6.3 Hz, 4H), 7.38 (t, *J* = 7.7 Hz, 5H), 7.27 (dt, *J* = 17.2, 8.1 Hz, 4H), 6.65 (d, *J* = 4.7 Hz, 1H), 5.53 – 5.37 (m, 2H), 5.10 (d, *J* = 16.5 Hz, 1H), 4.67 (s, 2H), 4.57 (p, *J* = 6.8 Hz, 1H), 4.37 (dt, *J* = 17.2, 10.4 Hz, 2H), 4.18 (t, *J* = 6.9 Hz, 1H), 4.07 – 4.00 (m, 1H), 2.49 – 2.30 (m, 1H), 2.12 (dt, *J* = 15.5, 8.7 Hz, 1H), 1.20 (d, *J* = 6.8 Hz, 3H), 0.96 (dd, *J* = 9.6, 7.1 Hz, 6H). ^{13}C NMR (101 MHz, $CDCl_3$) δ 173.47, 171.19, 156.49, 148.62, 144.03, 143.87, 141.91, 141.42, 133.61, 132.19, 129.81, 128.52, 128.35, 128.18, 127.84, 127.20, 125.21, 125.08, 120.10, 67.23, 64.49, 60.17, 60.14, 51.18, 47.30, 46.55, 31.57, 19.25, 18.05, 17.96, 17.93.

Synthesis of Fmoc-Val-Ala-NB-PAB-DMT

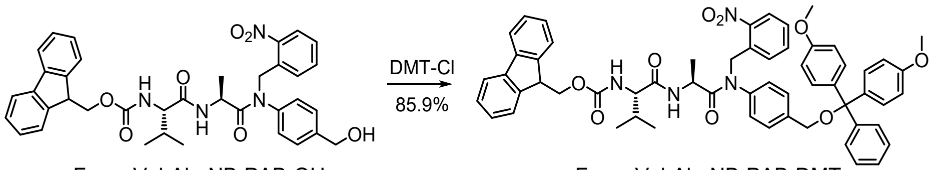

Fmoc-Val-Ala-NB-PAB-OH Fmoc-Val-Ala-NB-PAB-DMT

Fmoc-Val-Ala-NB-PAB-OH (0.51 g, 0.78 mmol) was dissolved in 20 mL anhydrous pyridine under argon gas protection. 4,4'-Dimethoxytrityl chloride (DMT-Cl) (0.53 g, 1.56 mmol) was added, and the mixture was stirred overnight. Then, the solvent was removed under high vacuum, and the residue was purified by silica gel column chromatography (elution solvents: ethyl acetate/petroleum ether 1:2 to 1:1 v/v and 1% triethylamine was added). After purification, 0.64 g Fmoc-Val-Ala-NB-PAB-DMT was obtained as solid foam (85.9% yield). ^{1}H NMR (400 MHz, $CDCl_3$) δ 7.98 (d, *J* = 8.1 Hz, 1H), 7.75 (d, *J* = 7.4 Hz, 2H), 7.67 – 7.55 (m, 4H), 7.49 (dd, *J* = 5.2, 3.4 Hz, 2H), 7.44 – 7.34 (m, 9H), 7.33 – 7.25 (m, 5H), 7.25 – 7.18 (m, 2H), 6.87 – 6.77 (m, 4H), 6.55 (d, *J* = 7.0 Hz, 1H), 5.44 (dd, *J* = 22.9, 12.8 Hz, 2H), 5.09 (d, *J* = 16.7 Hz, 1H), 4.64 (p, *J* = 6.9 Hz, 1H), 4.39 (ddd, *J* = 17.5, 10.5, 7.5 Hz, 2H), 4.24 – 4.16 (m, 3H), 4.06 (dd, *J* = 8.6, 6.2 Hz, 1H), 3.78 (s, 6H), 2.19 – 2.09 (m, 1H), 1.23 (d, *J* = 7.0 Hz, 3H), 0.98 (dd, *J* = 9.8, 7.0 Hz, 6H). ^{13}C NMR (101 MHz, $CDCl_3$) δ 173.52, 158.66, 156.42, 148.51, 144.94, 144.04, 143.90, 141.43, 140.42, 139.93, 136.13, 133.64, 132.47, 130.15, 129.53, 128.38, 128.25, 128.03, 127.83, 127.20, 126.98, 120.10, 113.32, 86.69, 67.18, 64.81, 60.08, 55.35, 51.55, 47.33, 46.56, 31.70, 19.24, 18.13, 17.96.

Synthesis of Val-Ala-NB-PAB-DMT

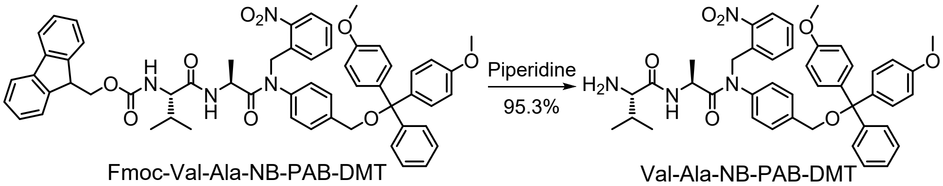

Fmoc-Val-Ala-NB-PAB-DMT Val-Ala-NB-PAB-DMT

Fmoc-Val-Ala-NB-PAB-DMT (0.61 g, 0.64 mmol) was dissolved in 8 mL DMF, and 2 mL piperidine was added. The mixture was stirred under room temperature for 30 minutes. Then, the solvent was removed under high vacuum, and the residue was purified by silica gel column chromatography (elution solvents: ethyl acetate/petroleum ether 1:1 to dichloromethane/methanol 5:1 v/v and 1% triethylamine were added). After purification, 0.45 g Val-Ala-NB-PAB-DMT was obatined as solid foam (95.3% yield). ^{1}H NMR (400 MHz, $CDCl_3$) δ 7.96 (d, *J* = 8.9 Hz, 1H), 7.73 (d, *J* = 7.8 Hz, 1H), 7.69 – 7.58 (m, 2H), 7.48 (dd, *J* = 5.2, 3.3 Hz, 2H), 7.43 – 7.33 (m, 7H), 7.31 – 7.18 (m, 5H), 6.87 – 6.78 (m, 4H), 5.42 (d, *J* = 16.7 Hz, 1H), 5.14 (d, *J* = 16.7 Hz, 1H), 4.69 (p, *J* = 7.0 Hz, 1H), 4.16 (s, 2H), 3.78 (s, 6H), 3.24 (d, *J* = 3.9 Hz, 1H), 2.24 (dtd, *J* = 13.8, 6.9, 4.0 Hz, 1H), 1.24 (d, *J* = 6.9 Hz, 3H), 0.98 (d, *J* = 7.0 Hz, 3H), 0.82 (d, *J* = 6.9 Hz, 3H). ^{13}C NMR (101 MHz, $CDCl_3$) δ 174.40, 174.03, 158.65, 148.53, 144.95, 140.21, 140.02, 136.15, 133.75, 132.61, 130.15, 129.62, 128.34, 128.26, 128.17, 128.02, 127.91, 126.96, 125.08, 113.31, 86.66, 64.82, 60.28, 55.35, 51.39, 46.22, 45.96, 31.16, 19.81, 18.55, 16.28.

Synthesis of HDA-Val-Ala-NB-PAB-DMT

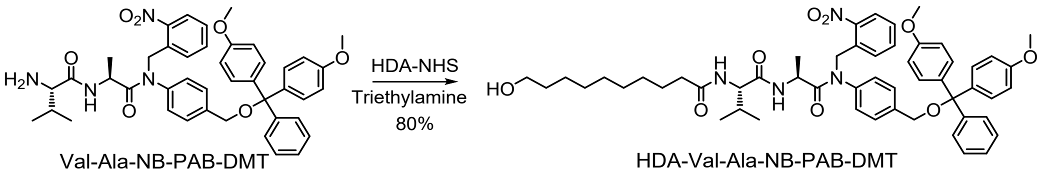

Val-Ala-NB-PAB-DMT (0.43 g, 0.59 mmol) and 0.25 g NHS-protected 10-hydroxydecanoic acid (HDA-NHS)[1] were dissolved in 25 mL dichloromethane, and 0.25 mL triethylamine was added. The mixture was stirred under room temperature overnight. Then, the organic solvents were removed under reduced pressure. After purification by silica gel column chromatography (elution solvents: ethyl acetate/petroleum ether 1:1 to dichloromethane/ methanol 10:1 v/v and 1% triethylamine was added), 0.43 g HDA-Val-Ala-NB-PAB-DMT was obtained as solid foam (80% yield). ^{1}H NMR (400 MHz, $CDCl_3$) δ 7.97 – 7.84 (m, 1H), 7.61 – 7.50 (m, 2H), 7.44 – 7.37 (m, 2H), 7.38 – 7.26 (m, 7H), 7.17 (dddd, *J* = 9.2, 7.3, 6.2, 1.4 Hz, 5H), 6.76 (dd, *J* = 9.4, 2.5 Hz, 4H), 6.54 (d, *J* = 7.1 Hz, 1H), 6.03 (d, *J* = 8.7 Hz, 1H), 5.38 (d, *J* = 16.8 Hz, 1H), 5.02 (d, *J* = 16.8 Hz, 1H), 4.53 (p, *J* = 6.9 Hz, 1H), 4.26 (dd, *J* = 8.7, 6.4 Hz, 1H), 4.10 (s, 2H), 3.71 (s, 6H), 3.60 – 3.50 (m, 2H), 2.13 (t, *J* = 7.6 Hz, 2H), 2.02 (dt, *J* = 17.1, 5.2 Hz, 1H), 1.50 (ddd, *J* = 20.8, 12.8, 5.6 Hz, 4H), 1.28 – 1.12 (m, 13H), 0.89 (t, *J* = 7.1 Hz, 6H). ^{13}C NMR (101 MHz, $CDCl_3$) δ 173.37, 173.05, 171.05, 158.53, 148.38, 144.81, 140.25, 139.87, 136.00, 133.51, 132.39, 130.02, 129.40, 128.23, 128.12, 128.09, 127.89, 127.70, 126.85, 125.03, 113.19, 86.56, 64.68, 62.96, 57.78, 55.22, 51.42, 46.41, 36.74, 32.74, 31.58, 29.29, 29.24, 29.16, 29.11, 25.64, 19.12, 18.13, 17.87.

Synthesis of Val-Ala(NB) phosphoramidite

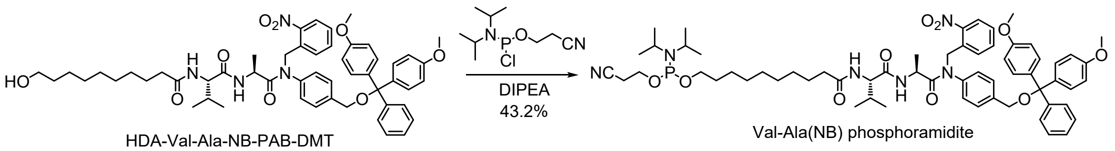

HDA-Val-Ala-NB-PAB-DMT (0.4 g, 0.44 mmol) was dissolved in 20 mL anhydrous dichloromethane under argon gas protection, and 0.4 mL DIPEA was added. The mixture was cooled down under ice bath. Then, 0.2 mL 2-cyanoethyl N,N-diisopropylchlorophosphoramidite (0.9 mmol) was added dropwise. The reaction was monitored by thin-layer chromatography. When the reaction was completed, the mixture was diluted with 50 mL dichloromethane and washed with saturated KCl. The organic layer was collected and dried by anhydrous Na_2SO_4. After purification by silica gel column chromatography (elution solvents: ethyl acetate with 1% triethylamine), 0.21 g Val-Ala(NB) phosphoramidite was obtained as solid foam (43.2% yield). ^{1}H NMR (400 MHz, $CDCl_3$) δ 7.98 (d, *J* = 7.9 Hz, 1H), 7.67 – 7.58 (m, 2H), 7.48 (dd, *J* = 5.2, 3.3 Hz, 2H), 7.45 – 7.34 (m, 7H), 7.32 – 7.27 (m, 2H), 7.26 – 7.19 (m, 3H), 6.86 – 6.80 (m, 4H), 6.33 (d, *J* = 7.1 Hz, 1H), 5.99 (d, *J* = 8.6 Hz, 1H), 5.44 (d, *J* = 16.7 Hz, 1H), 5.10 (d, *J* = 16.8 Hz, 1H), 4.61 (p, *J* = 6.8 Hz, 1H), 4.29 (dd, *J* = 8.6, 6.3 Hz, 1H), 4.17 (s, 2H), 3.90 – 3.79 (m, 2H), 3.78 (s, 6H), 3.67 – 3.53 (m, 4H), 2.63 (t, *J* = 6.6 Hz, 2H), 2.20 (t, *J* = 7.6 Hz, 2H), 2.16 – 2.05 (m, 1H), 1.66 – 1.53 (m, 4H), 1.35 – 1.22 (m, 13H), 1.18 (dd, *J* = 6.8, 4.9 Hz, 12H), 0.97 (t, *J* = 6.5 Hz, 6H). ^{31}P NMR (162 MHz, $CDCl_3$) δ 147.17.

4.5 Synthesis of CA4 phosphoramidite

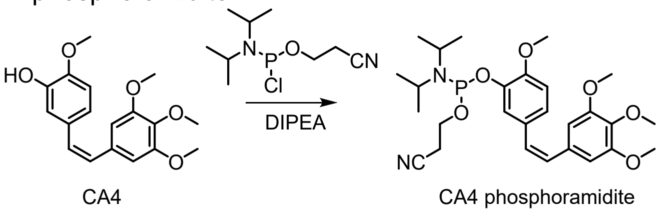

Figure S38. Synthesis of CA4 phosphoramidite.

Combretastatin A-4 (CA4) (0.5 g, 1.58 mmol) was dissolved in 20 mL anhydrous dichloromethane under argon gas protection, and 0.55 mL DIPEA (3.16 mmol) was added. The mixture was cooled down under ice bath. Then, 0.5 mL 2-cyanoethyl N,N-diisopropylchlorophosphoramidite was added dropwise. The reaction was monitored by thin-layer chromatography. When the reaction was completed, the mixture was diluted with 50 mL dichloromethane and washed with saturated KCl. The organic layer was collected and dried by anhydrous Na_2SO_4. After purification by silica gel column chromatography (elution solvents: ethyl acetate/petroleum ether 1:4 to 1:2 v/v and 1% triethylamine was added), 0.78 g CA4 phosphoramidite was obtained (95.5% yield). ^{1}H NMR (400 MHz, $CDCl_3$) δ 7.02 (t, *J* = 1.7 Hz, 1H), 6.95 (dd, *J* = 8.4, 2.1 Hz, 1H), 6.78 (d, *J* = 8.4 Hz, 1H), 6.50 (s, 2H), 6.43 (dd, *J* = 12.1 Hz,

2H), 3.96 – 3.86 (m, 2H), 3.83 (s, 3H), 3.81 (s, 3H), 3.74 – 3.64 (m, 8H), 2.67 (t, *J* = 6.6 Hz, 2H), 1.19 (d, *J* = 6.8 Hz, 6H), 1.10 (d, *J* = 6.8 Hz, 6H). ^{31}P NMR (162 MHz, $CDCl_3$) δ 149.01. ^{13}C NMR (101 MHz, $CDCl_3$) δ 153.06, 150.78, 150.75, 143.06, 142.98, 137.16, 132.92, 130.03, 129.39, 129.09, 124.47, 121.98, 121.88, 117.82, 111.94, 106.05, 60.99, 59.39, 59.21, 56.03, 55.91, 43.96, 43.82, 24.76, 24.68, 24.36, 24.29, 20.33, 20.27.

4.6 Synthesis of 4MU phosphoramidite

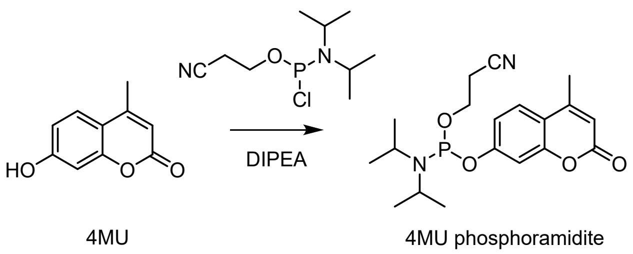

Figure S39. Synthesis of 4MU phosphoramidite.

4-Methylumbelliferone (4MU) (0.5 g, 2.83 mmol) was dissolved in a mixed solvents with 10 mL dry tetrahydrofuran and 20 mL dry dichloromethane under argon gas protection, and 0.9 mL DIPEA (0.66 g, 5.16 mmol) was added. The mixture was cooled down under ice bath. Then, 0.75 mL 2-cyanoethyl N,N-diisopropylchlorophosphoramidite (0.79 g, 3.36 mmol) was added dropwise. The reaction was monitored by thin-layer chromatography. When the reaction was completed, the mixture was diluted with 100 mL dichloromethane and washed with 50 mL saturated KCl. The organic layer was collected and dried by anhydrous sodium sulfate. After purification by silica gel column chromatography (elution solvents: ethyl acetate/petroleum ether 1:1 v/v and 1% triethylamine was added), 0.637 g 4MU phosphoramidite was obtained as colorless oily (59.7% yield). ^{1}H NMR (400 MHz, $CDCl_3$) δ 7.43 (d, *J* = 8.3 Hz, 1H), 6.93 (dtd, *J* = 3.7, 2.4, 1.5 Hz, 2H), 6.10 (d, *J* = 1.2 Hz, 1H), 3.95 – 3.84 (m, 2H), 3.73 – 3.61 (m, 2H), 2.63 (t, *J* = 6.4 Hz, 2H), 2.34 (d, *J* = 1.2 Hz, 3H), 1.18 (d, *J* = 6.8 Hz, 6H), 1.11 (d, *J* = 6.8 Hz, 6H). ^{31}P NMR (162 MHz, $CDCl_3$) δ 147.45. ^{13}C NMR (101 MHz, $CDCl_3$) δ 161.16, 157.69, 157.61, 154.76, 152.39, 125.52, 117.31, 116.49, 116.40, 115.00, 112.76, 107.48, 107.37, 59.21, 59.03, 43.98, 43.85, 24.65, 24.58, 24.46, 24.39, 20.41, 20.33, 18.71.

5. NMR spectra

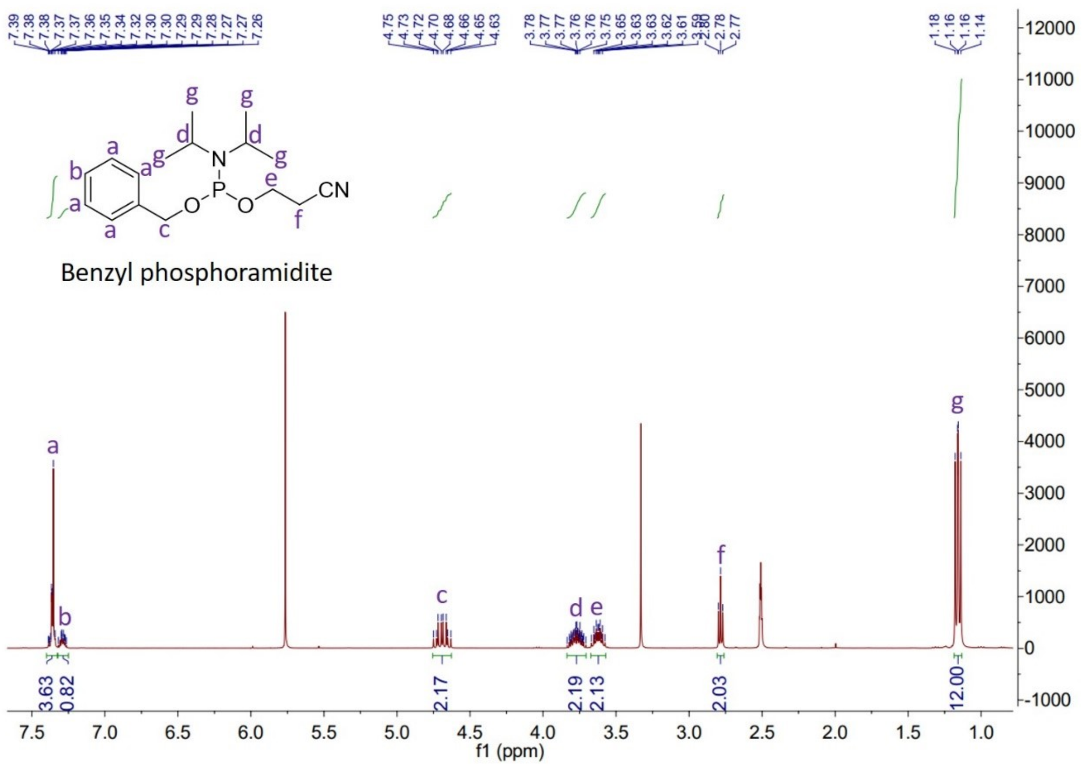

Figure S40. ^{1}H NMR of benzyl phosphoramidite.

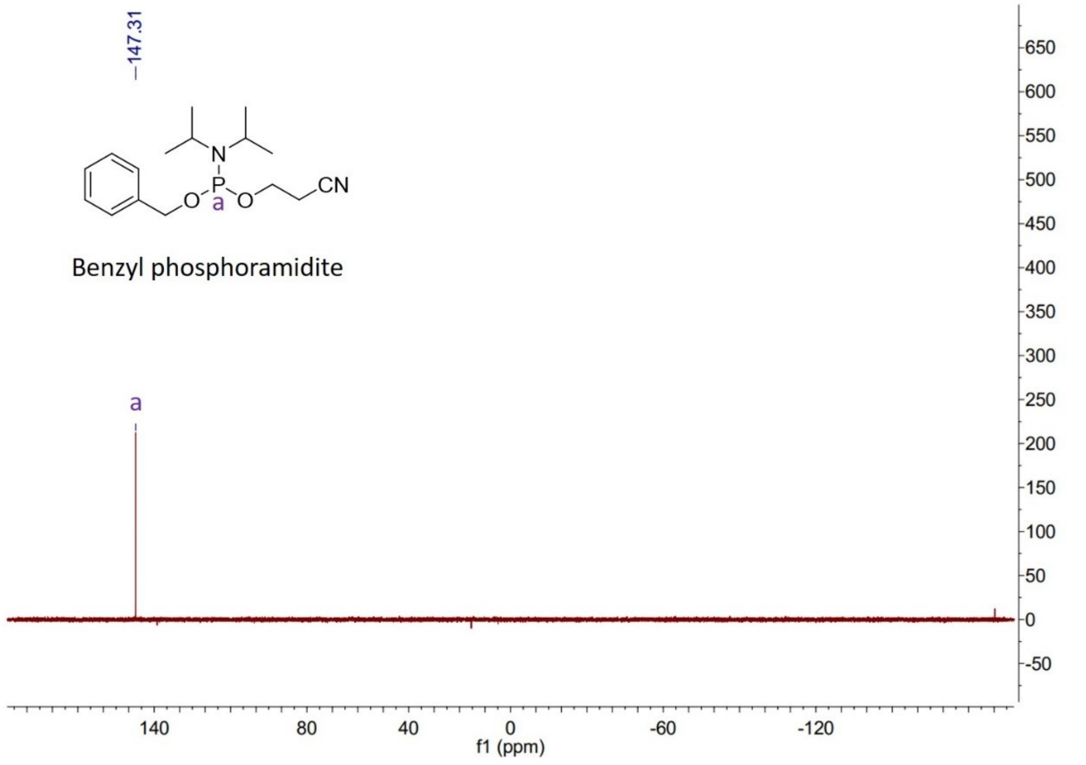

Figure S41. ^{31}P NMR of benzyl phosphoramidite.

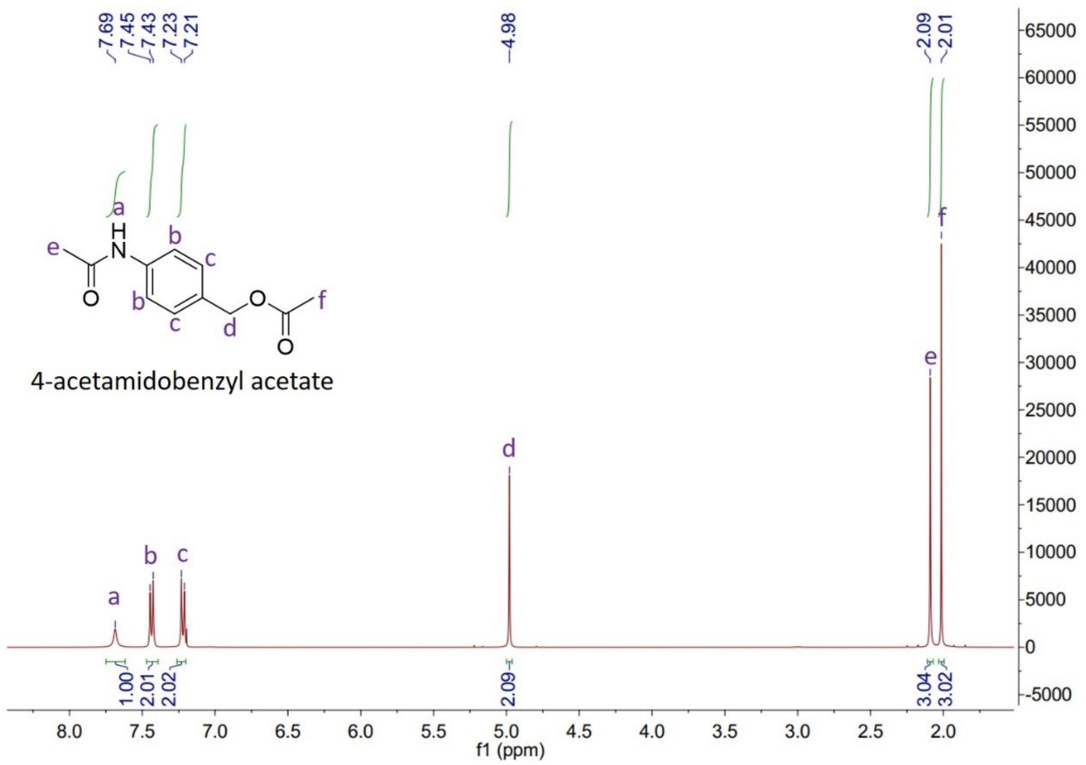

Figure S42. ^{1}H NMR of 4-acetamidobenzyl acetate.

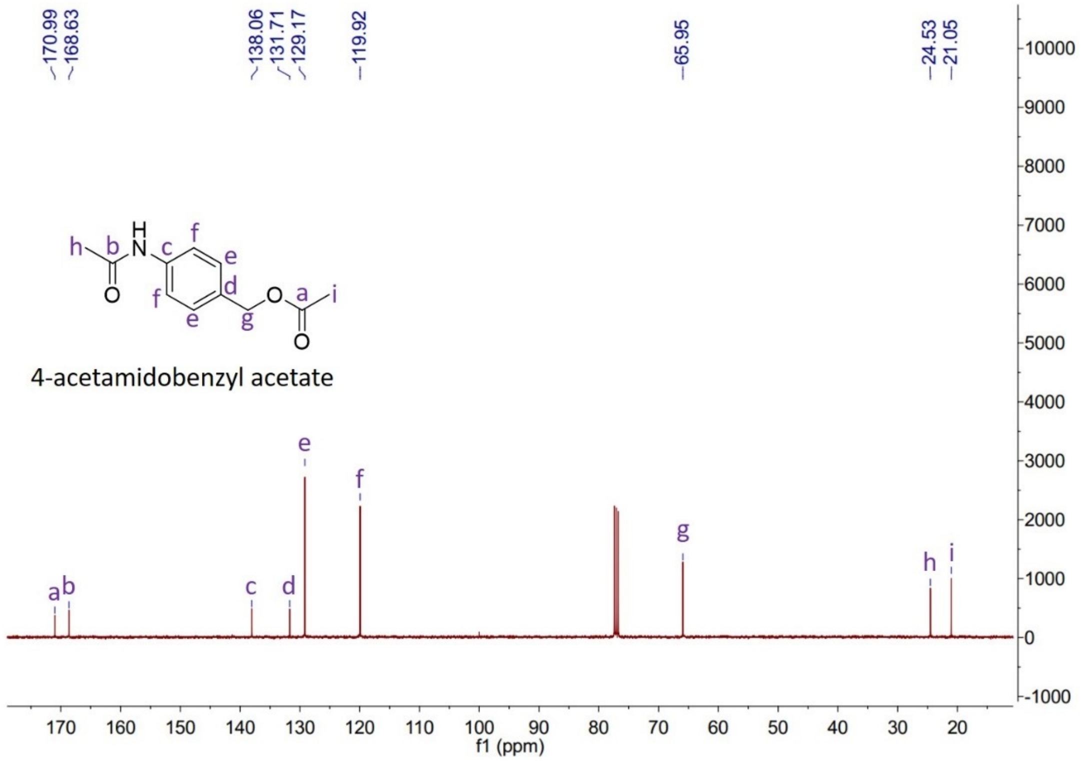

Figure S43. ^{13}C NMR of 4-acetamidobenzyl acetate.

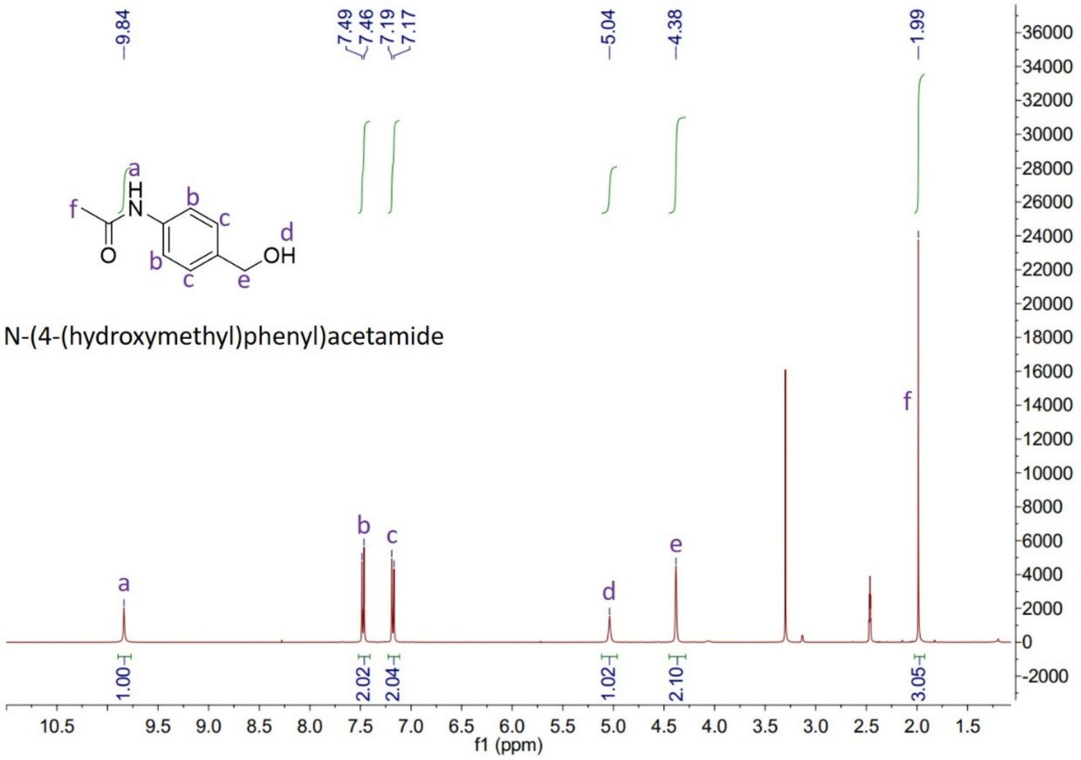

Figure S44. ^{1}H NMR of N-(4-(hydroxymethyl)phenyl)acetamide.

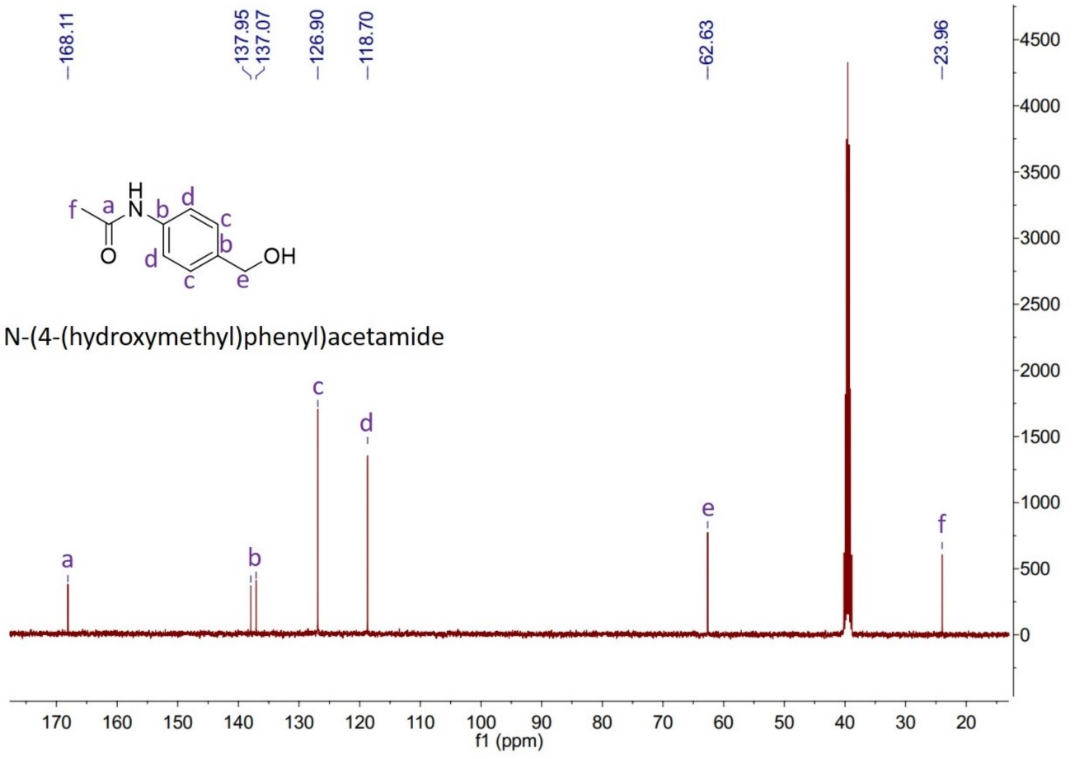

Figure S45. ^{13}C NMR of N-(4-(hydroxymethyl)phenyl)acetamide.

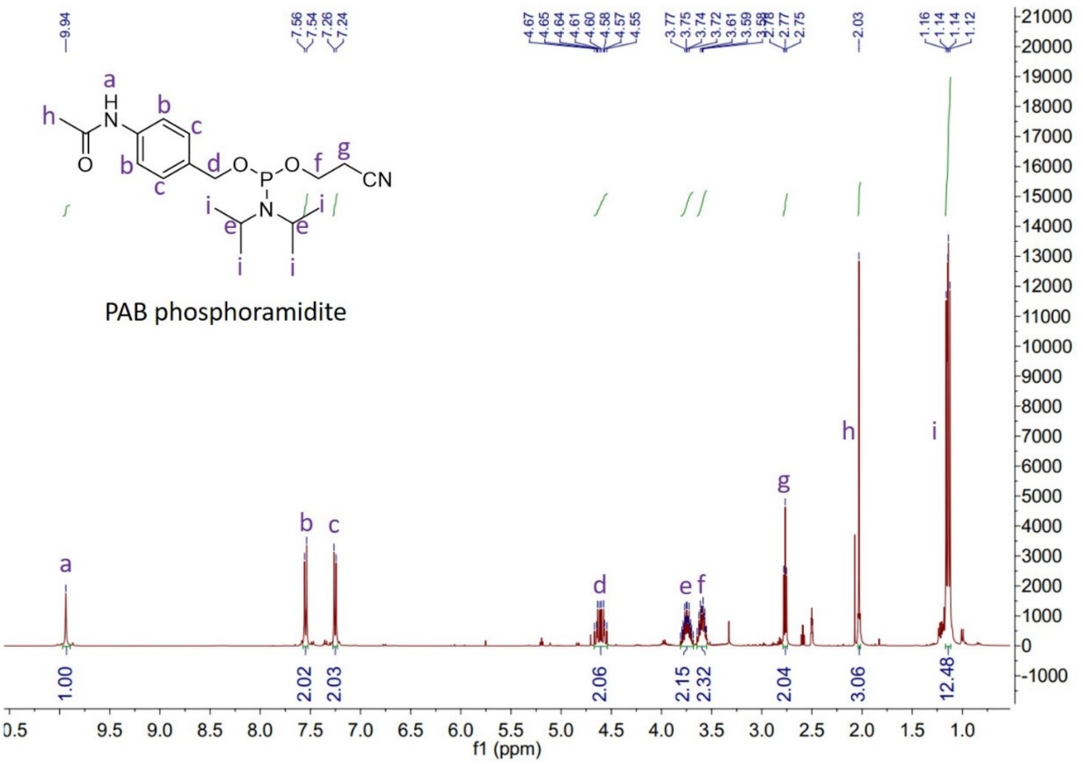

Figure S46. ^{1}H NMR of PAB phosphoramidite.

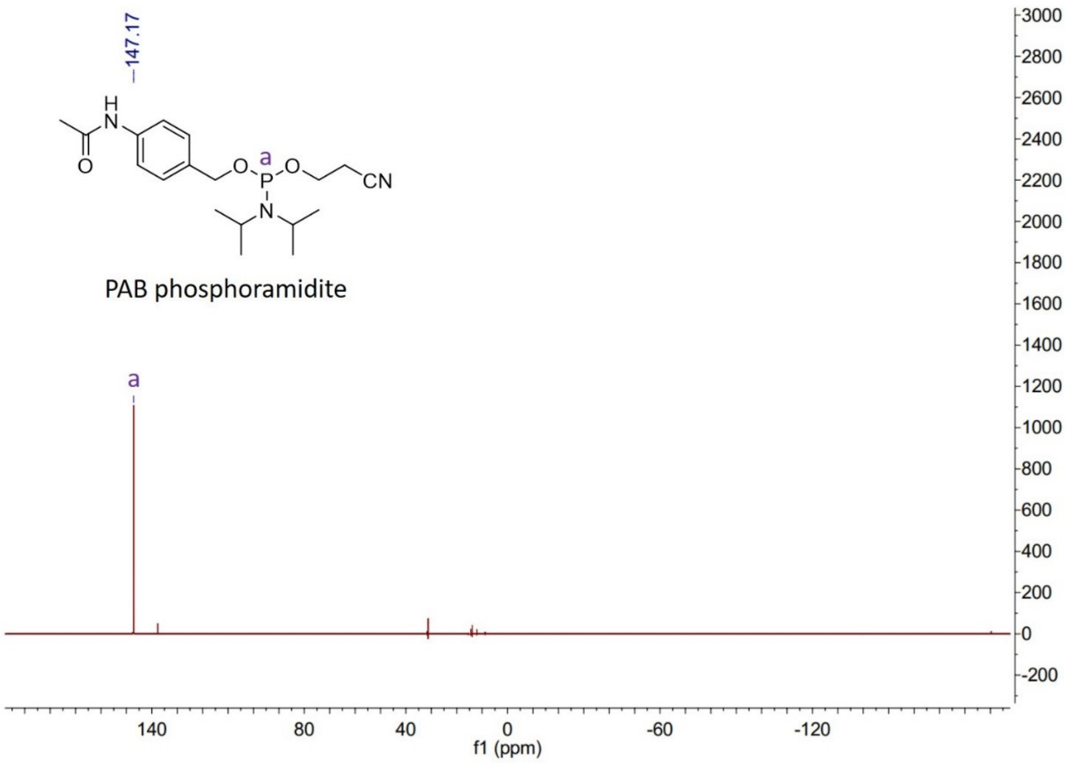

Figure S47. ^{31}P NMR of PAB phosphoramidite.

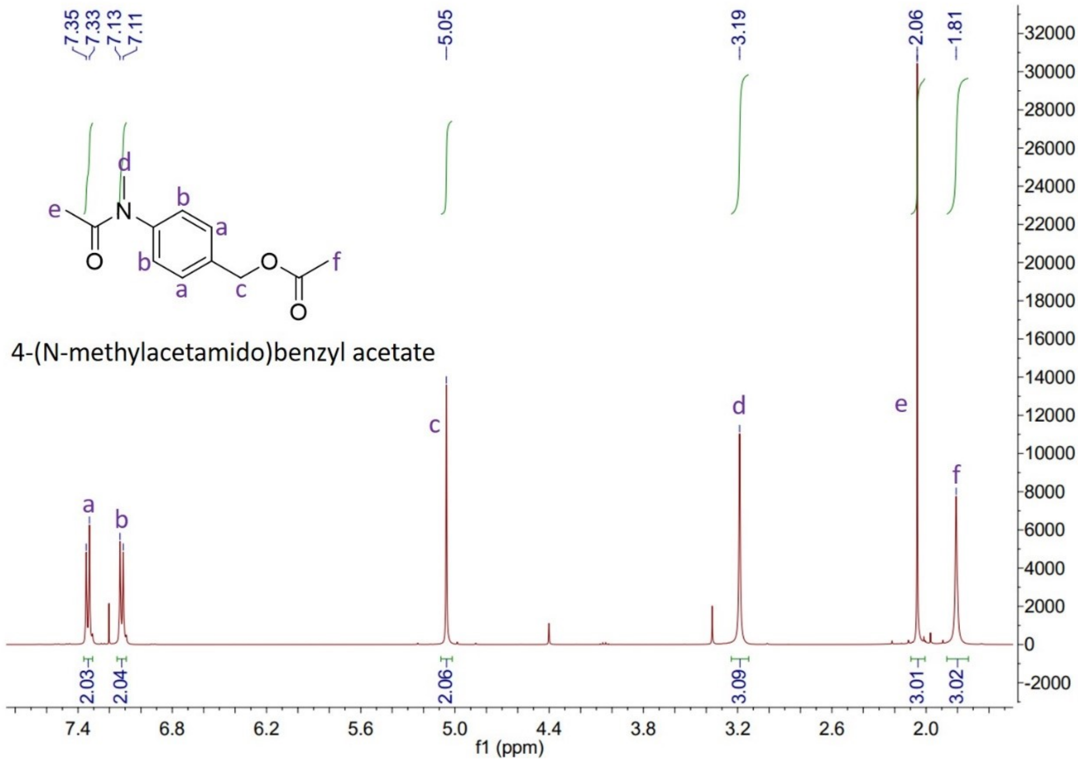

Figure S48. ^{1}H NMR of 4-(N-methylacetamido)benzyl acetate.

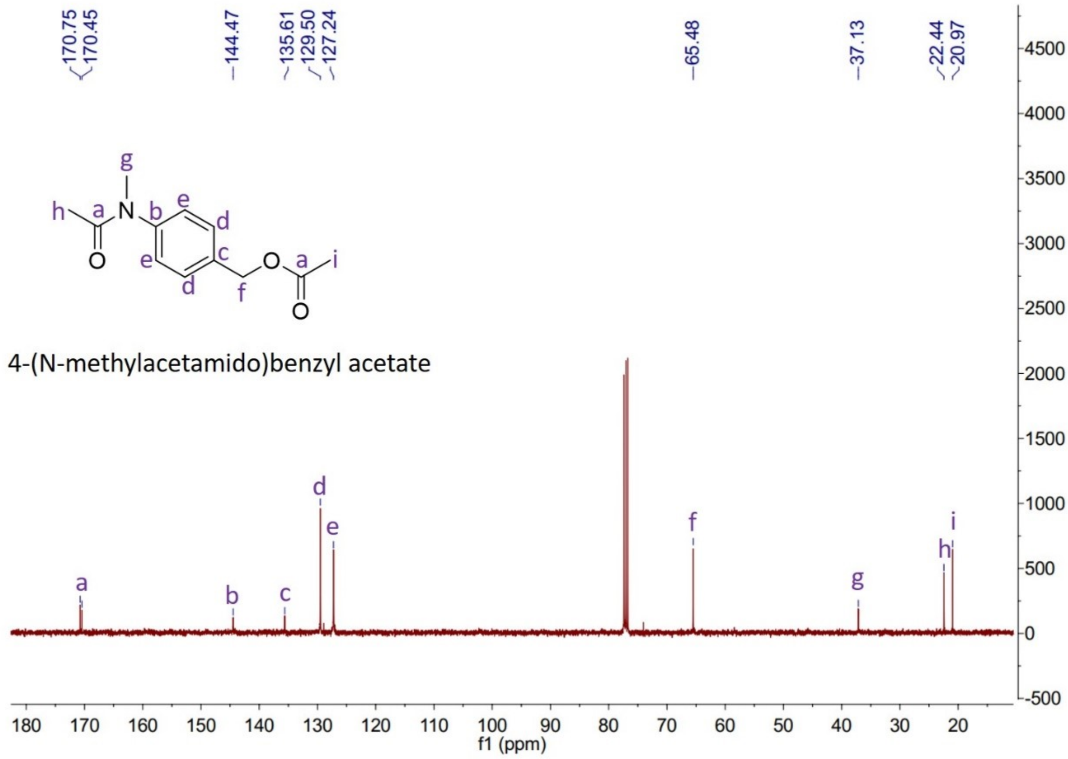

Figure S49. ^{13}C NMR of 4-(N-methylacetamido)benzyl acetate.

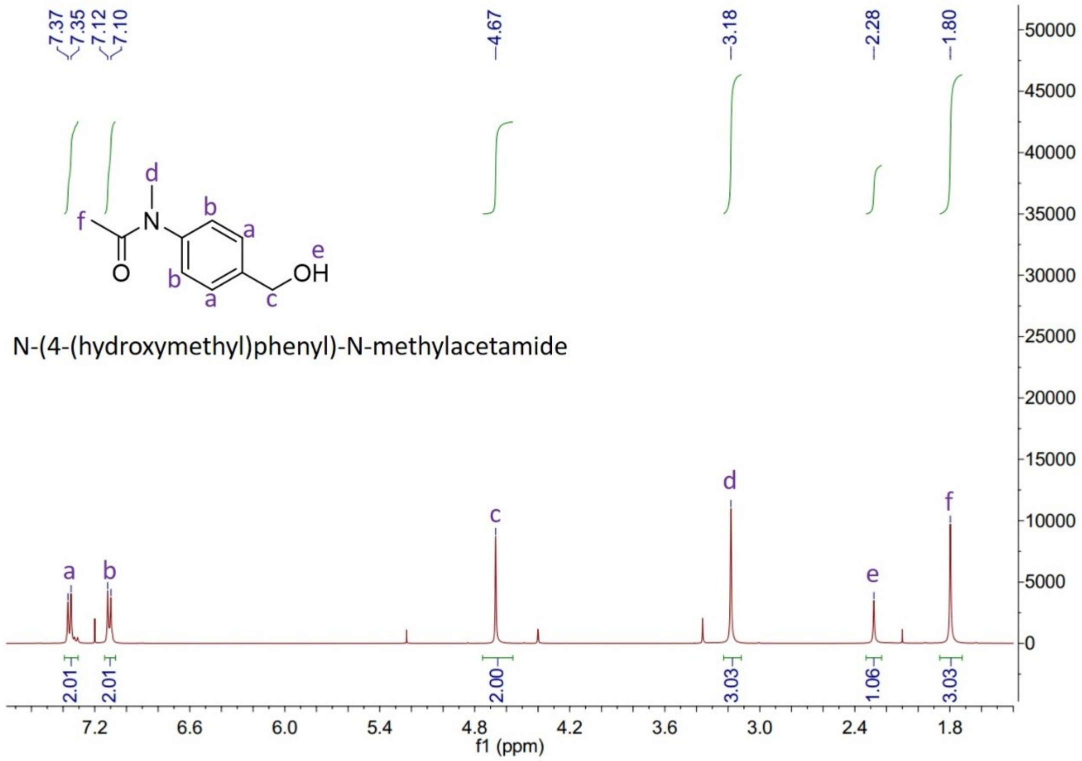

Figure S50. ^{1}H NMR of N-(4-(hydroxymethyl)phenyl)-N-methylacetamide.

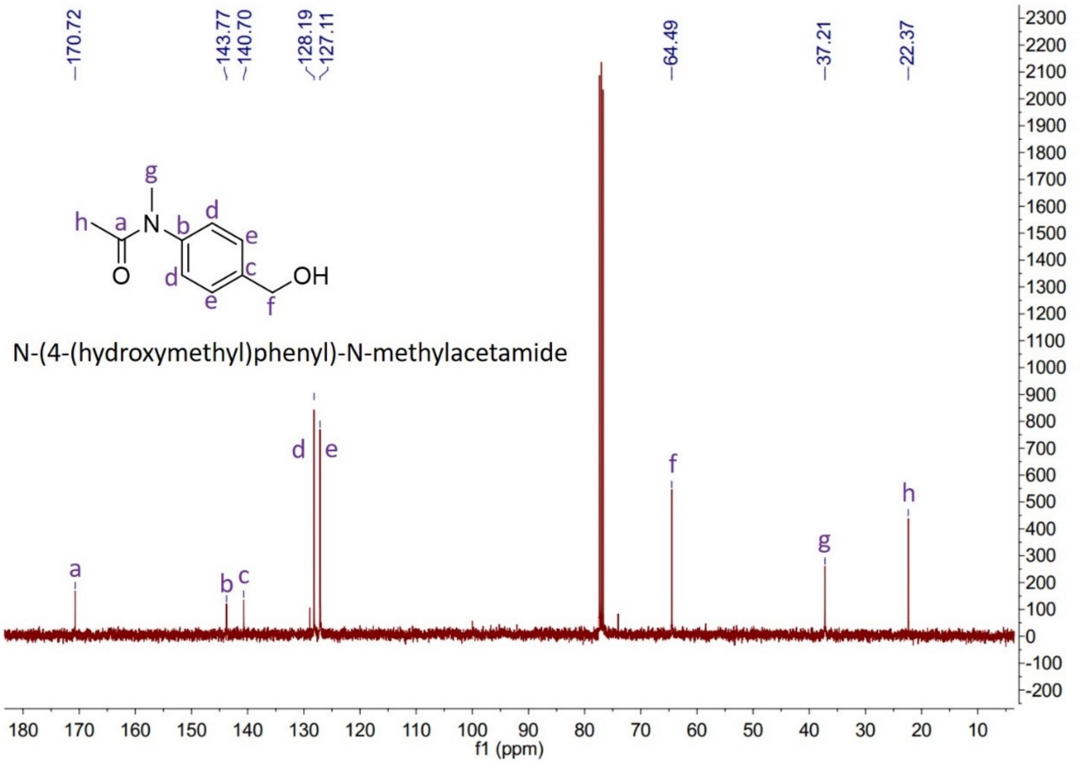

Figure S51. ^{13}C NMR of N-(4-(hydroxymethyl)phenyl)-N-methylacetamide.

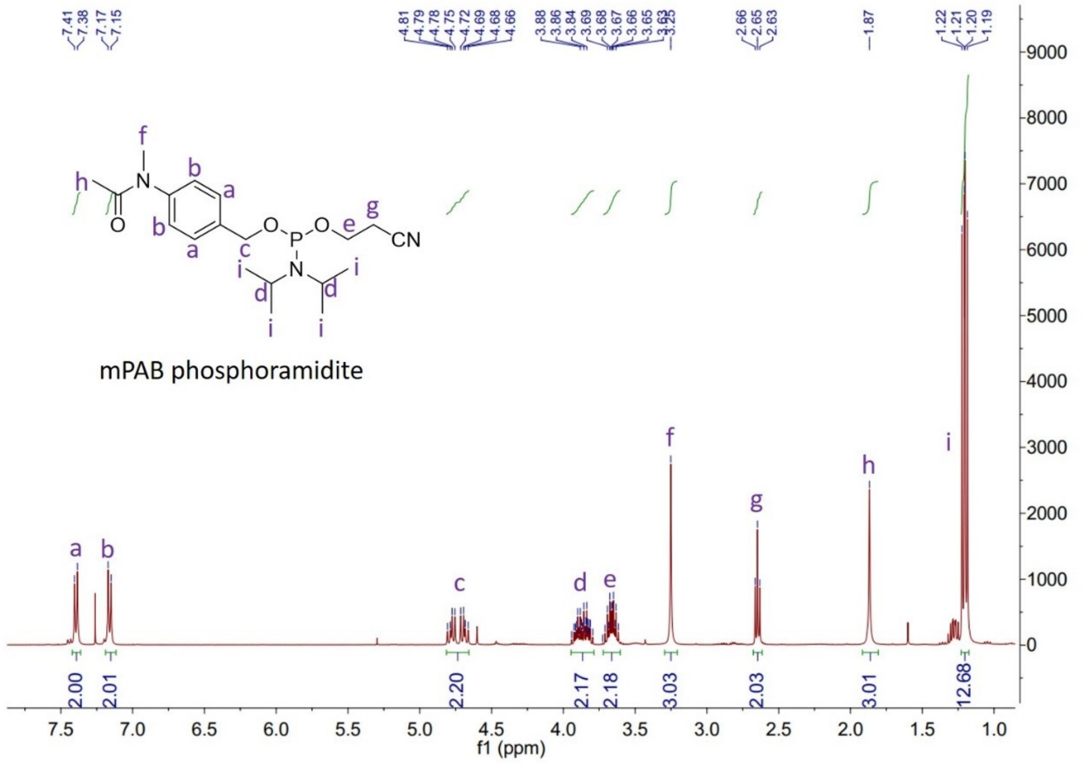

Figure S52. ^{1}H NMR of mPAB phosphoramidite.

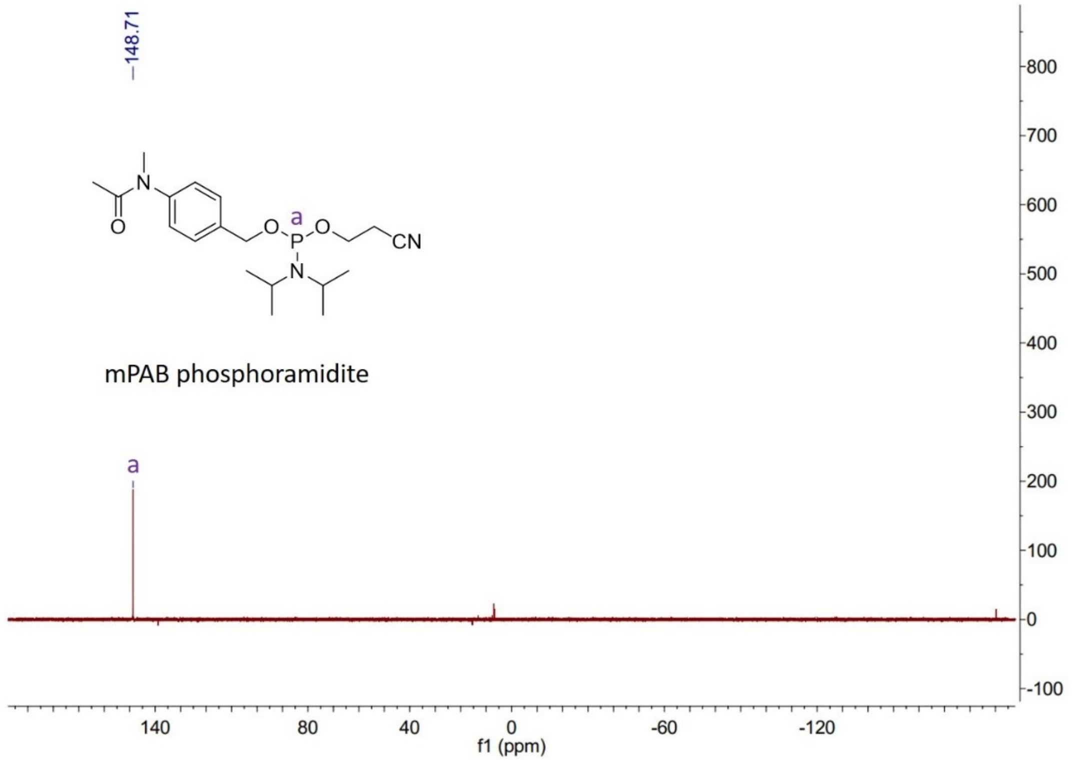

Figure S53. ^{31}P NMR of mPAB phosphoramidite.

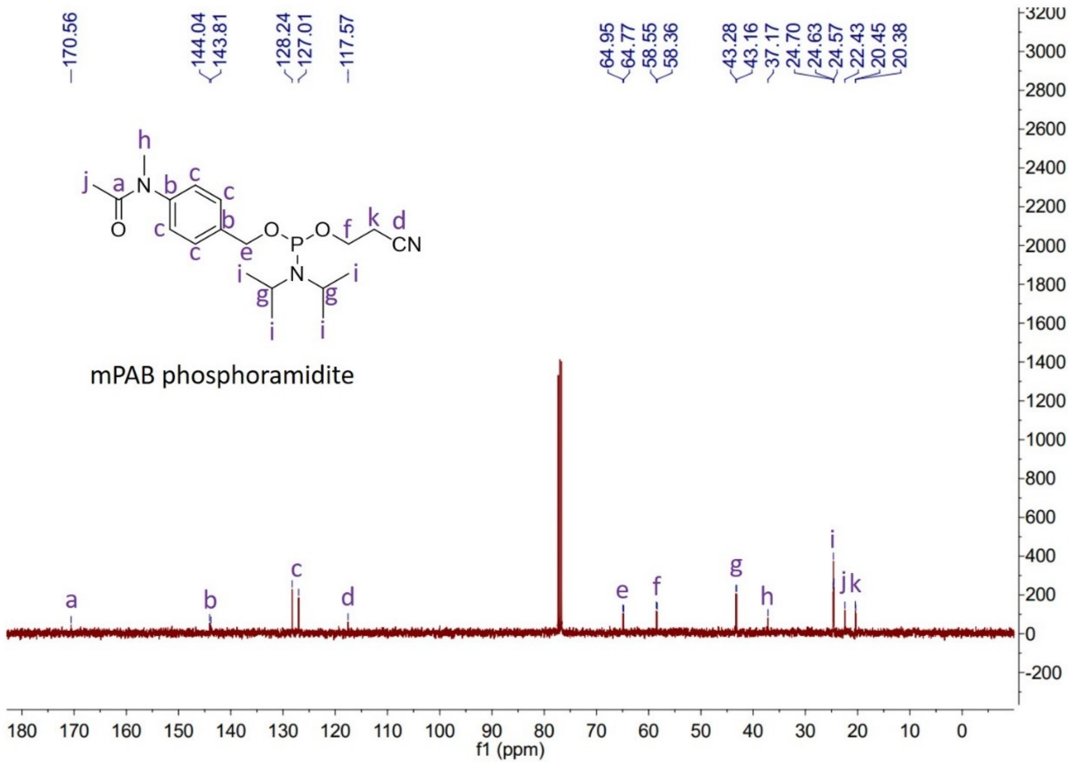

Figure S54. ^{13}C NMR of mPAB phosphoramidite.

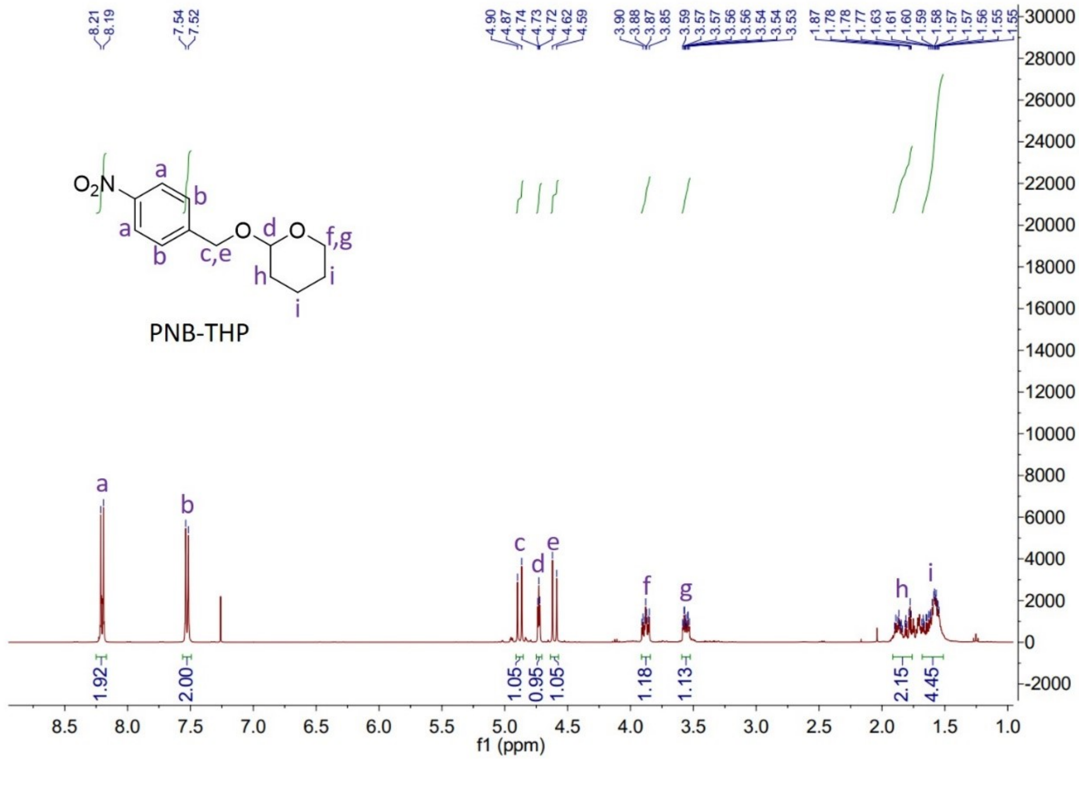

Figure S55. ^{1}H NMR of PNB-THP.

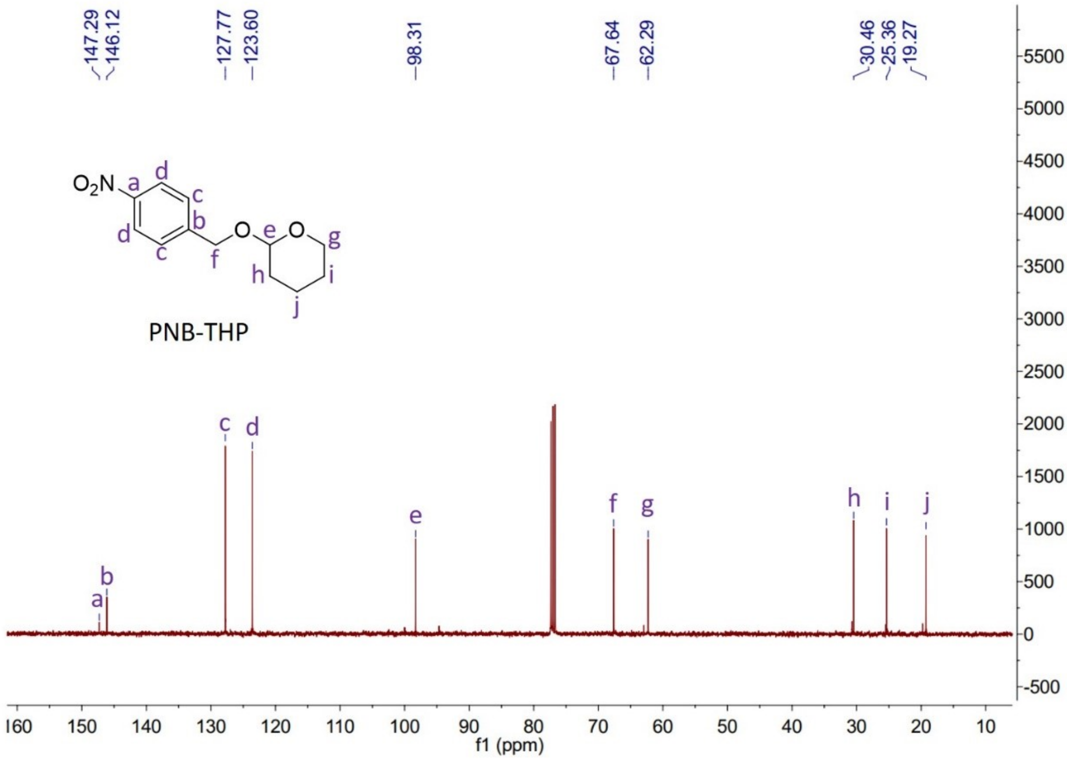

Figure S56. ^{13}C NMR of PNB-THP.

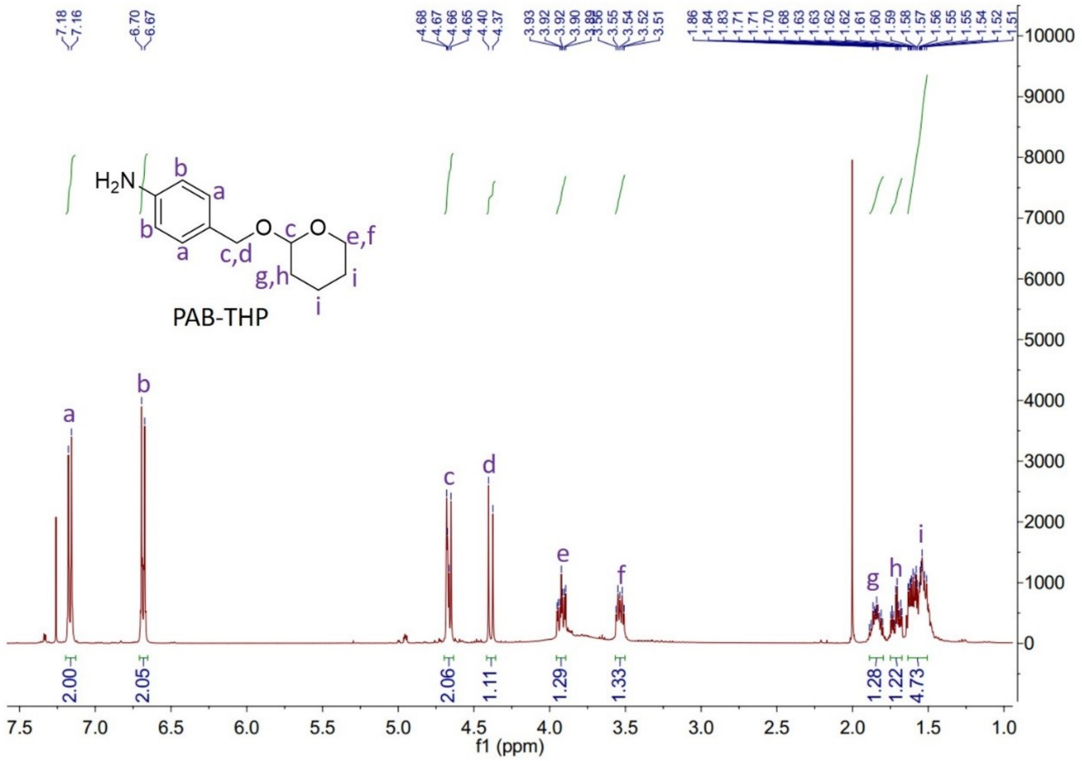

Figure S57. ^{1}H NMR of PAB-THP.

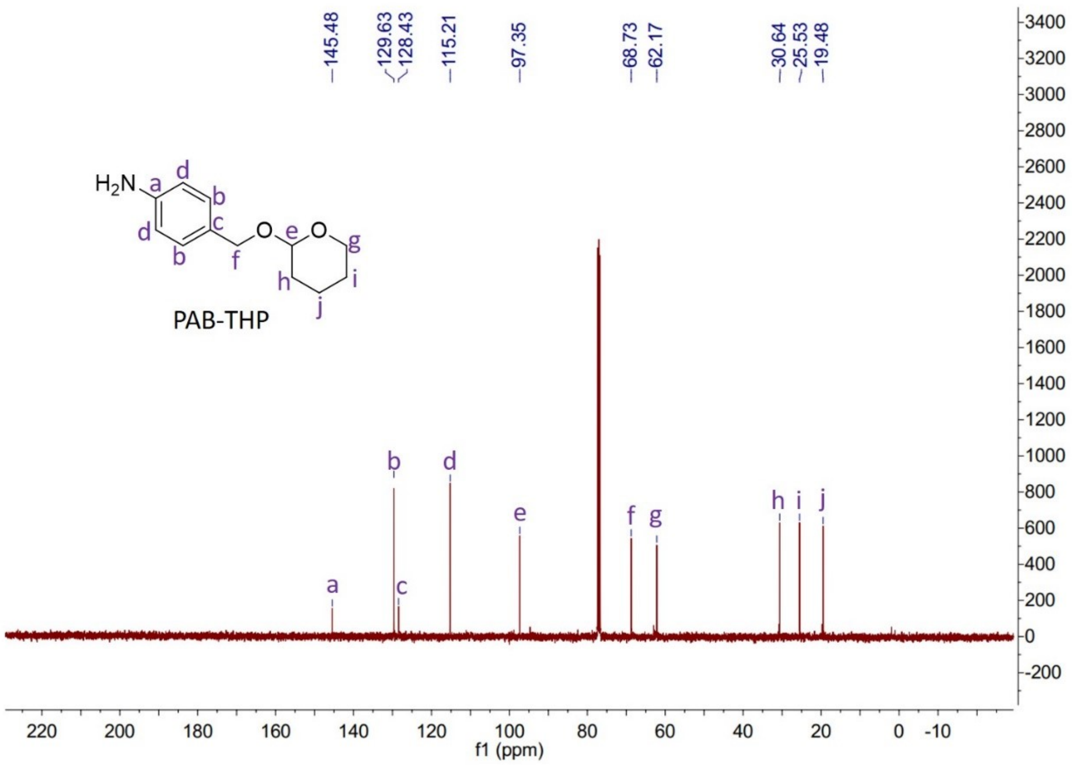

Figure S58. ^{13}C NMR of PAB-THP.

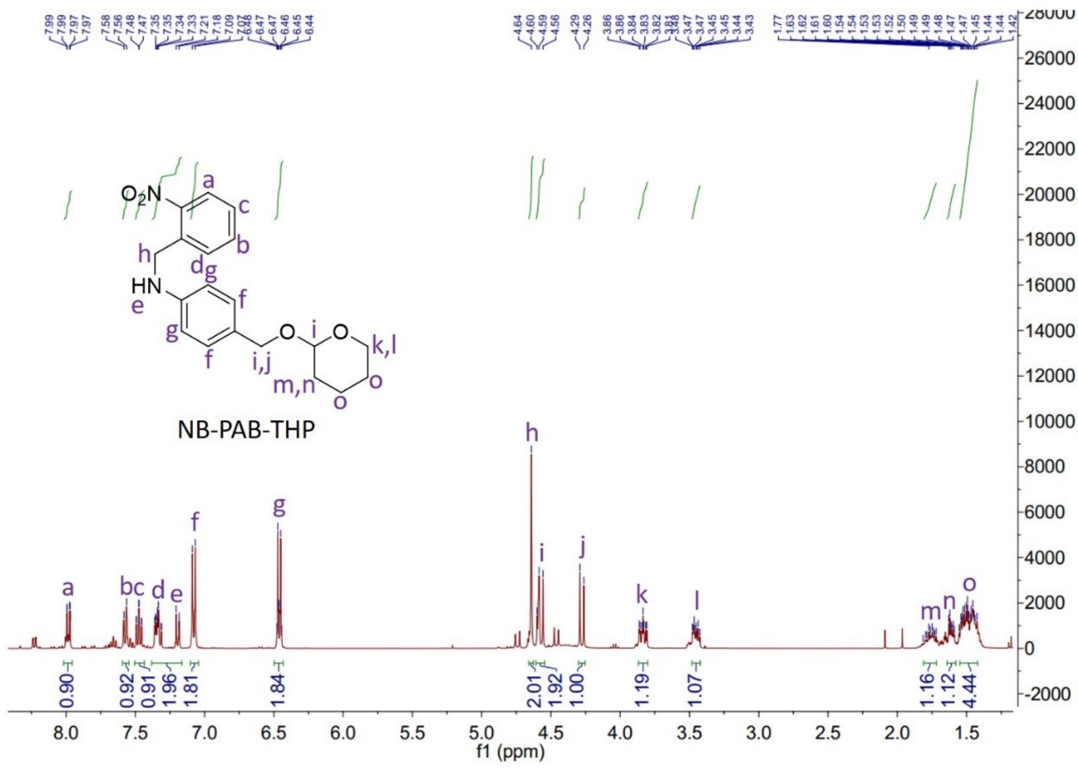

Figure S59. ^{1}H NMR of NB-PAB-THP.

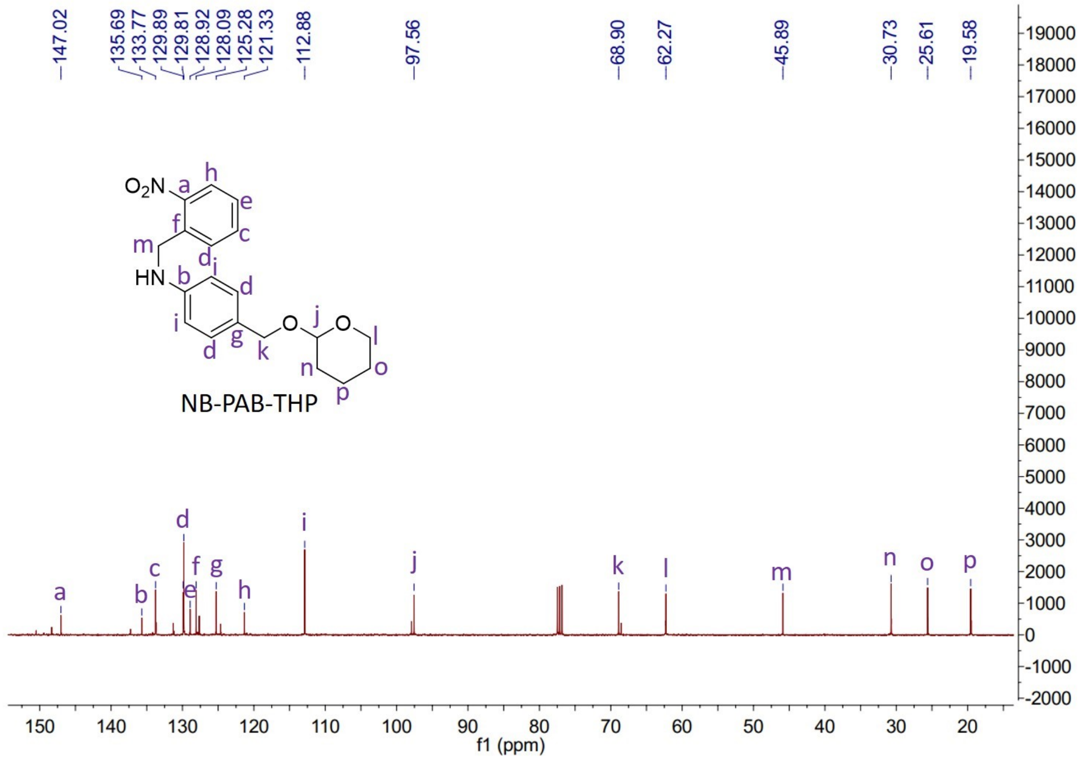

Figure S60. ^{13}C NMR of NB-PAB-THP.

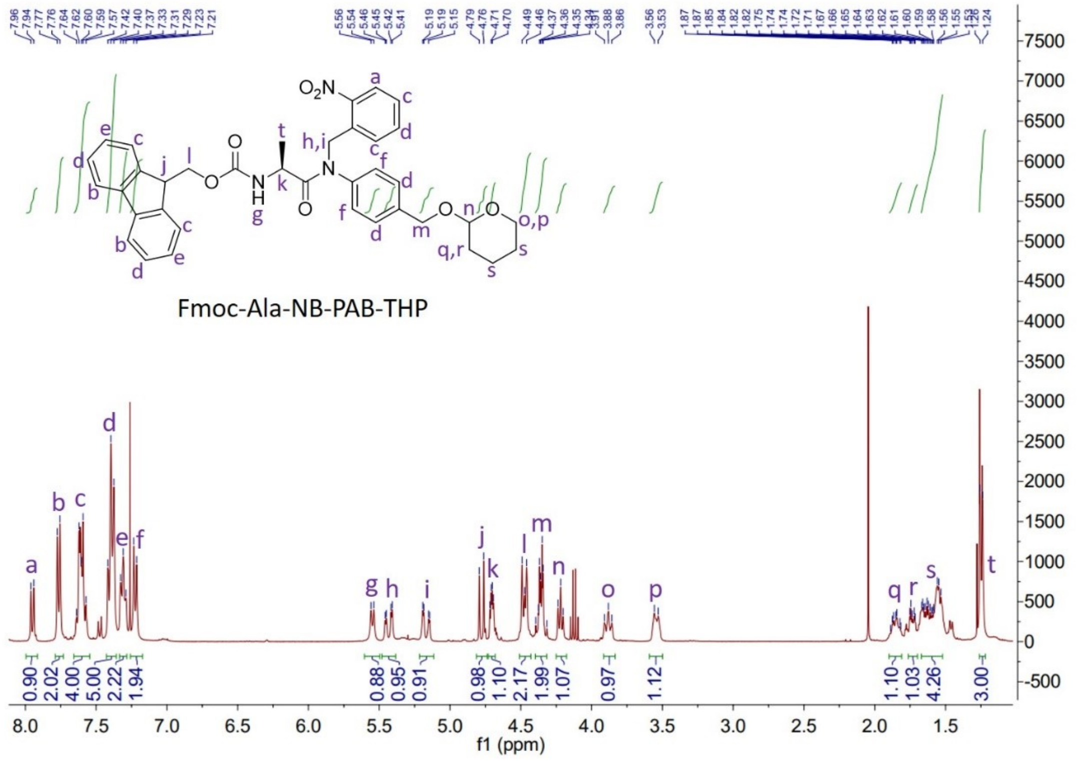

Figure S61. ^{1}H NMR of Fmoc-Ala-NB-PAB-THP.

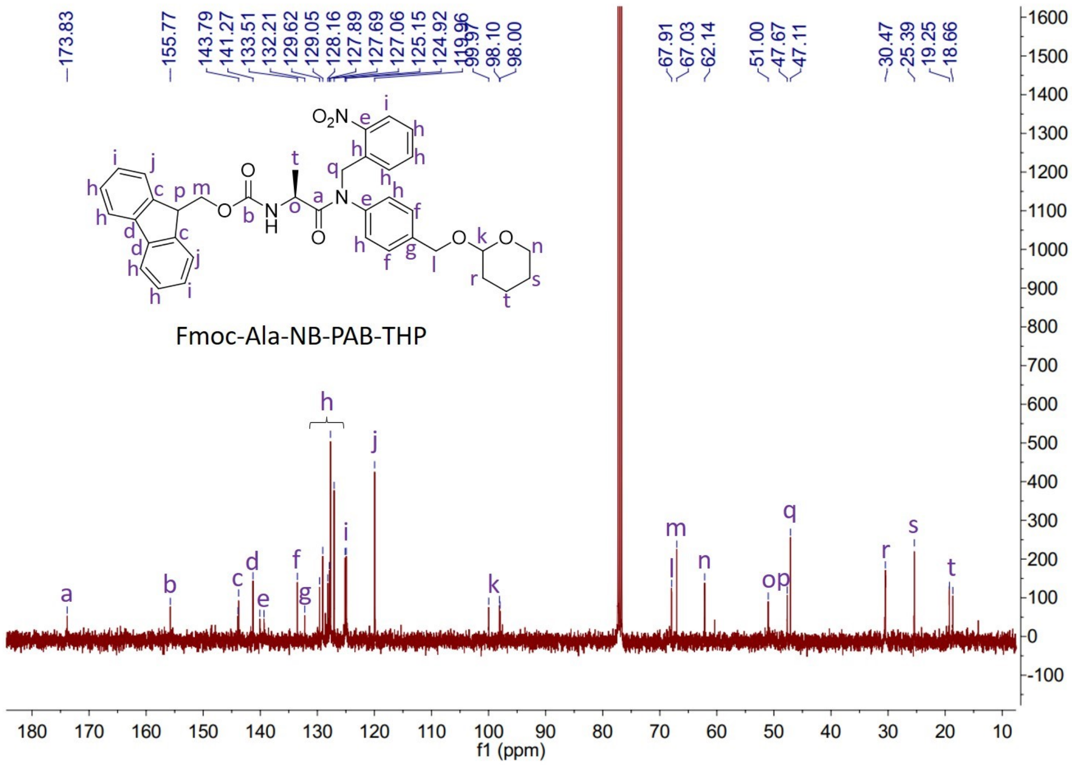

Figure S62. ^{13}C NMR of Fmoc-Ala-NB-PAB-THP.

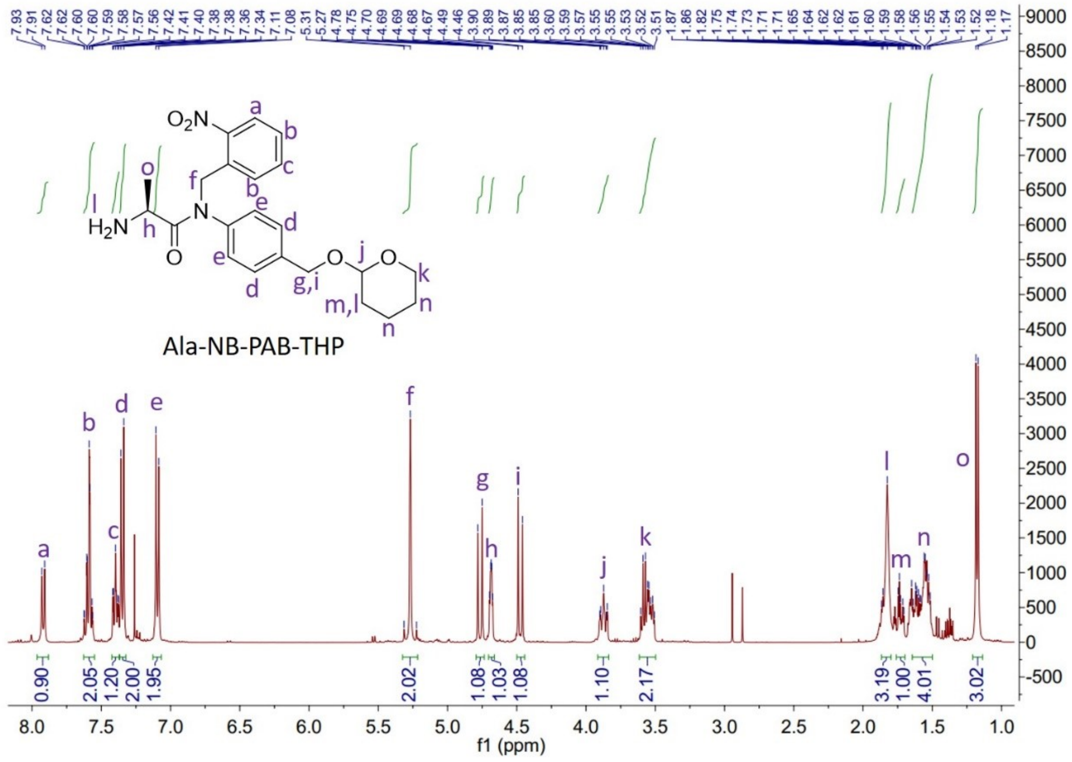

Figure S63. ^{1}H NMR of Ala-NB-PAB-THP.

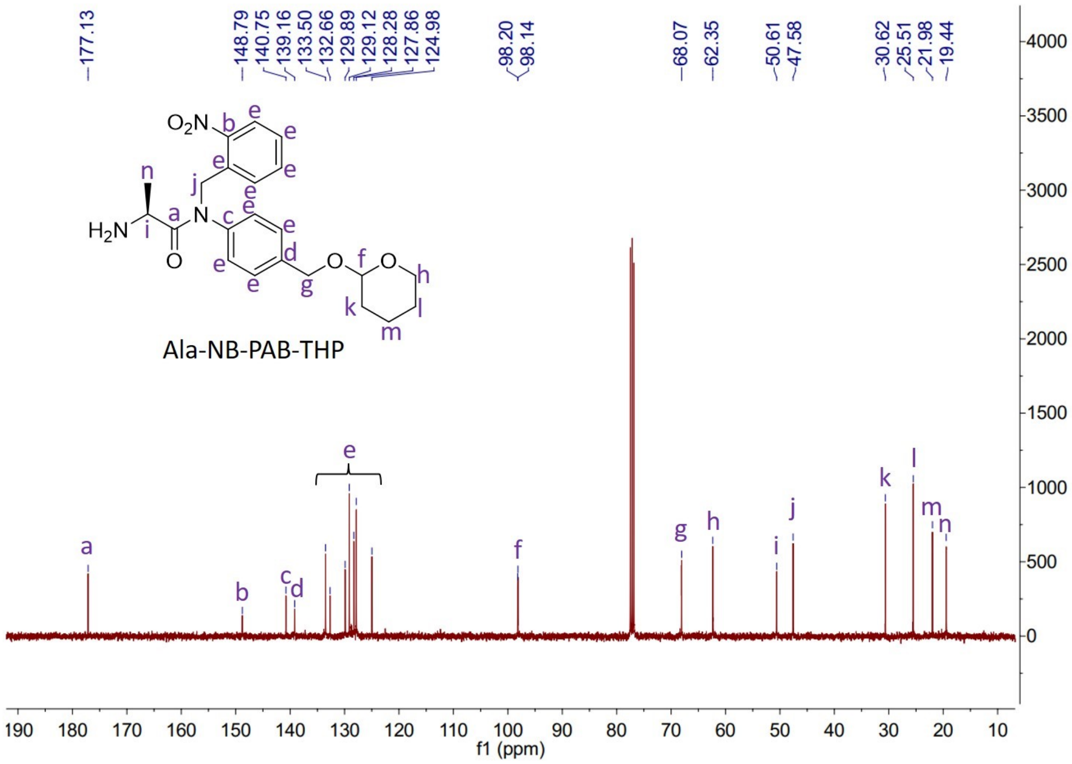

Figure S64. ^{13}C NMR of Ala-NB-PAB-THP.

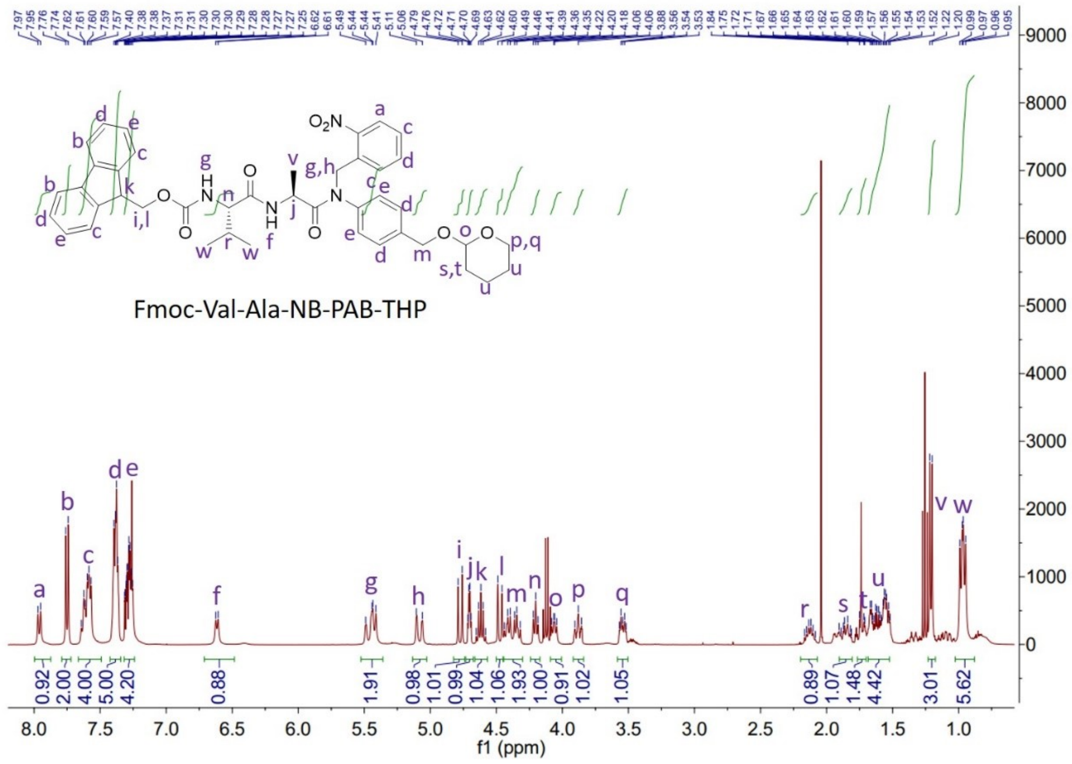

Figure S65. ^{1}H NMR of Fmoc-Val-Ala-NB-PAB-THP.

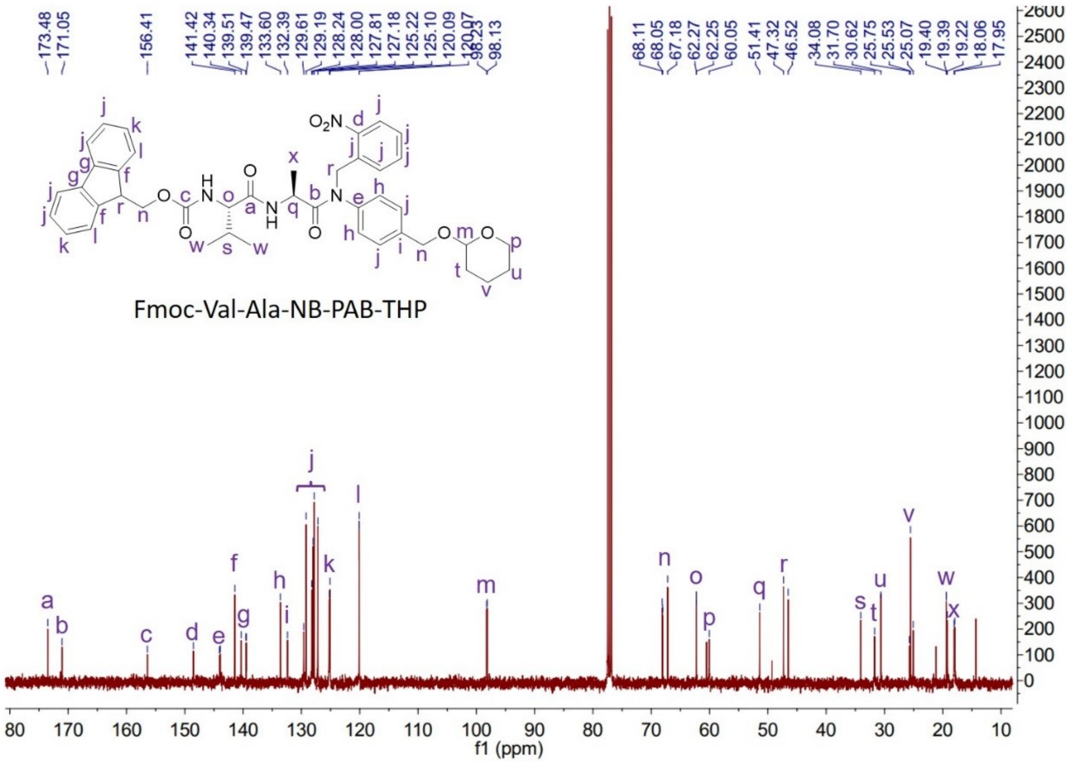

Figure S66. ^{13}C NMR of Fmoc-Val-Ala-NB-PAB-THP.

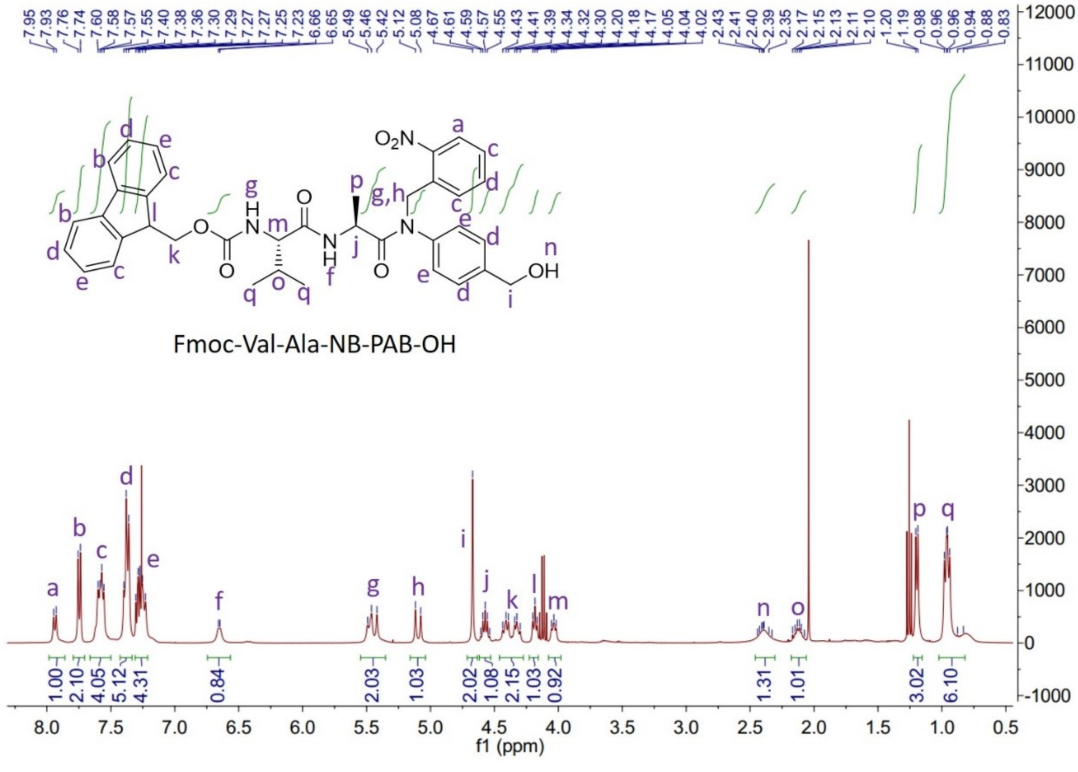

Figure S67. ^{1}H NMR of Fmoc-Val-Ala-NB-PAB-OH.

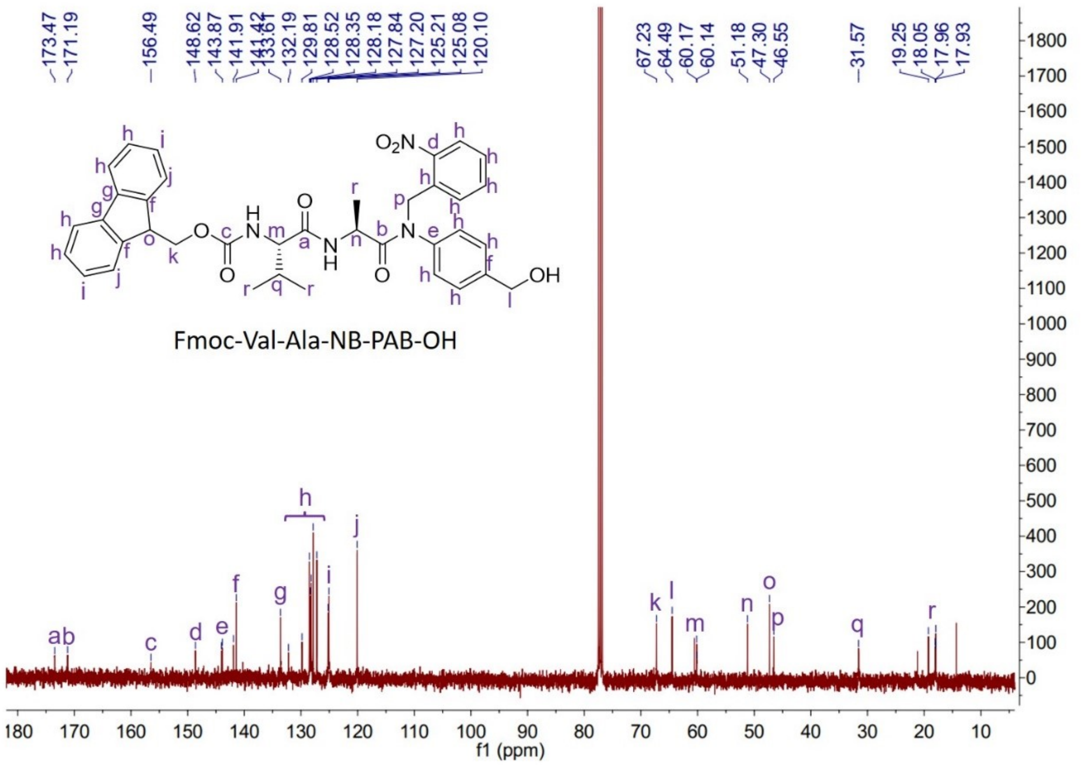

Figure S68. ^{13}C NMR of Fmoc-Val-Ala-NB-PAB-OH.

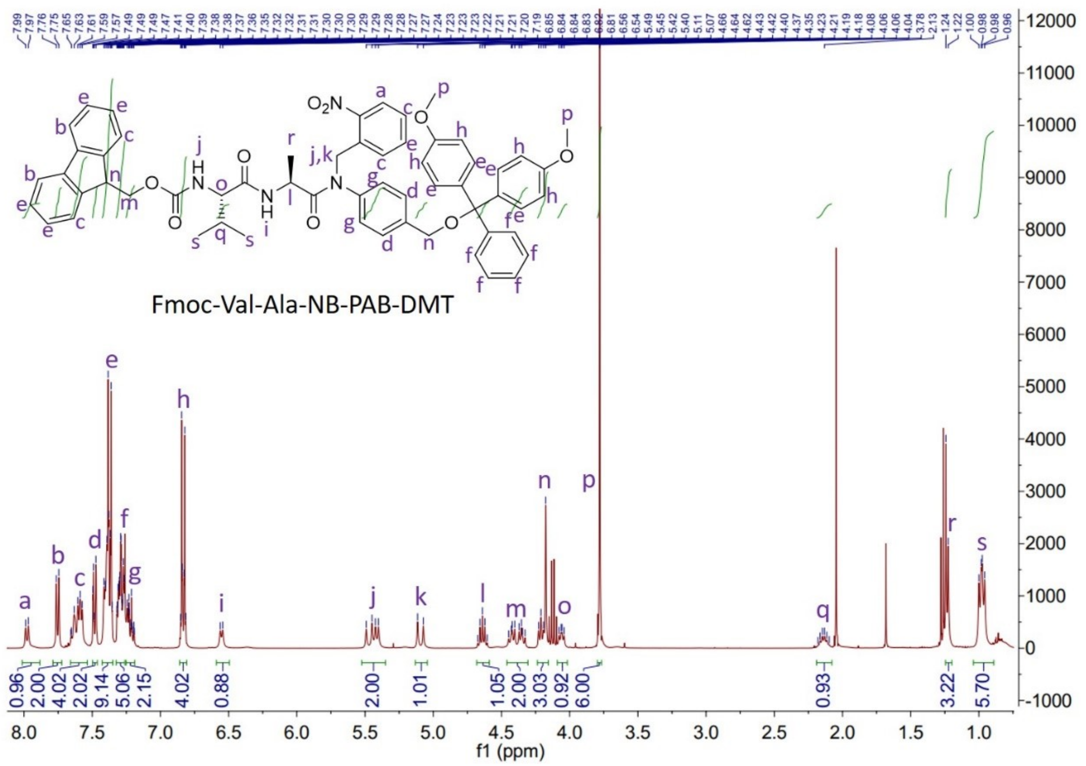

Figure S69. ^{1}H NMR of Fmoc-Val-Ala-NB-PAB-DMT.

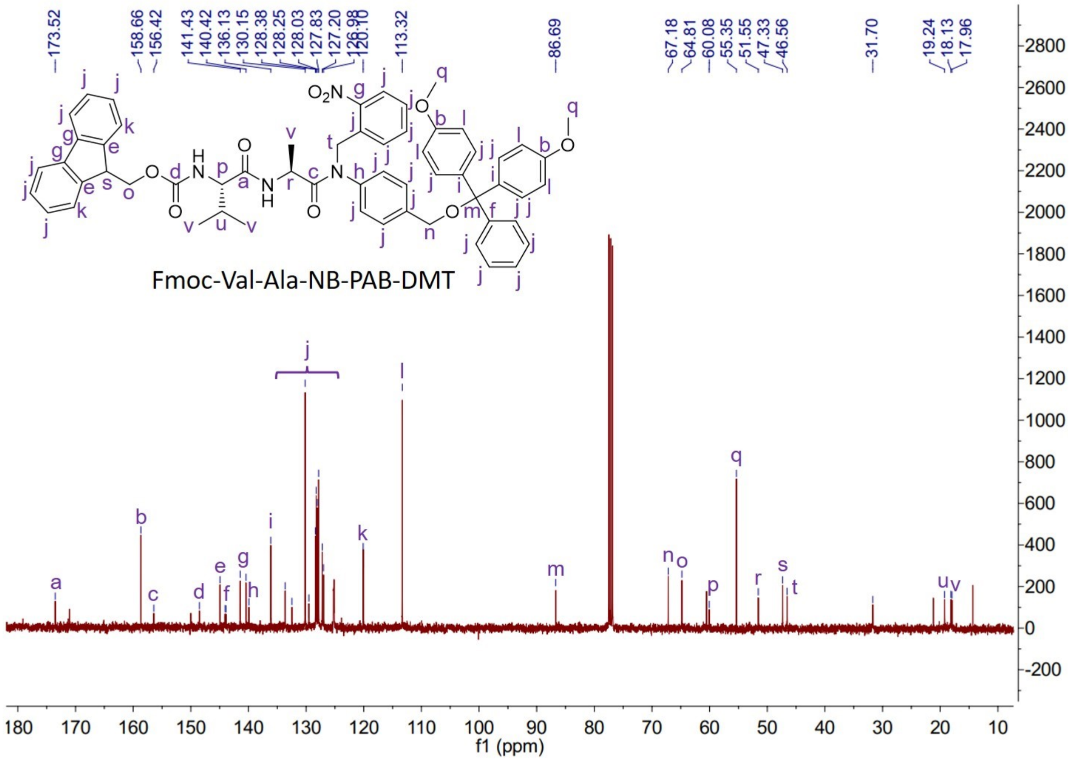

Figure S70. ^{13}C NMR of Fmoc-Val-Ala-NB-PAB-DMT.

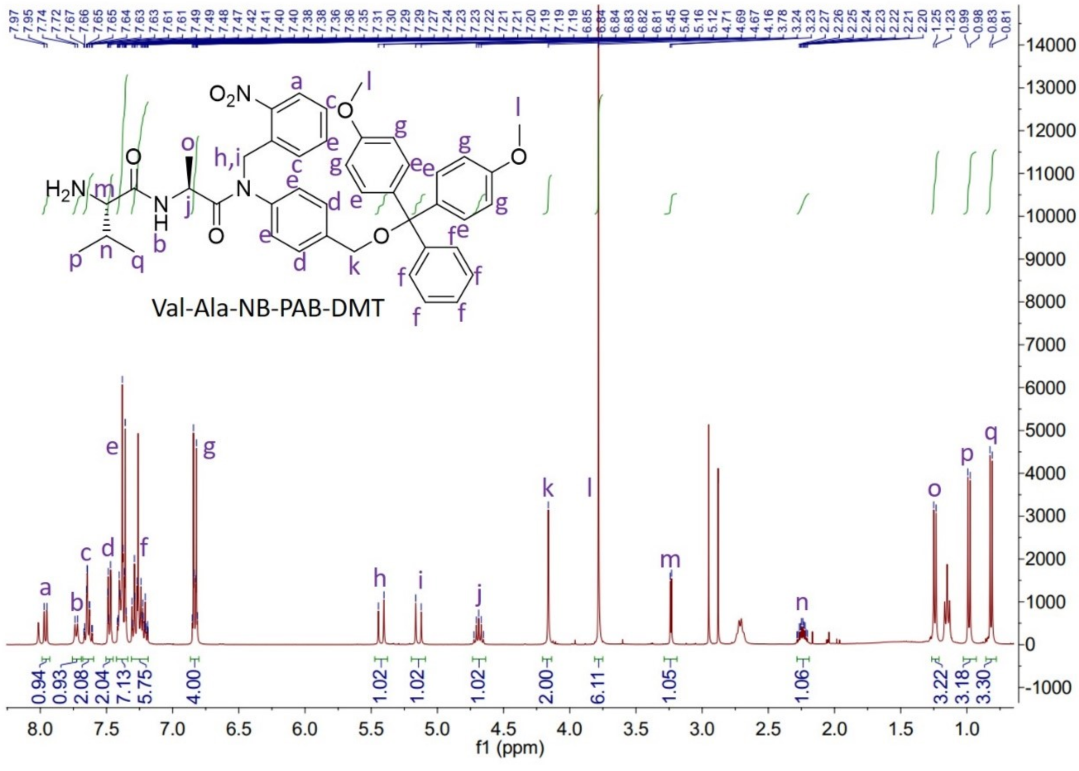

Figure S71. ^{1}H NMR of Val-Ala-NB-PAB-DMT.

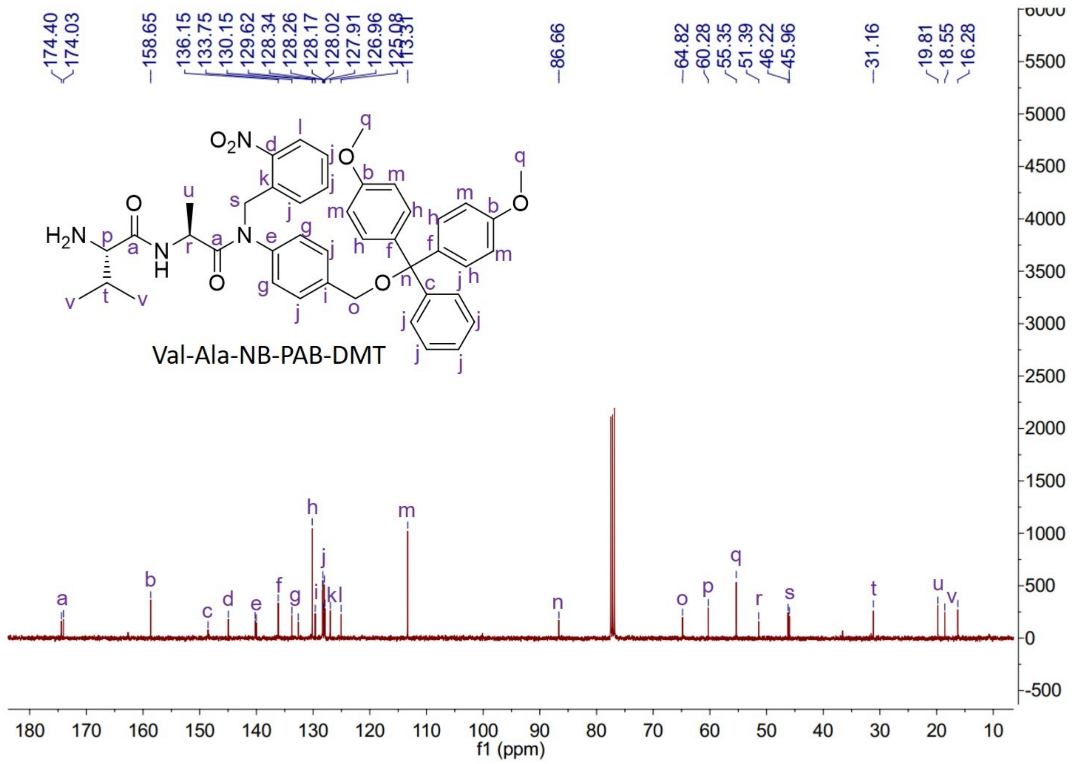

Figure S72. ^{13}C NMR of Val-Ala-NB-PAB-DMT.

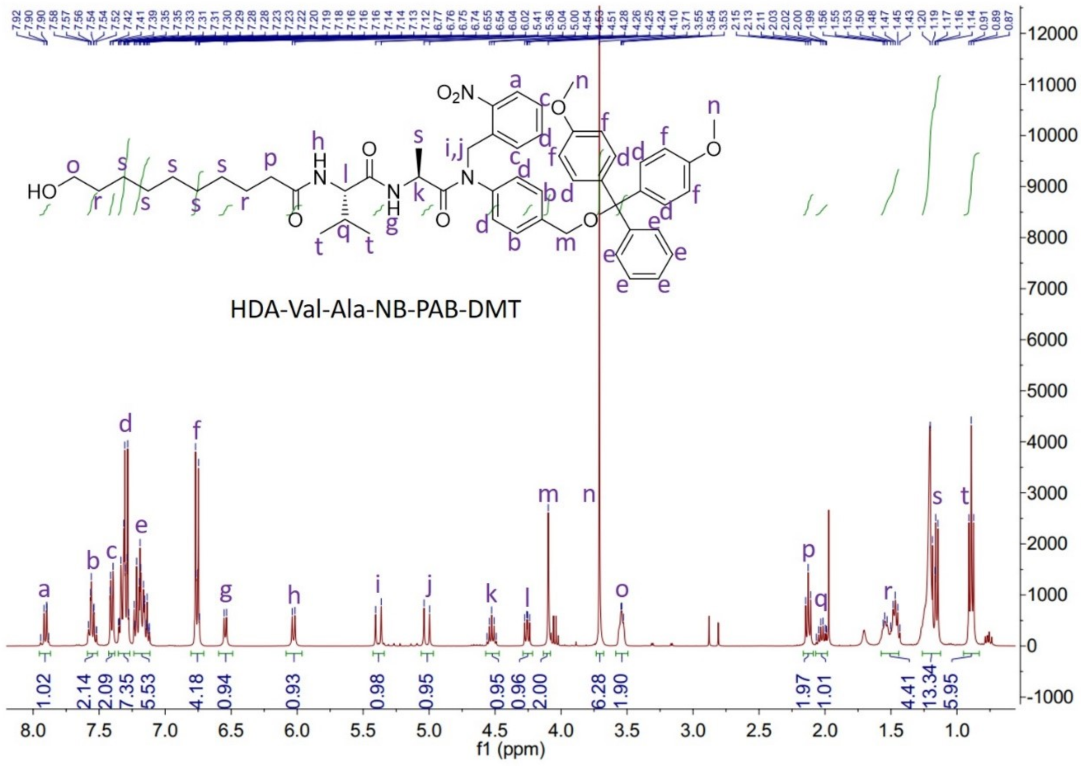

Figure S73. ^{1}H NMR of HDA-Val-Ala-NB-PAB-DMT.

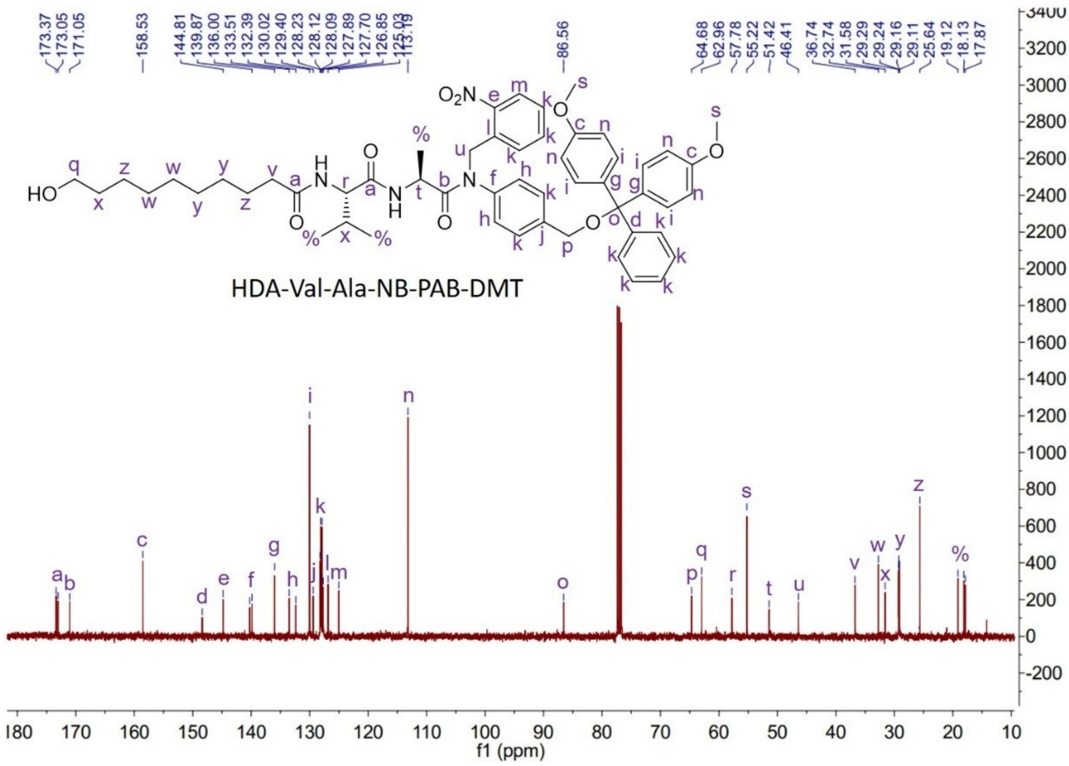

Figure S74. ^{13}C NMR of HDA-Val-Ala-NB-PAB-DMT.

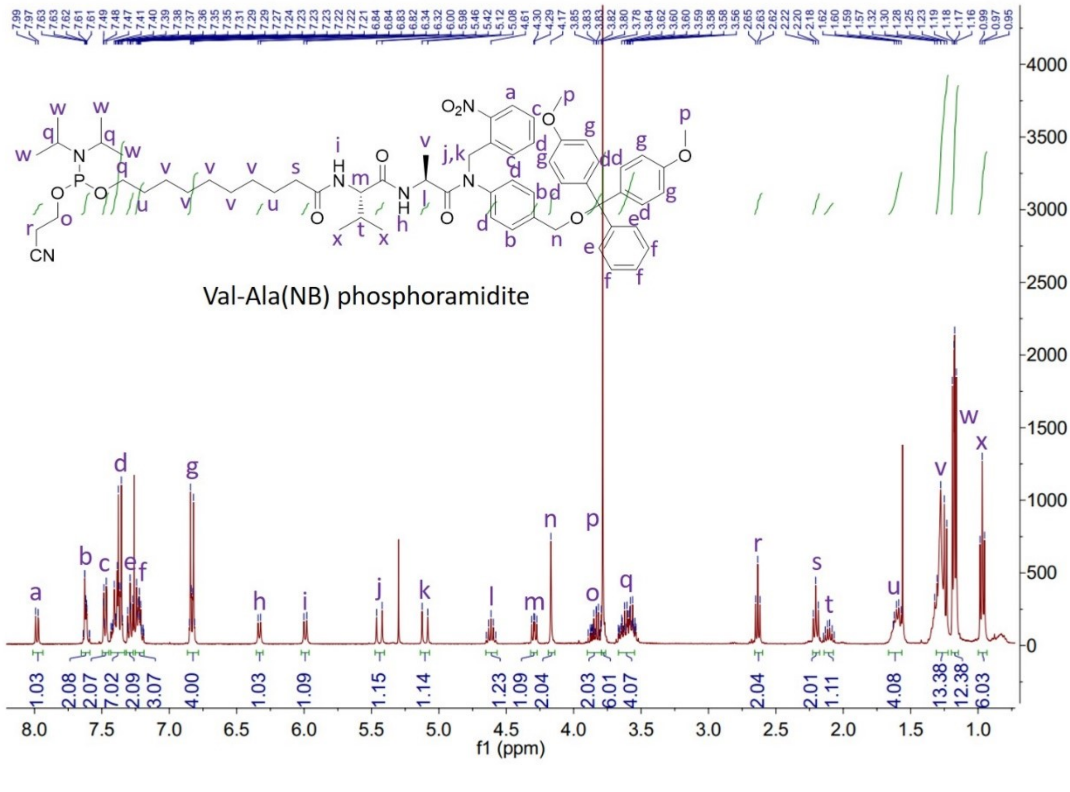

Figure S75. ^{1}H NMR of Val-Ala(NB) phosphoramidite.

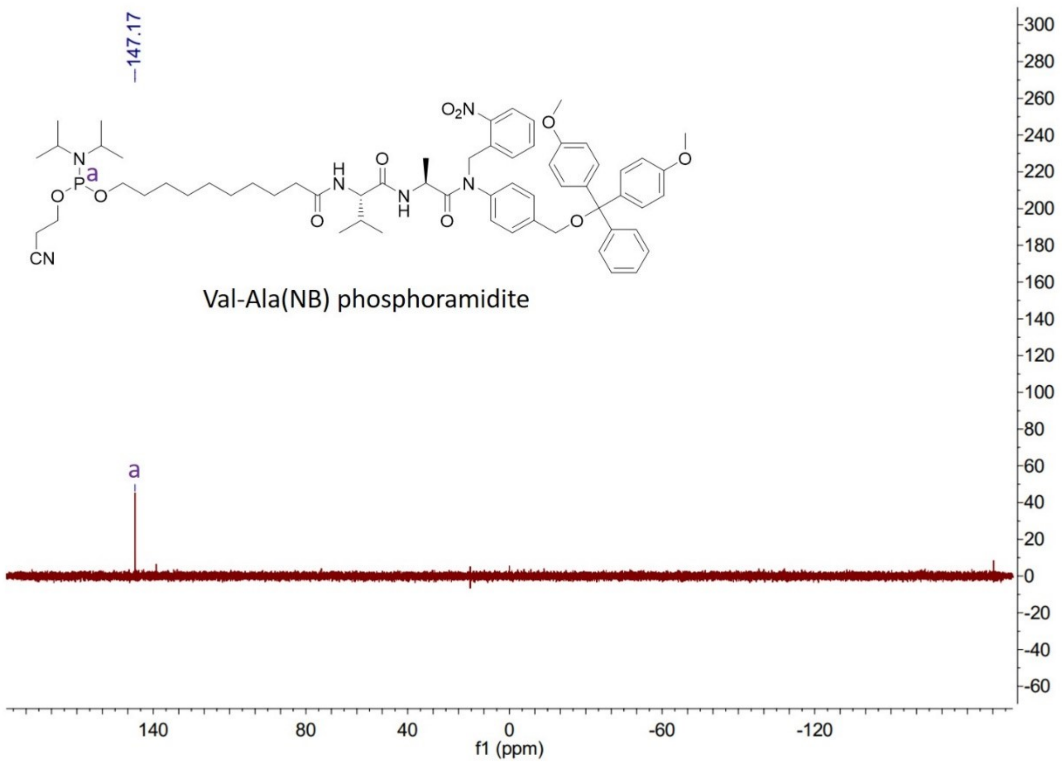

Figure S76. ^{31}P NMR of Val-Ala(NB) phosphoramidite.

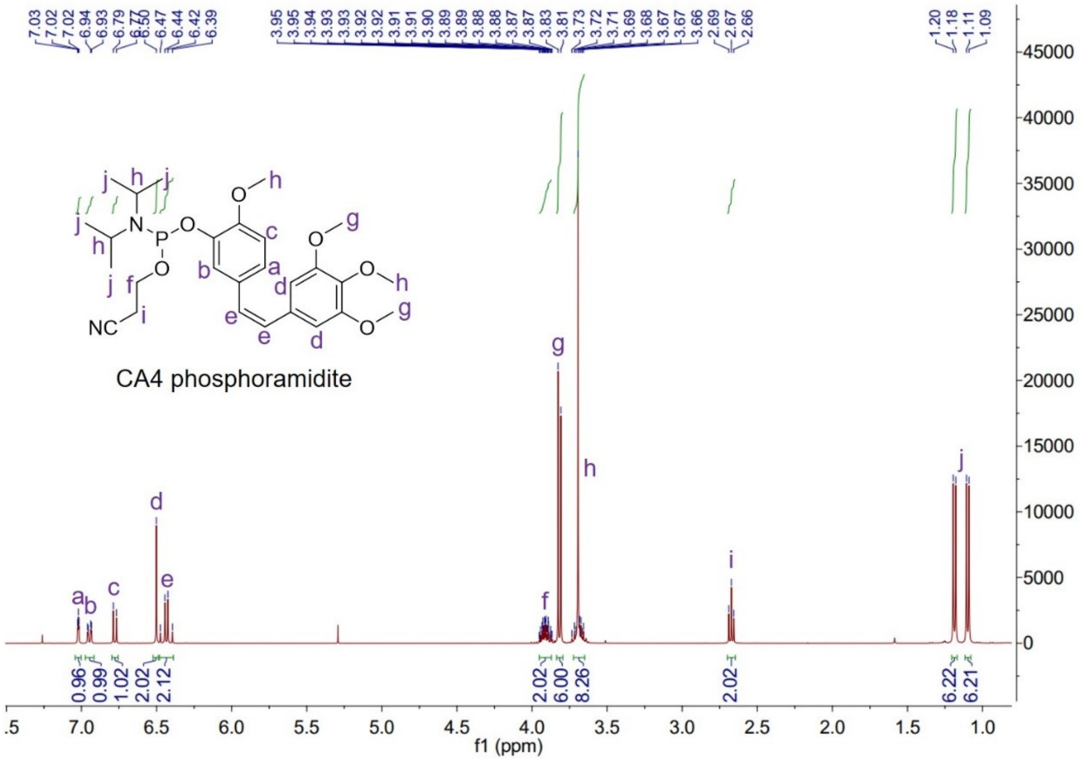

Figure S77. ^{1}H NMR of CA4 phosphoramidite.

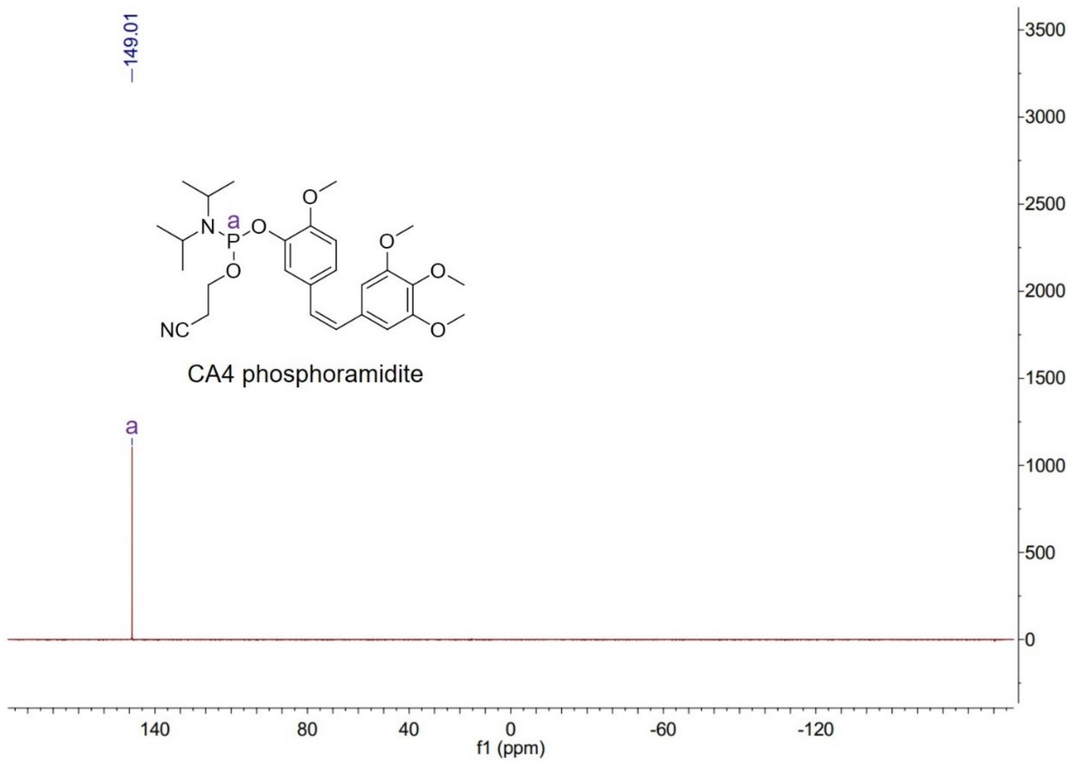

Figure S78. ^{31}P NMR of CA4 phosphoramidite.

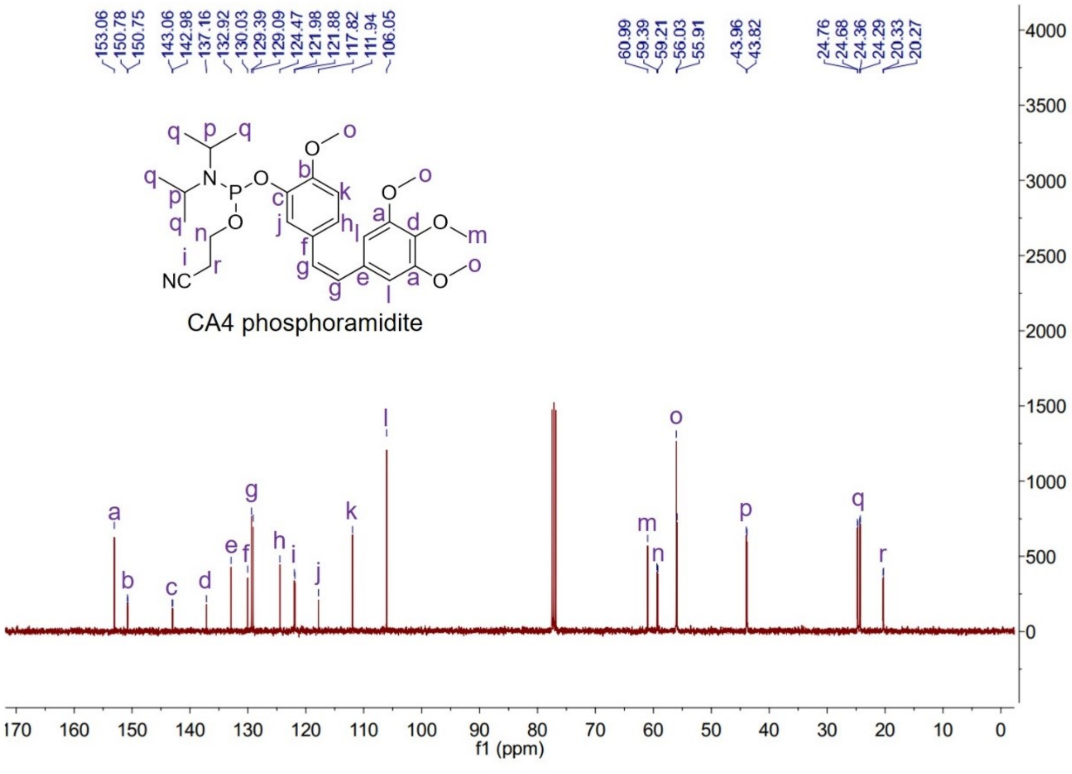

Figure S79. ^{13}C NMR of CA4 phosphoramidite.

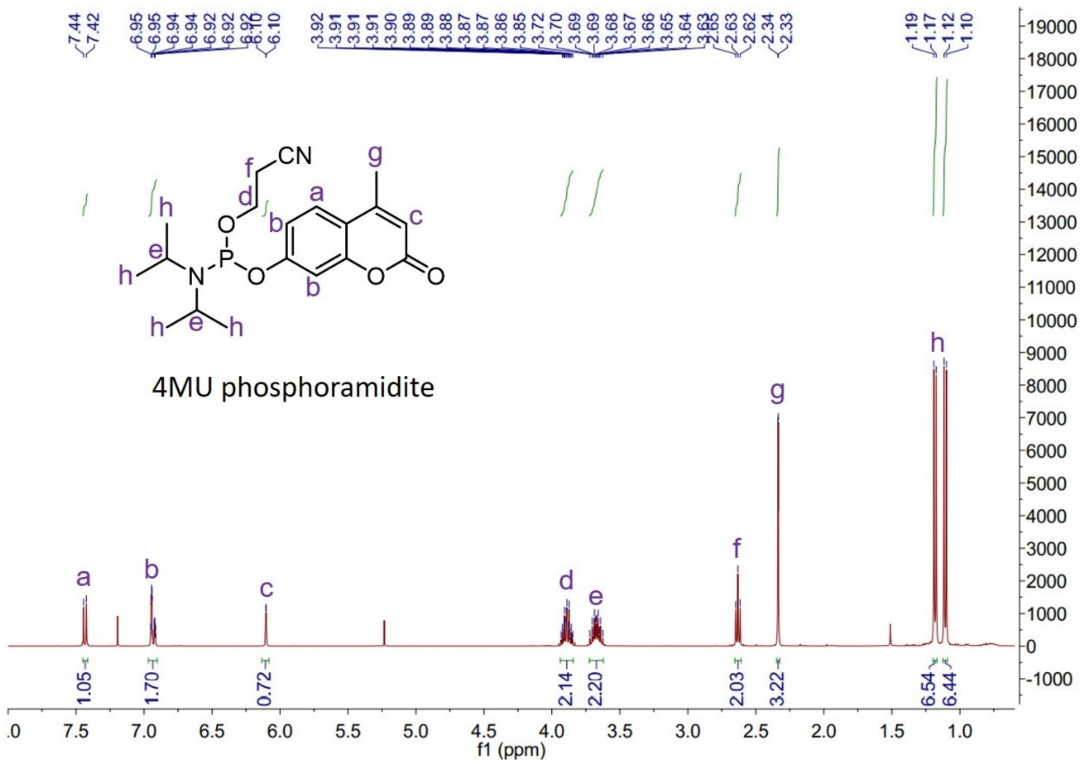

Figure S80. ^{1}H NMR of 4MU phosphoramidite.

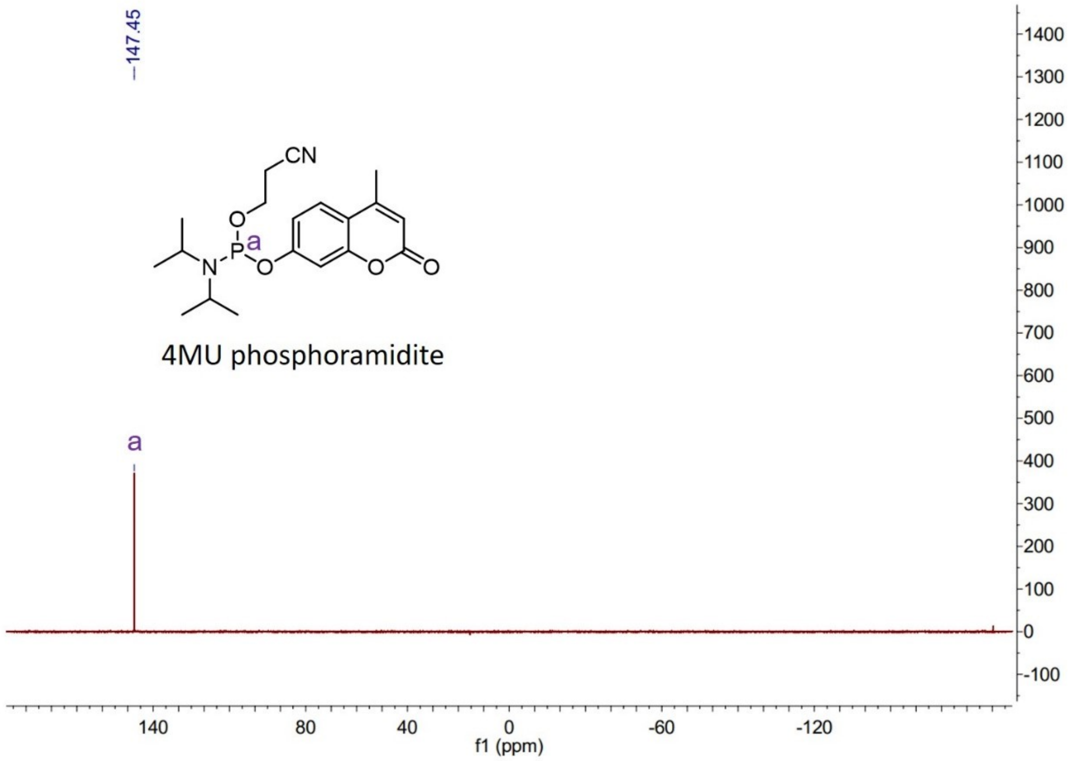

Figure S81. ^{31}P NMR of 4MU phosphoramidite.

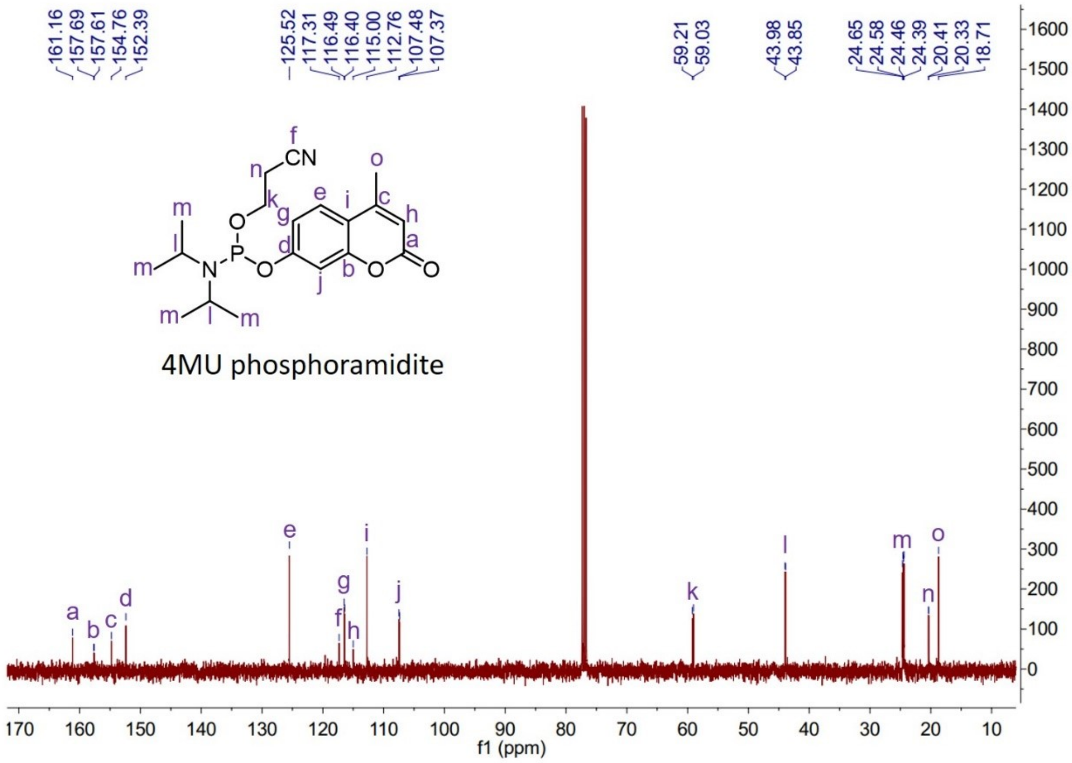

Figure S82. ^{13}C NMR of 4MU phosphoramidite.

6. References

1. C. Jin, A. H. Ei-Sagheer, S. Q. Li, K. A. Vallis, W. H. Tan and T. Brown, *Angew. Chem. Int. Ed.*, 2022, **61**, e202114016.
